# Differences in Performance of ASD and ADHD Subjects Facing Cognitive Loads in an Innovative Reasoning Experiment

**DOI:** 10.3390/brainsci11111531

**Published:** 2021-11-18

**Authors:** Anastasia Papaioannou, Eva Kalantzi, Christos C. Papageorgiou, Kalliopi Korombili, Anastasia Bokou, Artemios Pehlivanidis, Charalabos C. Papageorgiou, George Papaioannou

**Affiliations:** 1 1st Department of Psychiatry, Eginition Hospital, Medical School, National University of Athens, 11528 Athens, Greece; kalantzieva@yahoo.gr (E.K.); lina.korompili@gmail.com (K.K.); anastasia.bokou@hotmail.com (A.B.); apechlib@med.uoa.gr (A.P.); chpapag@med.uoa.gr (C.C.P.); 2 Neurosciences and Precision Medicine Research Institute “COSTAS STEFANIS” (UMHRI), University Mental Health, Papagou, 15601 Athens, Greece; 3 251 Air Force General Hospital, 11525 Athens, Greece; chrispapageorgio@gmail.com; 4 Center for Research of Nonlinear Systems (CRANS), Department of Mathematics, University of Patras, 26500 Patra, Greece; gpthespies@gmail.com

**Keywords:** multiscale entropy, Partial Least Square Correlation PLSC, Aristotle’s syllogism, ASD-ADHD, cognitive load

## Abstract

We aim to investigate whether EEG dynamics differ in adults with ASD (Autism Spectrum Disorders) and ADHD (attention-deficit/hyperactivity disorder) compared with healthy subjects during the performance of an innovative cognitive task, Aristotle’s valid and invalid syllogisms, and how these differences correlate with brain regions and behavioral data for each subject. We recorded EEGs from 14 scalp electrodes (channels) in 21 adults with ADHD, 21 with ASD, and 21 healthy, normal subjects. The subjects were exposed in a set of innovative cognitive tasks (inducing varying cognitive loads), Aristotle’s two types of syllogism mentioned above. A set of 39 questions were given to participants related to valid–invalid syllogisms as well as a separate set of questionnaires, in order to collect a number of demographic and behavioral data, with the aim of detecting shared information with values of a feature extracted from EEG, **the multiscale entropy (MSE)**, in the 14 channels (‘brain regions’). MSE, a nonlinear information-theoretic measure of complexity, was computed to extract a feature that quantifies the complexity of the EEG. **Behavior-Partial Least Squares Correlation, PLSC**, is the method to detect the correlation between two sets of data, brain, and behavioral measures. **-PLSC**, a variant of PLSC, was applied to build **a functional connectivity** of the brain regions involved in the reasoning tasks. **Graph-theoretic measures** were used to quantify the complexity of the functional networks. Based on the results of the analysis described in this work, a mixed 14 × 2 × 3 ANOVA showed significant **main effects of** **group factor** and brain region* syllogism **factor**, as well as a significant brain region* group interaction. **There are significant differences between the means of MSE (complexity) values at the 14 channels of the members of the ‘pathological’ groups of participants, i.e., between ASD and ADHD, while the difference in means of MSE between both ASD and ADHD and that of the control group is not significant. In conclusion, the valid–invalid type of syllogism generates significantly different complexity values, MSE, between ASD and ADHD. The complexity of activated brain regions of ASD participants increased significantly when switching from a valid to an invalid syllogism, indicating the need for more resources to ‘face’ the task escalating difficulty in ASD subjects. This increase is not so evident in both ADHD and control.** Statistically significant differences were found also in the behavioral response of ASD and ADHD, compared with those of control subjects, based on the principal brain and behavior saliences extracted by PLSC. Specifically, two behavioral measures, the emotional state and the degree of confidence of participants in answering questions in Aristotle’s valid–invalid syllogisms, and one demographic variable, age, statistically and significantly discriminate the three groups’ ASD. The seed-PLC generated **functional connectivity networks** for ASD, ADHD, and control, were ‘projected’ on the regions of the **Default Mode Network (DMN)**, the ‘reference’ connectivity, of which the structural changes were found significant in distinguishing the three groups. The contribution of this work lies in the examination of the relationship between brain activity and behavioral responses of healthy and ‘pathological’ participants in the case of cognitive reasoning of the type of Aristotle’s valid and invalid syllogisms, using PLSC, a machine learning approach combined with MSE, a nonlinear method of extracting a feature based on EEGs that captures a broad spectrum of EEGs linear and nonlinear characteristics. The results seem promising in adopting this type of reasoning, in the future, after further enhancements and experimental tests, as a supplementary instrument towards examining the differences in brain activity and behavioral responses of ASD and ADHD patients. The application of the combination of these two methods, after further elaboration and testing as new and complementary to the existing ones, may be considered as a tool of analysis in helping detecting more effectively such types of disorders.

## 1. Introduction

Model-based inferences in human neuroscience is an emerging, modern, and very promising field. Relationships between neuroimaging measures and behavior give extremely valuable and important evidence about brain function and cognition in both healthy and ‘pathological’ populations. The usage of electroencephalography (EEG) (a portable, low cost measure of brain dynamics), within the framework of the new field is however limited. In this paper, we applied the *behavior-PLSC and the seed models* [1], to generate inferences about brain activity-behavior interaction, contributing towards bridging the aforementioned gap. Other similar methods are Canonical Correlation Analysis, CCA, and Hierarchical Bayesian models, HBM. The knowledge that is acquired by using such models mentioned before, will help us understand how individual differences in the brain contribute to individual differences in cognitive processes, especially in the case of ‘pathological’ groups of subjects as ASD and ADHD that are examined in the present work. We combine PLSC analysis in our work with a nonlinear method for deriving a feature from the EEG, the multiscale entropy, MSE, a measure of complexity, in order to enhance the signal since MSE captures a spectrum of characteristics related to nonlinear interaction of brain neurons during cognitive processes, induced in our case by a special type of reasoning, Aristotle’s syllogism.

We examine the complexity of participants’ EEG activity and their behavioral performance at multiple levels of difficulty imposed by the valid–invalid syllogisms. Participant’s response is reflected via their linear and nonlinear patterns in dealing with the different cognitive loads due to this type of syllogism. In other typical types of reasoning or cognitive tasks, such as the well-known *N-back tasks*, linear and nonlinear load effects are shown in participants [2]. Therefore, a natural question that came to our mind was the following: *Are the induced cognitive loads by Aristotle’s valid–invalid syllogisms of a similar type to the ones induced by a typical N-back task? If yes, how the three groups of participants in the experiment are expected to respond? How the ‘transition’ from a valid to invalid syllogism is perceived by an ASD or ADHD participant in comparison with a participant of the control group?*

Answers to these and related questions are provided in this work, which are expected to contribute both theoretically and practically to the current literature, by shading light on the understanding of brain dynamics and behavioral dynamics interaction and how this interaction affects the instantaneous cognitive functional connectivity in the brain networks of healthy and ASD-ADHD subjects.

A behavioral- and functional connectivity-oriented application of PLSC is followed in this paper, to examine the relationship between brain activity and behavior. Typical EEGs and values of entropy extracted from EEGs were used to form the *brain activity matrix*. Behavioral responses of the participants form the *behavioral matrix*, as describe below. Our work is inspired by a number of ‘similar’ research efforts, in respect of purpose and methods used. The most recent work is that of Meidenbauer et al. [3], in which the authors conducted a behavioral PLSC to examine the interaction between functional near-infrared (fNIRS) activation and behavioral responses, in an experiment aiming at revealing the load-dependent activation in prefrontal and parietal cortex during working memory task, such as the N-back one. They provide evidence, using the PLS analysis, that there are significant differences in the correlation between accuracy in participants’ responses and changes in the dynamics of the fNIRS signals, as a function of N-back level, in eight mid-frontal channels. They demonstrated that by using the behavioral PLSC method, fNIRS activity can capture working memory loads that vary with the difficulty of N-back task, as well as significant associations between brain activity and behavioral performance (measured by accuracy and reaction time), contributing therefore to this kind of modality that seems to be a promising tool in various applications. Another work, related to this one is the work of Price et al. [4] in which voxel activity from PET scans of participants were used for creating the brain activity matrix *X* and descriptive (demographic) and cognitive measures, such as *age*, *white matter volume*, and *mental status* on the Mini-Mental State Examination, MMSE [5], were used for the *Y* behavioral matrix. Their aim was to examine the shared information (or relationship) between *X* and *Y* of individuals with Alzheimer’s disease and normal controls. Other indicative works, related to the present paper, using PLSC is the work of [6], to examine the correlation between gray matter systems and response inhibition in obsessive compulsive disorder. Also, the work of [7] to examine the relationship between brain structure and neuropsychological tests in schizophrenia and, finally, the paper of [8] to examine how the brain shape relates to neuropsychological behavior and cortical functioning in the case of patients with traumatic brain injury.

To clarify further how this work resembles the above-mentioned efforts, as far as the structure of the most critical parameters of the PLSC method is concerned, we note that our brain dynamics matrix *X* consists of MSE values extracted from EEGs, while the behavioral data *Y* is formed by *age*, *emotional state* (measured by Self-Assessment Manikin, SAM) and *percent of confidence* of participants in answering the thirty-nine questions in the valid–invalid arguments of Aristotle, presented to them during the experiment. 

The main aim of the present work is to shed light on the connection between the changes in cognitive loads that are generated by two ‘special’ types of reasoning, namely *Aristotle’s valid and invalid types of syllogisms* with the behavioral responses of the participants, by using PLSC methods. More specifically, EEG recordings from *healthy (control) and ‘pathological’ subjects (ASD and ADHD)* are used as input to the nonlinear tool multiscale entropy (MSE), to extract a feature values that in turn are inputs to PLSC in order to examine how brain activity in different regions is correlated with behavioral responses of the subjects, due to their exposure in cognitive loads of varying difficulty induced by the ‘peculiarities’ of the Aristotelian syllogisms. In addition, we used seed-PLSC method to construct the functional connectivity networks, related to the above interactions, and compare them to detect any differences among the three groups.

This analysis, as a consequence, necessitates the examination of the *involvement or coupling* of the following ‘concepts’: (a) the complexity-variability of EEG signals (measured by MSE) of healthy and ‘pathological’ subjects (b) the connection of Aristotelian reasoning with primary cognitive processes (cognitive control, attention, working memory etc.) and cognitive features involved in Executive Function (EF), (c) the comparison of the correlation of the above cognitive processes with behavioral data, with the correlation found in this work between brain activity and behavioral data, and finally (d) the creation of functional connectivity networks related to interaction of brain regions and behavioral dynamics within the Default Mode Network, DMN, used a ‘reference’ network the changes of which are the criteria for distinguishing the three groups of participants The analysis of the above concepts will provide valuable information towards answering the question of whether or not the main induced cognitive processes in subjects exposed in Aristotelian syllogisms engage the same brain regions and exhibit similar modality with typical reasoning. 

As a small reference list of how cognitive concepts are relevant to the above coupling, we refer to the paper of Evans et al. [9], where they argue that primary cognitive processes are the outcome of activation of *cognitive networks that interact*, as the EEG signals reveal significant power in the theta and alpha brain rhythms. For example, increased frontal theta activity (event-related synchronization) is linked to cognitive control [10] and working memory [11], while the recruitment of attention is linked to decreased parietal alpha activity (event-related synchronization) [12]. Long-term memory is associated with increased parietal alpha activity [12]. A very interesting finding is that *novelty conflict, punishment and error* are associated with a cognitive *control under uncertainty* [10]. The mental effort in remembering increasing number of things, facts etc. is associated with *increased theta activity* with which maintenance and manipulation are also linked. Computational demands are linked to all components of working memory. Attention to a task-relevant information is associated with a decreased alpha activity. Access of knowledge systems (including long-term memory) is linked to increased alpha activity, a finding that is in compliance with the broadly accepted notion that *long-term memory is an automatic process* [13]. 

To the best of our knowledge, although a plethora of works exist using PLSC methods on fMRI, PET data, and very few papers exist for PLSC applications on EEG data, therefore the present study contributes also towards bridging this gap in the literature. Furthermore, the contribution of this work becomes more important because, also, only a very few works exist that analyze the brain–behavior dynamics interaction in ASD and ADHD patients based on EEG recordings. This research has revealed uncharacteristic ADHD-associated reports mainly in relation to the theta and beta oscillations [14,15,16,17] and uncharacteristic ASD-associated reports mainly in relation to the alpha, beta, and gamma oscillations [16,17,18]. The combine application of Behavior-PLSC and seed-PLSC to study the ‘primitive’ functional connectivity networks corresponding to these interactions, can also be considered as an innovative approach, adopted in the current study. The results seem promising in adopting this type of reasoning, in the future, after further enhancements and experimental tests (via using more sophisticated instruments of behavioral measures), as a supplementary instrument towards examining the differences in brain activity and behavioral responses of ASD and ADHD patients. 

The rest of this paper is structured as follows. In Section 1.2, the Aristotelian types of reasoning and their connection with typical cognitive processes is described. The link between syllogistic reasoning, linguistic and visuo-spatial systems, and Executive Function (EF) is given in Section 1.3. The concepts of complexity and MSE in normal (control) and ‘pathological’ conditions are presented in Section 1.4, in which we also provide a description of how ASD, and ADHD ‘manage’ the emotional regulation, which is one of the statistically significant behavioral measures found from ANOVA and parametric tests, that is used in the present work. This completes the introduction we believe to be a short, but concise literature review of all the key concepts used in the present work. Then, the materials and methods in Section 2 are described, detailing participants, EEG recording, the MSE computation, and finally the behavior-PLSC, seed-PLSC analysis, and the link of the later term with the brain functional connectivity networks, within the framework of the DMN network, the ‘reference’ brain system used as a ‘benchmark’ for distinguishing the ASD, ADHD and control group responses. Detailed results are given in Section 3, followed by an extensive discussion and a conclusion (Section 4).

### 1.1. The Structure of Aristotle’s Syllogism and Its Relation to Cognitive Processes

We briefly introduce at this point the syllogistic reasoning task and orthodox Aristotelian classification. This information is considered necessary in order to interpret easily the cognitive loads that the Aristotelian syllogisms impose on subjects trying to ‘handle’ them during experiments. Syllogisms are constructed with two premises and one conclusion. Each preposition or statement belongs to a group of four forms called *moods* [19,20]. These moods, traditionally, are labeled A, I, E, and O, as below [21]:

A: All *X* are *Y*
I: Some *X* are *Y*
E: No *X* is *Y*
O: Some *X* are not *Y*

The subject (S) (in a preposition) and predicate (P) in the conclusion are called *end terms,* while a term not present in the conclusion is a *middle term (M).* There are four arrangements or possibilities of end and middle terms, since each premise has two possibilities. The four possibilities mentioned above are called *figures.* Therefore, there are 4 × 4 × 4 = 64 possible types of premises for logical syllogisms, because each of two premises can be one of the four moods and four possibilities regarding the location of terms. Only 19 syllogisms out of 64 have a logical conclusion (they are *valid*), even though the validity of a syllogism is a relatively controversial concept [21]. Reasoning is intended to derive reasonable conclusions from premises and is carried out in working memory. Human *performance on syllogistic reasoning* is based on mental representations, as the mental model theory (one of the earliest comprehensive psychological theories of syllogism) explain [22]. As the authors in this paper claim, the *difficulty of syllogistic reasoning is a function of the number of mental models that must be constructed to derive a logically valid conclusion.* Also, a task that requires more models to be constructed to reach a correct answer, increases the probability of errors in the inference process, resulting possibly in a failure. As the mental model theory claims, the difficulty of (or cognitive load exerted in) syllogisms is determined primarily by the number of models and the Aristotelian *figure* (one of the four likely possibilities mentioned above). Mental model theory of syllogistic reasoning, incorporates explicitly the *working memory capacity*, an important component of cognitive process [23]. According to *Sample Mental Model (SMM),* a probabilistic approach in a mental representation [21], *people ‘use’ or sample six or seven instances in working memory to derive a conclusion to a syllogism.* However, [24] argue that the *limit of working memory capacity* is actually three to five chunks (‘fat pieces’), and this limit reflects human’s capacity for attention and constraints the relational representations, enabling making inferences.

All the preceding information lead to the conclusion that the ‘architecture’ or structure of Aristotelian syllogisms, reflected by modes and figures, in combination with the difficulty of syllogistic reasoning (which depends on the number of mental models that must be constructed before reaching a valid conclusion) and the limited capacity of working memory constraining the ability of working inferences, may be considered as the sources of cognitive loads, exerted on participants that are tested in Aristotelian syllogisms, which are responsible for shaping the dynamics of the EEGs recorded during relevant experiments. The aforementioned sources of cognitive loads may be located at, and activated by different brain regions, with different ways depending on task conditions or ‘pathological’ condition of a subject. It is challenging therefore to examine how normal, ASD and ADHD subjects ‘react’ when facing Aristotelian valid and invalid syllogisms, since the loads these syllogisms exert on subjects belonging to groups previously referred, differ as described above. Aristotle’s method of deduction is probably the first to analyze the logical reasoning or syllogism (also called **valid reasoning**). In his famous work ‘ORGANON—Prior analytics’ [19,20], the great philosopher presents a series of statements (the ‘building blocks’) in the process of reasoning that leads to a valid conclusion with absolute certainty. The **dual processing** model for the logical reasoning [25,26,27], is developed to help understand how the brain functions when a subject is ‘engaged’ in this type of reasoning. A natural question that is generated is whether Aristotle’s valid and invalid syllogism induce the same or different mental processes in the brain. This is a current, still open, and challenging research question, aiming at shedding light on the fundamental operation of reasoning, in its two extreme conditions. A work related to the study of EEG activity in different brain regions in the case of healthy participants exposed in valid and paradoxes syllogisms has been analyzed in the work of our team [28,29,30].

### 1.2. Syllogistic Reasoning, Linguistic, and Visuo-Spatial Systems and Executive Function (EF)

Goel et al. [31] provide strong evidence that syllogistic reasoning implicates a widespread network involving occipital, temporal, and parietal lobes, prefrontal cortex, and surprisingly, cerebellum and basal ganglia nuclei. According to Goel et al. [31], syllogistic reasoning is implemented in two distinct systems, whose engagement is mainly a function of the presence or absence of semantic content. In fact, the *temporal* system (left hemisphere, LH) is recruited during *content-based* reasoning, while the *parietal* system is activated when the reasoning lacks semantic content. The two systems however, share common components (bilateral basal ganglia nuclei, right cerebellum, bilateral fusiform gyri, and left prefrontal cortex). In addition, the *right prefrontal cortex* is recruited when logical argument results in a *belief–logic conflict*. What is doubtable in Goal et al.’s work, as Knauff argues [32], is the conclusion these authors draw from their findings. Specifically, their conclusion is that the *frontal-temporal system* is more *‘basic’*, and effortlessly engaged, while the parietal system is effortfully engaged only when the frontal-temporal route is due to a lack of familiar content. Under the current perspective, the question of how mental logical reasoning is implemented in the human brain is a question of *formal reasoning*. Goel et al. [31] and Knauff [32] argue that in syllogistic reasoning, both linguistic and visuo-spatial systems are engaged. Frontal cortex place a central role in logical reasoning. Since Aristotelian Syllogism ‘belongs’ to the model-based reasoning theoretical context, which suggests that reasoning is a visuo-spatial process, it is natural to deduce that parietal and occipital cortices are essential brain structures for Aristotelian syllogism. More specifically, Goel et al. [31] used problems with semantic content (e.g., All A are B; all B are C; so all A are C). At this point, we attempt to connect the aforementioned syllogistic reasoning, i.e., the cognitive loads that this induce on the executive function, especially of ‘pathological’ groups. The involvement of frontal and parieto-occipital regions in Aristotelian syllogism and Zeno-like paradoxes, respectively, has been found in the work of Papageorgiou et al. [30]. More specifically, event-related potential (ERP), generated by Zeno-like paradoxes, which mobilize frontal attention mechanisms, were compared with ERPs promoted by valid deduction in parietal-occipital regions associated with attention and memory processes.

ADHD and ASD are both developmental disorders that share lots of characteristics together, such as difficulties in social and behavioral skills [33]. In order to prevent the misdiagnosis and mistreatment among patients with ADHD and ASD and due to the fact that these two disorders have in common several clinical symptoms, it is important to better understand the differences and similarities between them and study each one separately [34]. 

**Executive function (EF)** is a severe term used to explain the maintenance of effective goal-directed behaviors such as cognitive, emotional, and physical self-control, all referred to as **mental control processes**. Working memory, planning, cognitive flexibility, response inhibition, and fluency are all functions of the EF, and play a crucial role in performing complex cognitive processes. It seems natural therefore to consider these dimensions of EF as related to the cognitive loads induced by the Aristotle’s syllogisms that involve or ‘activate’ some of the above EF. As we describe below, EF is measured by specific multidimensional psychometric instruments that all include the emotional control construct, used also in the current work. Since the main aim of this paper is to detect the shared information (correlation-association) between brain activity in various regions and behavioral data, the knowledge of the brain regions that are strongly ‘linked’ to EF is of high importance in our work in seeking similar strong link between Aristotle’s reasoning process and brain regions. 

To this end, our literature review shows that neural networks in **basal ganglia**, **thalamus,** and **prefrontal cortex** as well as prefrontal areas of the **frontal lobe**, are all important regions helping EFs perform cognitive processes [35]. Plenty of research shows that basic brain regions that are responsible for EF are those that are affected by ADHD and ASD [36,37], a fact that is supported by the deficits in EF shown in patients with neurodevelopmental disorders such as ASD and ADHD [38,39]. Symptoms of those deficits recorded in ADHD referring to response inhibition and working memory deficit [40] while in ASD referring to primary deficit in flexibility and planning [41].

For a better understanding of the distinct domain of EF referring to deficits in ADHD and ASD, there are many studies using EF tests such as the Tower Hanoi, the Wisconsin Card Sort Test (WCST), and the Stroop color-word test, which only analyzes specific domain of EF such as the flexibility, planning, set-shifting, working memory and inhibition, and using diversified subjects (age group, high or low functioned ASD and ADHD). All the above, though, showing inconsistent results due to the above limitations. In order to better examine and evaluate the exact domains of EF that are specifically impaired in each disorder, it is necessary to analyze each disorder alone, in a more comprehensive way.

A psychometric instrument that assesses the EF or equivalently the executive dysfunction is the **Behavior Rating Inventory of Executive Function (BRIEF**), which uses parents’ ratings of their children’s performance of daily executive tasks [42]. **Emotional control,** is one of the 19 cognitive dimensions of BRIEF used in the work of [43], an application of PLSC to explore the relationship between cognitive ability patterns and the differences in local brain anatomy measured by MRI. In the present paper, the emotional control dimension is also included in the behavior matrix, but taken from another instrument, the **SAM (Self-Assessment Manikin),** [44] (see Section 2.4.2 and in Supplementary Materials). The reason for selecting the emotional control in the behavioral data matrix is its high correlation with five of the 19 most important constructs in testing the overall cognitive ability of an individual, namely the monitor (0.59), organize material (0.35), plan/organize (0.51), working memory (0.45), and initiate (0.55), with numbers indicating the correlation [43]. 

### 1.3. Complexity and MSE in Normal and ‘Pathological’ Conditions

A novel approach of analysis and description towards investigating normal or typical and pathological states, degenerative or developmental, is the Physiological Complexity, a term that combines physiology with complexity, the later developed and used extensively in the fields of physics and mathematics. In complexity perspective, seemingly irregular dynamic evolution of physiological signals may contain a significant amount of nonrandom (of ‘nonlinear deterministic’ or stochastic type) fluctuations over multiple time scales, ‘hidden’ in the signals and not easily or never revealed by using ‘typical’ or linear tools of analysis [45,46]. Adopting the complexity approach, Costa et al. [47,48], introduced the entropy analysis in biological and physiological signals or time series, more specifically the Multiscale entropy (the main tool used in this work), while Fallani et al. [49] used graph theory, a nonlinear approach, to study brain functional networks. 

To investigate the differences in complexity of EEGs for the participated groups of subjects, the multiscale entropy (MSE) is adopted in this study. Entropy is a measure to quantify the complexity or order of a system. Systems exhibiting periodic or regular dynamic behavior are said to have low values of entropy. Irregular or random noise-like dynamics have high values of entropy. Regularity and complexity are not necessarily correlated. For example, white noise (a random process), even though it has a high value of entropy does not have the characteristics of a complex system since it does not possess the structural informational richness over multiple temporal scales that a genuine complex system exhibits. A measure of complexity able to distinguish an EEG signal from a linear or nonlinear stochastic signal, behaving the ‘same way’ (for example in view of the autocorrelation function, etc.) as an EEG one, the MSE developed by Costa et al. [47,48], is applied here and it is a powerful tool in detecting the multiple time-scales present in a physiological signal, as the EEG, using a coarse-graining procedure. This type of procedure suggests that optimally operating biological systems are modulated by multiple mechanisms that interact over multiple temporal scales. Therefore for these (optimally) functioning systems, the MSE is expected to have a high value, sustained for increasingly coarser time-scale. Contrary to this, the signals generated by random processes will have MSE or entropy values that decrease as the timescales increase. This is actually an expected result since the information in random noise remains only on the shortest timescale (information is lost), due to the fact that no new structure in the signal is revealed, as the timescale increase. In fact, the variance of the signal decreases, resulting in a decreasing value of the entropy.

A number of articles published relatively recently, provide evidence that a plethora of pathological processes, like in ASD and in ADHD that are examined in this work, are ‘linked’ to atypical and often, but not always, to the phenomenon of reduced levels of physiological complexity regardless the developmental and clinical situations, that could attribute a different understanding on the changes in complexity encountered in such conditions. In the case of detecting differences in EEG complexity between normal and patients with schizophrenia, Takahashi et al. applied the multiscale entropy method [50].

It seems very reasonable to assume that ASD subjects (exhibiting behaviors reflecting the autism spectrum conditions), may be associated with atypical patterns of brain complexity. This is because the core social and cognitive characteristics of ASD, according to the American Psychiatric Association, [51] (restricted repetitive range of behaviors, interests and activities, impairments in social interactions, and qualitative disturbances in communication), as well as recently recognized characteristics as atypical patterns of sensory and motor functioning and interaction [52], atypical visual perception [53], auditory perception [54], and the reduced adaptability to environmental changes [55], suggest that they are possibly connected with EEG signals of atypical complexity. Brain functioning models aiming to explain the above-mentioned features of ASD, include those suggesting disturbances in the underlying brain complexity, atypical neural connectivity [56], and disrupted temporal integration of information [57]. 

The idea that in autism an atypical functional complexity may exists, is enhanced by the observation that subjects without ASD exhibit improved adaptability to cognitive demands or loads associated with increasing variability (volatility), as it is manifested by greater complexity in scalp EEG [58]. Therefore, it seems natural to adopt a complexity measure in order to ‘measure’ the possible differences in the amplitude and frequency dynamics of EEGs taken from ASD, ADHD adults patients, and a matched typically developing control (normal) group of subjects. A surprising and ‘provocative’ result of this paper, as shown in the next sections, is that the complexity-variability of ASD and ADHD subjects, when they face Aristotle’s types of syllogisms, is higher than that of the control subjects. An explanation is suggested as described in the discussion section.

ADHD and ASD are the most prevalent neurodevelopmental disorders. ADHD is characterized by developmentally inappropriate inattention, impulsiveness, and/or hyperactivity that remain relatively persistent over time and result in impairment across multiple domains of life activities. ASD is characterized by persistent deficits in social interaction and communication as well as restrictive, repetitive patterns of behavior, or interests APA [51]. These disorders in most cases persist into adult life. There is a significant unmet clinical and research need to understand the persistence into adulthood [59,60,61].

In typical (normal) development from childhood to adult, brain activity is associated with increasing MSE values [62]. Patients with schizophrenia show an increase MSE in fronto-central and parietal brain regions [63]. The same researchers have shown that treatment of schizophrenia with antipsychotics is associated with reduced entropy. More relevant to our work is the research by Bosl et al. [64], which shows a decrease in resting state EEG complexity, during several phases of development, for infants at high risk of ASD, in comparison with infants at low risk of ASD. As Green, M.F. [65] has shown for the case of schizophrenia, the functional consequences of neurocognitive deficits in ASD and ADHD conditions are ‘translated’ to reduced complexity of the brain functioning. Sustained cognitive operations such as logical reasoning, thought continuity, and working memory, are known to be affected in ASD and ADHD disorders [66,67]. These kind of operations are related to long-time scales (tens of seconds) and correspond to temporal patterns of neuronal activities resembling random-like processes. ASD and ADHD can be deemed as dynamic processes neuronal activities, evolving in time that have not been addressed adequately, as the literature reveals. These processes are associated with highly volatile neuronal states as abnormal functional connectivity, which allows irregular and random-like jumps or switching between different brain regions or neuronal populations.

### 1.4. Emotional Regulation (ER) or Control in ASD and ADHD

ASD is a neurodevelopmental disorder characterized by social communication impairments and stereotype repetitive behaviors. Emotion Regulation (ER) impairments in ASD are associated with the overall severity of all core features of ASD [68]. 

An etiological connection between impaired ER and co-occurring psychiatric conditions has been suggested. Neural anomalies associated with emotion processing in ASD may mediate or moderate downstream ER impairments [69].

Neuroimaging studies help us understand interacting and overlapping brain regions in order examine directly ER in ASD. Pitskel et al. [70] in an fMRI study with 16 children and adolescents with ASD and 15 matched controls participants assessed their increased or decreased emotional responses to disgusting images. Both groups displayed distinct patterns of increasing versus decreasing their emotions. ASD participants showed no modulation of insula and upregulation of left amygdala as well as decreased functional connectivity between amygdala and orbitofrontal cortex. Richey et al. [71] found that among 15 high functioning adults with ASD compared to 15 controls, during cognitive reappraisal of faces ASD adults registered less changes in dorsolateral prefrontal cortex activation compared to controls. Βοth studies support the notion of decreased dorsolateral prefrontal cortex activation during the cognitive control of emotional responses in ASD.

ADHD is one of the most prevalent disorders in childhood and adolescence as well as a still prevalent disorder during adulthood [72,73]. *Emotional regulation (control) deficits (ERDs)* are associated with functional impairments and are considered a translational factor linked to ADHD [74]. ERD is associated with *affect lability* and *emotional reactivity*. Emotion regulation (ER) refers to all efforts to affect the emotions we have, when they happen to us, and how we experienced and express them [75]. According to the model of emotion control of Gross [76], to gain control of emotions, an individual enters a cyclical process in which he/she identifies the emotion needed to the regulated (identification), selects (selection) and executes an ER strategy (implementation). Deficits in *inhibition*, *working memory,* and *executive functions* are neurocognitive correlates of ER in ADHD. 

Regarding ADHD and ER in adults, the study of Marx and colleagues [77] is the first one in which they compare ADHD participants and control subjects on an emotional working memory task: ADHD subjects showed both a general working memory deficit and enhanced distractibility, i.e., lower performance accuracy. In the work of Christiansen and colleagues [78], a strong piece of evidence showed the ERDs are a core component in adult ADHD.

The neurophysiological relation of ER and ADHD is linked to the differentiation of *emotion generation* and *emotion regulation*. The first concept, a bottom up process, is related to the *amygdala, ventral striatum,* and *somatosensory* brain regions, while the second concept (a top-down process) is linked to the *executive neural networks*, with the *prefrontal cortex* to play a central role. Emotional regulation system, is the third part of the top-down regulation that encompass more *posterior parts of the temporal junction (TPJ)* and *medical frontal cortex (MFC)* [79]. 

The other two components are the core system of *self-regulation*, and a *specific action* regulation or *response inhibition* system. The former encompasses the frontal gyrus, the medial frontal cortex (MFC), and the anterior cingulate cortex (ACC). Since we are interested to hear in the emotional control*group interaction effect, we will focus on core systems of ER. The most current evidence suggests that *when cognitive control is absent in emotionally stimulating situations, then this may lead to increased distraction and in consequence in impaired ER in ADHD subjects.* Evidence also is provided in the work of Hajcak and colleagues [80], where event-related potential (ERP) of EEGs is linked to emotion and ER. More specifically, *adult patients with ADHD showed increased late frontal positive potential, extended also to centro-parietal brain locations.*

As far as the psychophysiological connection of ER and ADHD, only a few studies on adults exist, [81,82]. The former work concerns with a study on stress reactivity, a standardized cognitive-psychosocial stressor, stimulating a situation in which a subject is interviewed on a job placement, in conjunction with a mental arithmetic, a *demanded cognitive task exerting “load”* (thus stress) on the subject (this situation is very close to the *valid–invalid tasks* encountered in this work). The main finding in their work was that *subjective stress levels were higher in patients with ADHD compared to healthy controls*. In the second work, the study concerns a frustrating driving simulation, in which *participants with high ADHD symptoms reported more anger and frustration*. In general, evidence for pathophysiologic differences in ADHD adults is weak and sparse, making interpretations hard. 

In Posner et al. [83], children of age 7–12 years with ADHD, (ADHD-Combined, ADHD-Inattentive), ODD, oppositional defiant disorder, Social Anxiety Disorder, and controls were examined in order study their *emotional lability*, including its relation with *handedness*, using the Edinburgh Handedness Inventory tool. Children with ADHD had reduced connectivity in two neural circuits: one underlying *executive attention* and the other *emotional regulation*. They found also that reduced connectivity in the EA circuit correlated with executive attention deficits, but not with emotional lability, while on the other hand, reduced connectivity in the ER circuit correlated with emotional lability but not with executive attention deficits. Compared with healthy (control) subjects, ADHD children exhibit reduced connection strength between the DLPFC and multiple components of the EA network. In addition, they provide evidence that there is a strong relation between EA and ER, more specifically the strength of connection between the *DLPFC and dorsal caudate* strongly predicted *executive attention deficits: weaker connections were associated with more perseverative errors*. An important result that is consistent with a central function of the EA network—to adaptively respond to changing environmental ‘loads’ by filtering out (inhibiting) unrelated—unwanted responses, so facilitating the wanted ones. Also, children with ADHD have reduced connectivity between the VS and the orbitofrontal cortex. Actually, they found a double dissociation between these two neural circuits and their behavioral correlates. Specifically, *reduced connectivity* in the *executive attention circuit correlated with executive attention deficits*, but *not* with *emotional lability*. *Reduced connectivity in the emotional regulation circuit correlated with emotional lability, but not with executive attention deficits. More importantly, reduced connectivity between the orbitofrontal cortex and VS may underlie the limitations on the ability of children with ADHD to regulate emotions. This is because reduced strength of connection between the orbitofrontal cortex and VS diminishes the regulatory control that the orbitofrontal cortex has over the VS and this result in reinforced emotional lability.*

The anterior cingulate Cortex (ACC) is specialized in the regulation of autonomic structures during emotional arousal. It also ‘inspects’ if it is necessary to increase cognitive control and sends this information to the DLPFC [84]. The ventral part of the ACC (vACC) has numerous connections with the amygdala and is sensitive to emotional stimuli, while the dorsal ACC (dACC), which controls the activation of the vACC, is more related to cognitive and regulatory processes [85].

Based on emotive EEG montage (see Section 2.3), electrodes corresponding to our regions of interest, based on the statistically significant results of behavioral analysis (emotion control measure), were selected as seed-regions in the seed-PLSC method to create a ‘primitive’ functional connectivity network. The seeds are as follows: left DLPFC (LDLPFC: F3), right DLPFC (RDLPFC: F4. We examine the information flow (i.e., activation) from the seed-regions to anterior cingulate cortex, ACC (Fz), left temporal area (LTmp: T7, TP7), and right temporal area (RTmp: T8, TP8), within the framework of the Default Mode Network, DMN, proposed in the literature and suitable for the purposes of the current study.

According to the dual pathway model [86], the principal affected neural circuit in ADHD subjects underlies executive attention, EA, which is subserved by distributed cortical and subcortical networks, including the *dorsolateral prefrontal cortex (DLPFC)*, the *dorsal anterior cingulate cortex (dACC), the supermarginal gyrus, the dorsal caudate nucleus, and the frontal eye fields.* However, *Emotional Regulation*, ER, has been proposed also as one of the primary deficits in ADHD [87]. ER system is subserved by frontolimbic neural networks, with components as the *subgenual* and *orbitofrontal cortices*, *amygdala*, *hippocampus,* and *ventral striatum (VS)*. All the above components involved in ER have ‘functional reflections’ on the scalp brain regions on which the EEG electrodes are located. In this study, we will concentrate on the *orbitofrontal cortices* (Left DLPFC: F3, right DLPFC: F4).

## 2. Materials and Methods

### 2.1. The Workflow

**Figure 1:**
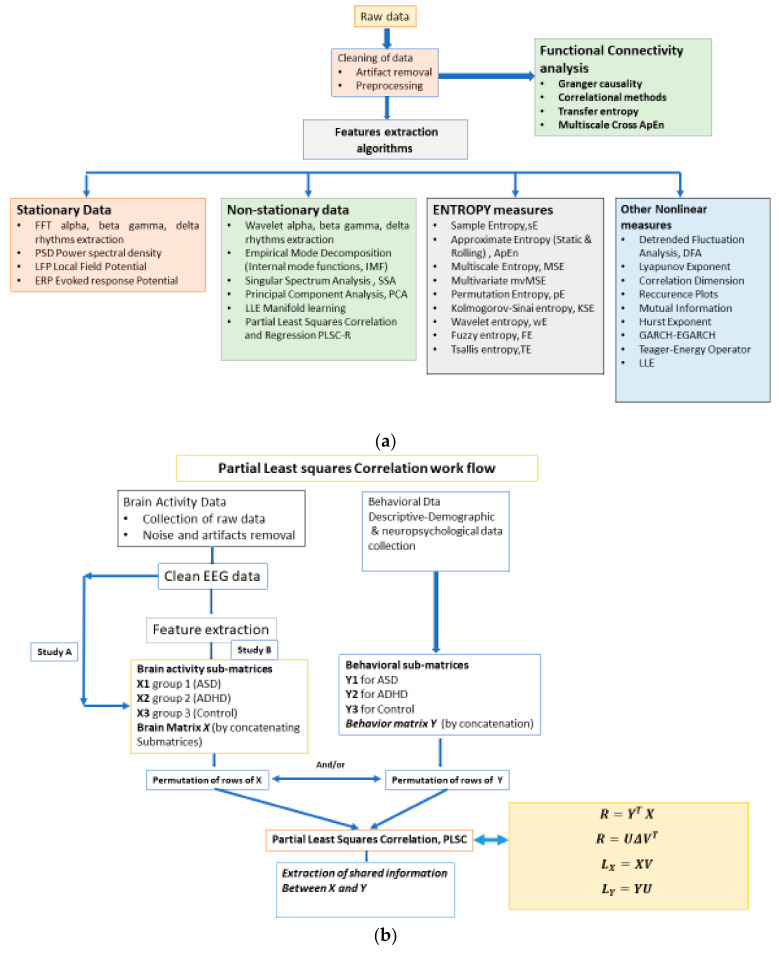
(**a**) An indicative workflow. Our approach (path) is from raw data to preprocessing, Multi Scale Entropy (MSE) in combination with Partial Least Square Correlation, applied on CBD (Cognitive-Behavioral-Demographic) data. (**b**) A work flow of PLSC for analyzing brain and behavioral data in the current study. Entropy values (MSE) were extracted from EEGs and used as input to PLSC (Study B).

For the purpose and the objectives of the present work, there is an extensive spectrum of tools of analysis that could be used. Figure 1a presents an indicative workflow that one can follow to study how the dynamic behavior of recorded EEGs of subjects, in different groups (control and ‘pathologic’) is affected, when the subjects are ‘tested’ in valid and valid Aristotelian syllogisms. In this paper we follow the path from raw data analysis and preparation to the ‘simultaneous’ usage of two different but nonlinear approaches: the Partial Least Squares Correlation, PLSC analysis and MSE analysis, that provide an efficient and effective tool towards detecting the complexities and their changes, at various time scales. The work flow of the application of PLSC in the present paper is provided in Figure 1b in which two possible studies are shown, study A and B. In A, the PLSC is applied on the clean, ‘original’ EEG signals in each channel, while in study B the method is used on the MSE feature values, extracted from the EEGs. In the second study that is conducted in this paper, PLSC is more efficient (its reliability is enhanced) when applied on MSE values.

In this paper, we perform a *behavior PLSC* to analyze the relationship between the *behavioral characteristics* of groups ASD, ADHD, and control, and their *functional brain activity* (other variations are *PLSC task, PLSC seed, or multi-table*) [88]. The process is depicted in Figure 1a, which illustrates the construction of matrices *X, Y,* and extracted correlation matrix *R*. The observations (mean MSE per subject) are arranged according to 3 conditions (ASD, ADHD, and control). 

### 2.2. Participants

The distribution of 63 participants in the study is as follows: 21 ASD, M = 14 (66.6%), F = 7 (33.3%) with group average age of 31.2 years (SD = 10.52 y), 21 ADHD, M = 14 (70%), F = 6 (30%) with group average age of 28.65 years (SD = 8.75 y), and 21 normal, M = 12 (57,1%), F = 9 (42.86%), group age average 28.05 years (SD = 6.152 y).

The descriptive statistics of the demographic data of the participants collected by questionnaires, including education level, occupation, smoking, right- or left-handed, health issues, and treatment, are provided in Supplementary Materials. The data are taken from a study that is part of a larger research project on de novo diagnosed adults with ADHD and ASD [89]. The multi-disciplinary team that carries out all assessments consists of psychiatrists who have extended experience in the diagnosis and treatment of neurodevelopmental disorders in adults and are trained in ADOS [90,91], ADI-R [91,92], and DIVA [63,64]; and clinical psychologists. In order to be included in the study, subjects had to be adults with normal intelligence and fluent phrase speech and to be assessed for the first time in their life for a possible ADHD and/or ASD diagnosis. Exclusion criteria were a previous ADHD and/or ASD diagnosis, the presence of acute psychopathology requiring urgent psychiatric treatment, current substance abuse disorder, IQ < 70 according to WAIS and a known genetic cause. Diagnosis regarding the presence of ADHD and/or ASD is given during a consensus meeting of the multidisciplinary team and is based on DSM-5 criteria while taking into consideration all available information. Written consent was obtained from all participants and the study was approved by the Ethics Committee of our University Mental Health Research Institute (UMHRI).

### 2.3. EEG Recordings

**Figure 2:**
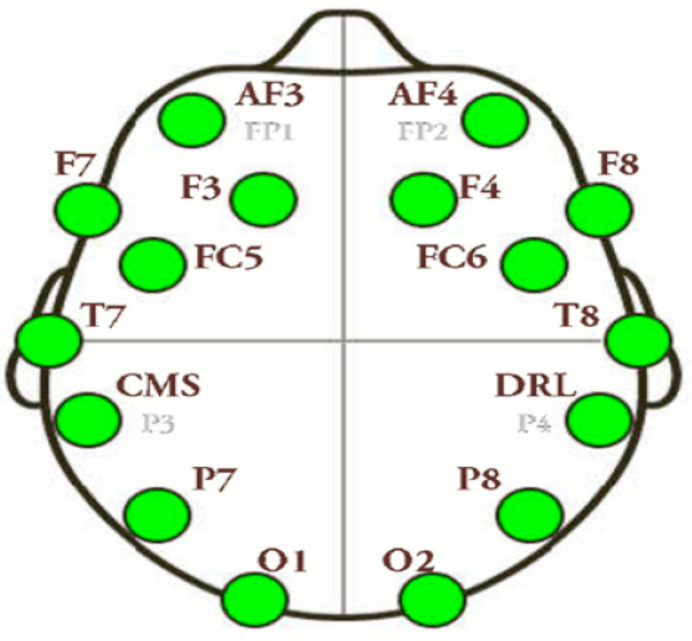
EEG recording electrode scalp locations in emotive EPOC system.

In this work, for EEG signals recording, the Emotive EPOC system was used, consisting of 14 channels (plus CMS/DRL references), following the 10–20 international system of locations. The channel names are AF3, AF4, F3, F4, F7, F8, FC5, FC6, P7, P8, T7, T8, O1, and O2. Sampling was sequential, with single ADC, and rate 128 Hz (2048 Hz interval). All electrodes were placed over subject scalp. Band pass was filtered between 0.05 Hz-35Hz, in order to avoid interference due to power supply netwok’s signal (50Hz). Ground and reference electrode were placed on left and right ear lobes. The electrode impedance below 5 KΩ was kept. The scalp locations for the Emotion EPOC system are shown in Figure 2 and Table 1.

In the case of our study, the Emotiv EPOC device has provided several important benefits (i) compared with more expensive multichannel equipment: the setting up time of the Emotiv EPOC system is significantly shorter than that of an expensive EEG system, (ii) additionally, recent research assessing the reliability of the EMOTIV Epoc EEG device provides converging evidence indicating their capacity to measure consistently EEG signals [93,94]. After cap fitting, good conductivity was confirmed with Emotiv software through wet saline electrodes [95].

### 2.4. Tasks Description. The Aristotle Experiment

Using EEG signals, we aim to isolate the particular brain regions involved in Aristotelian types of reasoning or syllogisms, and to differentiate their engagement during the different types of syllogism: valid, invalid, illusions, and paradox. In addition, we will try to answer how this differentiation is accounted for in the case of three groups of subjects (control, ASD, and ADHD).

Care has been taken so the sentences or prepositions in the above types of syllogisms were presented visually (on a computer screen), in order to dissociate brain regions related to syllogism processing from those related to sensory process and low-level reasoning. This is a crucial stage in the process of analysis since the isolation of substrates associated with this high-level syllogisms (activating cross-modal cognitive systems), is not an easy task. In the experiment, the syllogism statements consisted of categorical type and the participants were informed to reach to a conclusion from the premises provided by the instructor, and indicate whether the given instruction was ‘True’ or ‘False’ (see below).

Aristotle’s experiment is based on the study of reasoning or syllogism process, based on logical rules and concerns the way we reach to a conclusion (reasoning process) and on the way we take decisions. The process starts with assumptions (hypothesis) that ideally lead to a valid conclusion. The most known theoretical model ever proposed so far is that of dual process theory. Type or system I of the process is considered old, fast, and automatic, while type or system II is newer, slower and allows reasoning based on logical rules. In type I, we are aware only of the result (conclusion), while in type II, we are aware of both the way and the result. Usually, there is interaction between the two types. The question that arises often is whether personality or psychopathology enter the reasoning process. The Aristotle experiment is a process consisting of four stages. Valid and invalid syllogisms are used together with paradoxes and illusions (the last two are not considered in this study). 

#### 2.4.1. Stimuli and Procedures Stimuli

The experiment was designed to contrast two mental functions (corresponding, as it is assumed, to two different cognitive loads): processing of syllogisms characterized as “valid” vs. processing of “invalid” reasoning. This was done by exposure of the participants to two arrays of statements, one containing 39 valid syllogisms, the other 39 invalid. The order of presenting the valid and invalid sections was *counterbalanced* across participants, in order to avoid *confounding effects*. Two indicative examples follow: the *valid* array included statements of the following type: “All men are animals. All animals are mortal. Hence, all men are mortal.” [96]. Common mistakes affecting the validity of an argument are called *“denying the antecedent” and “affirming the consequent.*” Both are forms of invalid syllogism that occur when the As and Bs are swapped. The example below (see Table 2) is a syllogism about George and his capacity as a gamer. The *invalid* array consisted of statements of the type shown in table (Aristotle, Prior Analytics, translated by [23]).

#### 2.4.2. Behavioral Data and Procedure

The subjects under test were seated comfortably 1 m away from a computer screen in an electromagnetically shielded room. Instructions to read carefully each statement were presented to the subjects, before the start of the experiment, so they can be familiar with the process and its requirements. Then, after two presentations of a warning sound, syllogisms or arguments started to appear on the screen, in sets of 39, and every one of them was accompanied by a question, ‘right’ or ‘wrong’. The time duration of each slide is proportional to the number of letters in the syllogisms and then the slide disappears. Immediately after, the next stimulus appeared, which may be a right or wrong answer. The subject is called to give answer. Answers to each one of the ‘valid’ syllogisms are considered right if subject considers them as right, while answers to ‘invalid’ syllogisms are considered right if subject considers them wrong. Each subject’s answer is accompanied by his *percent (%) of certainty or confidence level* given, which reflects his certainty in the answer he provides (100% absolutely certain and 0% not at all certain).

In parallel with the above process, a recording of the emotional condition of each of the tested subject is taking place (its *intensity*, *control,* and *mood* of the emotions he feels), according to the *Self-Assessment Manikin (SAM)* instrument that directly assess the *pleasure (valence or strength), arousal, and dominance (regulation or control)* dimensions-constructs of emotions, in response to an object or event [48,97]. The SAM instrument for assessing the dimensions of emotion state is shown in Figure S1 in Supplementary Materials. The numbers indicate the rate scale, with 2, 4, 6, 8 the rates between two pictures, allowing a 9 point scare rating of a participant state of emotion. EEG signals were simultaneously, with the above procedure, recorded, using a wireless system of 14 electrodes (EMOTIV PRO, see Section 2.3).

SAM is easily administered, a non-verbal instrument for assessing fast the pleasure, arousal and dominance (control) association with a person’s emotional reaction to an event. We used SAM in this report to assess how ASD, ADHD, and control participants, reacted emotionally to the valid and invalid Aristotle’s cognitive tasks. According to differences obtained in judgments of control, SAM is possibly more accurate in tracking the participants—rather than stimulus—feeling of control. Due to these characteristics, SAM is a useful tool when “quantifying” the subjective experience of emotion related to processing most stimuli. Also, SAM can be employed with a variety of subject populations (adults, children, subjects having language disorder as well as all clinical syndromes, as ASD and ADHD in our case). 

SAM was applied in the work of [98], for emotion recognition, using empirical mode decomposition (EMD) on DEAP EEG data base, a multimodal data set [99] consisting of EEG recordings while watching the selected video clips to analyze human affective states. The extracted intrinsic mode functions IMFs from EMD were used as features of *valence* and *arousal* dimensions of participants that are used in plotting a 2-dimensional presentation of valence vs. arousal of emotional state, based on the *Circumplex Model of Affect* of Russell [100] and its new version of Posner [101]. In Section 3.1.1, we show the 2-D plot of the variation of the arousal and valence dimensions scores of the three groups. 

In comparison with the more children-oriented and modern instrument of *BRIEF (Behavior Rating Inventory of Executive Function)*, SAM is a pictorial technique that assess directly three measures of pleasure, arousal, and control, related to subject’s affective reaction to a wide spectrum of stimuli. BRIEF measures 8 dimension of behavior, with *emotional control*, which according to Ziegler [47] is strongly correlated with other crucial dimensions of executive function, as working memory (0.45), which is very related and important variable in cognitive processing as our valid–invalid tasks. As we describe in Results, emotional control was found to be statistically significant when taken together with handedness (see Section 3). 

In this work, we extracted MSE values from the EEGs and use them as brain data, while the behavioral data are as shown in Table 3. The two data sets are used as input to the PLSC method in order to distinguish how the shared information between the two sets depends on the group of participants. As already mentioned, it is preferable working with MSE feature values, because they incorporate more information due to nonlinear interaction of the brain regions. Therefore, it is expected that the PLSC works better when applied on MSE values instead of EEGs.

### 2.5. The Partial Least Square Correlation (PLSC) Method

**Figure 3:**
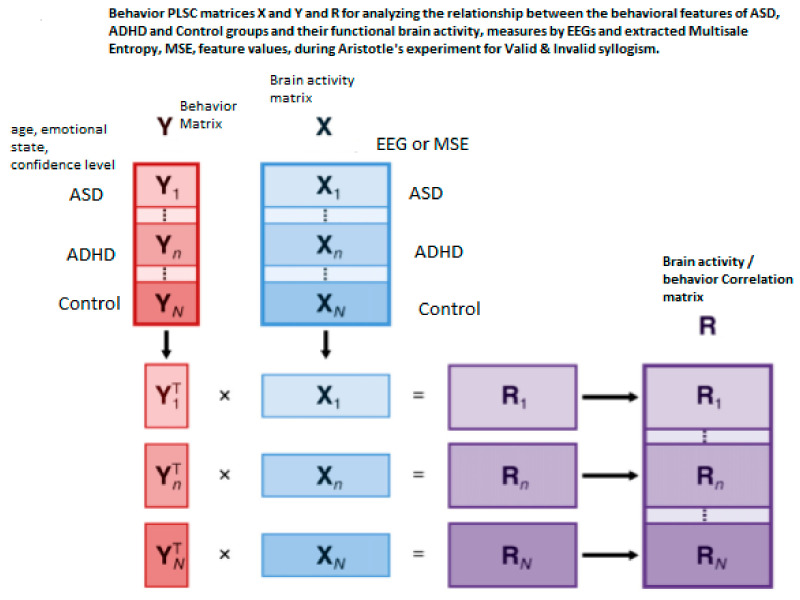
Construction of matrices X, Y, and extracted correlation matrix R. The observations (mean of EEG or MSE per subject) are arranged according to 3 conditions (ASD, ADHD, and control). *X, Y* are normalized within condition. The matrix of correlations *R_n_* between each condition-wised sub-matrix (*X_n_, Y_n_*) are stacked one below other to form the total-combined correlation matrix R, which finally is decomposed by singular value decomposition, SVD.

PLSC, originally developed for econometrics [102], is a member of the PLS, a versatile family of multivariate methods. It is focused on analyzing two tables (matrices) of data measured on the same observations. PLS has been applied to *regression* (PLSR) [103,104,105,106], *correlation* [1,88], *path-modeling* (PLSPM) (Esposito et al., 2010) [107], genetics and genomics [108], in *combined studies of genetics and brain data* [109]. PLS correspondence analysis (PLSCA), is tailored for specific data and design issues encountered within the context of psychological, cognitive, and neurological fields. It is particularly versatile in analyzing simultaneously data tables of *a mixture of continuous and categorical data* [110]. PLSC is applied to *neuroimaging* [1,88] and recently it has been proved to be the best covariance method in detecting generic links in high dimensional neuroimaging data [111]. Chen et al. [112] applied PLSR in estimating an individual’s cognitive-behavioral-demographic (CBD) variables based on MRI’s resting state functional connections (RSFC). More specifically, RSFCs has been reported to be effective features in estimating a plethora of CBD variables, as *temperaments traits* [113], *intelligence quotient* [114], *visual-verbal memory* [115], *sustained attention* [116], *age* [117], and *gender* [118]. PLS analysis is applied on *electroencephalography (EEG)* data in the work of Aylin [119], in identifying stimulation dependent changes in EEG activity. More specifically, the aim of the study was to determine if three different stimulations differ significantly and which part of the brain in linked strongly with this differentiation. The pictures of an anonymous elderly lady and of a known face and the light were the complex and simple stimulations. The present study further extend the above work by including a number of behavioral data and analyzing their correlation with EEG data, i.e., combine a brain activity matrix (containing the average MSE values per subject) with a Behavioral Matrix (containing behavioral—demographic data) (see section Materials and Methods). *X* and *Y* are the brain and behavior data, respectively. *X* contains MSE values at each channel and *Y* the SAM measures (age, level of confidence in answering questions, and emotional state). Thus, in our case (see also Figure 3): 

*X* matrix has 21 rows (one per subject) for ASD, 21 for ADHD, and 21 for control, and 14 columns corresponding to channels. *Y* has 21 rows for ASD, 21 for ADHD and 21 for control, and 3 columns corresponding to 3 behavioral measures. 

*X and Y* are normalized within condition. The matrix of correlations *R_n_* between each condition-wised sub-matrix (*X_n_, Y_n_*) is stacked one below the other to form the total-combined correlation matrix R, which finally is decomposed by Singular Value Decomposition, SVD. The behavior matrix *Y* contains the same variables in both studies, one demographic *(age*) and two neuropsychological (*emotional state* and *level of confidence* of participants. The rows of *Y* are the same participants as in matrix *X* and its columns are the participants’ age, and values of emotional state and confidence level. Both *X* and *Y* are centered (the mean is subtracted) and normalized within each group n (*X_n_* and *Y_n_* are centered and normalized independently, so the sum of squares of a column in say ASD is equal to 1). The matrix of correlations for each group n is computed as $R_{behavior}=Y_{behavior,n\;}^{T}X_{n}$. This matrix is the input to the SVD. $R_{behavior}$ contains the correlation of each of the j = 14 EEG or MSE values (at each channel) in *X* with each of the k = 3 behavioral measures (age, emotional state, confidence) in *Y* within each of the N = 3 conditions or ASD, ADHD, and control groups. Thus, *R* will contain N × K rows and J columns, as shown below:

$$R_{behavior}=\left[\begin{array}{c} R_{behavior,1}\\ R_{behavior,2}\\ R_{behavior,3} \end{array}\right]$$

where each of the N × K rows provides the kth behavioral measure for the nth group and J columns reflect the EEG activity or MSE feature values. The SVD of $R_{behavior}$ is computed as

$$R_{behavior}=U\Delta V^{T}$$

where **U** is the N × K row and L column matrix of the saliences for the behavioral measures, with L the rank of $R_{behavior}$, and Δ the diagonal matrix of singular values, and finally, **V** the J × L matrix of the saliences of the brain activity.

The **U** matrix of behavioral saliences indicate EEG- (or MSE-) dependent differences in the brain–behavior correlation, or equivalently the interaction of the experimental conditions with the behavioral measures. Brain saliences **V** reflect EEG-dependence differences in the brain behavior correlation.

The correlation of the behavioral variables with brain scores provide a pattern of scores similar to **U**, reflecting the differences in the behavior. According to [1], in order to assess the reliability of the brain–behavior association, confidence intervals must be estimated.

In order to examine how the experimental conditions (ASD, ADHD, and control groups) interact with each behavioral variable (age, emotional state, and confidence), a plot of each column of **U** against each behavioral variable is needed (see Section 3, results). *In order to express the saliences in terms of brain activity and behavior, the original matrices **X** and **Y** are projected onto their respective saliences, and this projection creates the latent variables that are linear combinations of **X** and **Y**. PLSC searches for latent variables that express the largest amount of shared information of **X** and **Y** (i.e., latent variables with maximal covariance).*

The latent variables ***Lx*** of ***X*** and ***L_Y_*** of ***Y*** are computed from the aforementioned respective saliences. To recall, ***L_x_*** is called *Brain Scores* and ***L_Y_*** *behavior sores*, and both matrices have I rows and **L** columns. The *Brain Scores* are given by ***L_x_ = XV***. The latent variables for behavior per group is given by the product of the rows of ***Y*** and **U** corresponding to each group. The combined behavior scores are formed by concatenating the group-wise behavior scores, so the latent variable for the *n*-th group is given as follows:

$$L_{Y,n}=Y_{n}U_{n}$$

Thus, the **L_Y_** matrix is finally formed as $L_{Y}=\left(\begin{array}{c} L_{Y,1}\\ L_{Y,2}\\ L_{Y,3} \end{array}\right)$.

The illustration of ***L_x_*** and ***L_Y_*** is not done with the typical way. We can use however PCA style plots, as shown in the results section, which can help us in observing how the first and second brain scores separates the participants in ASD, ADHD, and control groups from each other, and similarly how the first and second behavior scores does the same thing. Permutation tests are also performed, as described below, to enhance the reliability of the results and extend them to the population. Figure 3, the ‘construction’ of all the matrices and sub-matrices involved in the PLSC application on the data of the current study.

The PLSC is divided in four versions: (a) behavior PLSC, (b) task PLSC, (c) seed PLSC for functional connectivity analysis, and (d) multi-table or multi-block PLSC [1]. The difference between the methods rests in the structure of the matrix *Y* (corresponding to behavioral or design variables), introduced below. In behavior PLSC, which is the main method adopted in the present study, *Y* is a matrix of behavioral variables, a matrix of contrasts or design variables in task PLSC, a matrix of voxel or EEG activity in seed-PLSC. The last technique is also applied to our study, on MSE values instead of EEGs, in order to analyze the *functional connectivity* formed by the interactions of the most activated brain regions, as a result of the cognitive loads induced by valid–invalid reasoning. The seed-PLSC is described in Section 2.5.2.

**Figure 4:**
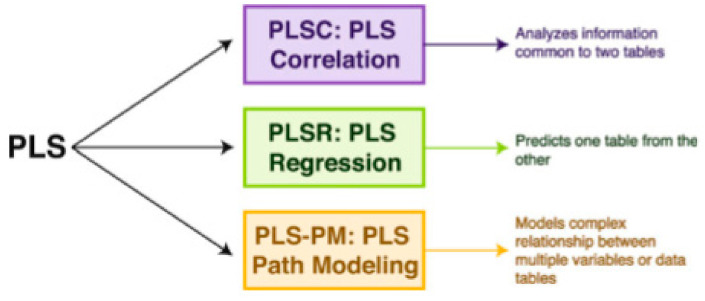
PLS methods with their main goals. PLS Correlation is the method adopted in this work.

PLSC is a method used to find the shared information between two data matrices (tables) that collects measurements on common sets of variables. Partial least square, PLS, derives latent variables that are (optimal) linear combinations of the variables in a data matrix. PLSC is based on singular value decomposition (SVD), the main analytical tool in decomposing a matrix [120,121]. PLS methods with their main goals are depicted in Figure 4.

Let two data matrices of sizes I × J and I × K, symbolized (respectively) by *X* and *Y*, which contained measures of the same I observations (rows), described by J and K (respectively) variables (columns). Matrix *X* corresponds to brain activity, while the matrix *Y* corresponds to behavioral or design variables)

Let the normalized and centered versions of *X* and *Y* denoted by *Z_X_* and *Z_Y_*, respectively. The following relation describes the common information in the two matrices

$$R=X^{T}Y\;and\;Z_{R}=Z_{X}^{T}Z_{Y}$$

where *R* is a J × K cross-product matrix, the correlation matrix (*Z_R_*_,_ in centered and normalized form). *Z_R_* is the variable that is used further in this analysis, and its SVD decomposition is written

$$Z_{R}=U\Delta V^{T}$$

If L is the rank of *Z_R_*, then U is a J x L orthonormal matrix of left *singular vectors*, V is the K × L orthonormal matrix of right singular vectors, and Δ is L × L diagonal matrix (the off-diagonal elements of Δ are all 0). The elements of diag{Δ} are the singular values (with ordering from *larger to smallest). The eigenvalues (the squared singular values) describe the variance of the* data extracted by the components. The matrices of singular vectors *U* and *V* in the PLSC ‘vocabulary’ are called *saliences* [1,92]. To correspond these variable with those in PCA (Principal Component Analysis), the matrices *U*Δ and *V*Δ are ‘equivalent’ to principal (factor) scores [105]. The original variables *Z_X_* and *Z_Y_*, in PLSC, are combined linearly to create pairs of *latent variables* (each pair has one latent variable from *Z_X_* and one from *Z_Y._* The latent variables of *Z_X_* and *Z_Y_* are given by

L_X_ = Z_X_U and L_Y_ = Z_Y_V

Matrix L_X_ is called brain scores, and matrix L_Y_ is called behavior or design scores.

In PLSC analysis, two vectors of coefficients are sought, namely u and v, which define the linear combinations of the columns of Z_X_ and Z_Y_, respectively, called latent variables, denoted l_X_ and l_Y,_ computed as $l_{X}=Z_{X}u$ and $l_{Y}=Z_{Y}v$, respectively, and have maximal covariance, expressed as follows:

$$\delta =arg\;max\left(l_{X}^{T}L_{Y}\right)=arg\;max\;cov\left(l_{X},l_{Y}\right)$$

with the constraints that in the linear transformations for Z_X_ and Z_Y_, the coefficients have unit norm

$$u_{l}^{T}u_{l}=1=v_{l}^{T}v_{l}$$

After the extraction of the first pairs of the latent variable, additional pairs are also extracted, on the condition that unpaired sets of latent variables are orthogonal. Using SVD (2), the matrices L_X_ and L_Y_ that contain the coefficients of the linear transformations, which are given as follows:

$$L_{X}^{T}L_{Y}=U^{T}Z_{X}^{T}Z_{Y}V=U^{T}Z_{R}V=U^{T}U\Delta V^{T}V=\Delta$$

In case l = 1, the covariance between L_X_ and L_Y_ attains the maximum possible value, and when l = 2, the covariance between L_X_ and L_Y_ has also the maximum possible value, but now under the constraints that the second pair of the latent variables are orthogonal. The relationships between the jth column of *X* and the kth column of *Y* is measured by the scalar product of these two columns. Due to centering of these matrices, as mentioned above, the scalar product gives the covariance between the two columns. The product reflects the correlation between them, when in addition to the centering, the columns are also normalized (transformed to z-scores). Since correlation and covariance do not depend on the order of the variables, they are not directional, so the roles of *X* and *Y* are symmetric and the analysis concentrates on shared information [88]. Once more, PLSC computes latent variables with maximal covariance. 

In order to decide which latent variables to keep, the computational approach of permutation tests is adopted here, to obtain *p*-values, which can then be used to generalize the found latent variables [88]. 

#### 2.5.1. Common Inertia in PLSC and Significance of Inferences in PLSC and Permutation Test

The information common between the two matrices (brain and behavioral) can be directly quantified as the *Inertia* common to these data sets, denoted as *I_total_*

$$I_{total}=\sum_{l}^{L}\Delta_{l}\;$$

where $\Delta_{l}$ denotes the singular values from (3) (i.e., $\Delta_{l}$ is the *l*th diagonal element of **Δ**), and *L* is the number of nonzero singular values of R.

PLSC is a multivariate method and as such an additional inferential step is needed in order to assess if the results can be accepted as reliable or significant. In this work, we assessed the global model (SVD of correlation matrix) via permutation test, a computational cross-validation method [122]. The test provides a nonparametric estimation of the sampling distribution of the parameters computed, allowing a hypothesis testing to be performed. In our case, the rows of *X* were randomly permuted (to obtain a *random effect model*), therefore any relationship between *X* and *Y* (e.g., via *R*) is replaced by a random form. The new matrix *R_perm_*, reflects now only random associations of the original data (due to randomization of rows in *X*), and the SVD analysis in (2) is repeated again, giving new singular values. We note here that SVD on the original *R* corresponds to *fixed effect model*. The overall index of effect $I_{total}$ (i.e., the common inertia) is computed repetitively for a large number of times (in our case 10,000 times). Then, the probability distribution is $I_{total}$. If the common inertia computed for the sample is rare (for example <5%), then this index is statistically significant (the test actually corresponds to an omnibus test that tests the overall effect). 

In this work, we tested if the overall analysis extracted relevant information between *X* and *Y*, generated 10,000 *R* matrices by permuting only the rows of *X,* computed the $I_{total\_perm}$, and compared it with the ‘deterministic’ $I_{total}$. We did the test for both cases, based on the amplitude of EEGs as well as on the MSE feature, for valid and invalid type of syllogism. In the supplementary material (Figure S2a,b), we report the outcomes of the tests. In both cases, the deterministic values of $I_{total}$ were obtained in the distributions of $I_{total\_perm}$ with very small probabilities, *p* < 0.05, so *the results were found to be statistically significant*. 

#### 2.5.2. Brain Functional Connectivity Analysis via Seed-PLSC within the Default Model Network (DMN). ASD and ADHD Abnormalities in DMN’s Connectivity

##### DMN

All the analytical steps that are performed in this work regarding PLS are based on the assumption that *behavior* and *cognition tasks* (valid–invalid syllogisms) are the results of the *integrated activity of brain-dynamic networks*, rather than the actions of isolated (independent) regions that operate independently. Another target of this paper is also the investigation of the changes in the brain specific regions that depend on the ‘dynamics’ of the interaction of EEG (MSE) variability with the three behavioral measures. These changes can be detected via observing the structural changes of a *functional connectivity map* created by using the *seed-PLS approach*, a variant of PLS methods, *within the Default Model Network (DNM).* The involvement of DMN as a ‘*reference network’* within which the seed-PLS created functional map is analyzed, is explained below. In the literature, DMN has been extensively investigated. Especially the combination of DMN and the power spectrum analysis (α, β, γ, δ, and θ rhythms) of EEG has been used by Chen A.C.N. et al. [123] to detect significant differences in DMN’s network in healthy subjects in eyes-closed and eyes-open conditions. DMN is the one of the most important *resting state networks*, RSN [124], and is identifiable in three regions: *(a) precuneus/posterior cingulate (PPC), (b) lateral parietal cortex (LPC), and (c) mesial or medial prefrontal cortex, (mPFC).* mPFC together with anterior cingulate Cortex (ACC) are embedded in an organized network for initiation of functional ‘self’. The dorsal mPFC increases in internal feeling judgment and the ventral mPFC reduces in external cognitive judgment.

Typically, DMN shows higher neuronal activations during resting state than the induced neuronal activation during task performance (as during valid–invalid reasoning, in our case), which require attention such as working memory [125,126]. However, a plethora of other studies have also shown that DMN exhibits increased activation when performing particular tasks, and in addition, DMN instead of simply disengaging in active tasks, may facilitate or monitor performance of that task [127]. 

Therefore, and in general, the detection of statistically significant correlation between DMN connections and behavior, indicates that the monitoring of the functional changes in brain connectivity is proved to be extremely useful in analyzing the behavioral differences of the participants. Thus in our case, if we find that the functional connectivity between regions of DMN, which is considered as *a ‘reference’ network*, or as a biomarker, is significantly changed during the valid–invalid cognitive loading, this will guide us to distinguish the response of ASD, ADHD in comparison to control subject. This approach has very recently been followed by [128] in an fMRI analysis of the alcoholism, using the DMN as a biomarker, i.e., monitoring the effective connectivity of DMN. More importantly, recent studies provide evidence that *DMN’s regions are involved in cognitive, behavioral, and motor functions*, in addition to their interactions with other cortical-subcortical brain regions. This explain why we have decided in this paper to analyze the seed-PLS generated functional map within the DMN context. According to [128], electrodes Pz, P3, P4, F3, F4, and Fz are those that are related to the three above-mentioned DMN’s regions. From an extensive table they provide in their work, we focused on the functions on which we are interested in our paper (mainly emotional and executive, e.g., working memory), which are related to the DMN’s areas and the corresponding electrodes. Since in our case only the F3 and F4 electrodes are involved, the functions related are: For electrode F3, the medial prefrontal cortex mPFC of DMN is associated with the memory function, the *working memory* [129]*, and inductive reasoning (two functions involved in valid–invalid Aristotle’s reasoning), while for electrode F4 the DMN’s mPFC is involved in the executive control of behavior in the motor function* [130]*, memory retrieval, and sequential learning in memory function* [131]*. The executive control of behavior, planning decision making, working memory, and attention functions are also associated with mPFC of DMN and electrode Fz (absent in our study, but close and between F3 and F4).*

The core regions associated with DMN that are disrupted in ASD patients have been examined in the works of [124], in which they report a number of studies where ASD subjects show significant differences in the DMN core regions (ventral medial PFC, vMPFC, and posterior cingulate cortex, PCC), compared with healthy subjects. Participants with ASD fail to represent mental states of others regarding problems’ solutions (theory-of-mind tasks) [132]. Mundy [80] and other researchers, based on the theory-of-mind and social interactions, support that *MPFC (Medial Prefrontal Cortex)* plays a crucial role in understanding ASD’s deficits. 

A plethora of research efforts have examined whether there are associations between *anatomical*-*morphological differences* in brain structure and ASD. Studies have revealed that there are complex brain changes, appeared at different developmental stages, regarding the cerebellum and amygdala with the latter being *increased in volume* in children with ASD [133,134]. It has to pointed out that amygdala is responsible for the social cognition and has wide projections to *orbital frontal cortex (OFC)* and *ventral medial PFC*, *vMPFC* [135,136,137,138]. 

Other studies have shown differences in the brain volume of children with ASD and more specifically in *dorsal medial PFC (dMPFC),* which reflects greater volume reductions. From the study of Waiter and colleagues [139] using voxel-based morphometry to examine differences in grey matter area among male adolescents with ASD, results showed that a number of regions related to *Default Mode Network (DMN)* a revealed greater increase in grey matter volume compared to the control population. However, this study needs further investigation upon adults group with ASD, in order to examine whether behavioral improvements and developmental stages plays a severe role regarding the DMN [140]. 

DMN differences among adults with ASD and control participants have been examined by Kennedy and colleagues [141] using fMRI, by exposing participants to *passive and demanding active tasks (as in our case the valid–invalid reasoning)*, which revealed that while control participants showed a typical pattern of activity in the default network, *adults with ASD had no such activity at all*. Also, *differences found among these two groups between vMPFC and Posterial Cingulate Cortex PCC* showed that individuals with greater social impairment had the most atypical vMPFC activity levels. A possible explanation for the failure of ASD in adults is due to the modulation of the DMN, as proposed by Iacoboni [142], according to which, this due to the lack of *self-referential processing.* Finally, in the recent study of Cherkassky and colleagues [143], using analysis of *intrinsic functional correlations*, results showed weaker levels in default networks in ASD participants. Also, ASD participants showed differences in the *frontal-parietal network*, a finding that strongly correlates with the control interactions between default networks and brain systems related to external attention. 

In the paper of Anteraper S.A et al. [144], cerebellar abnormalities and especially in the *Dentate nuclei (DNs)* (that are key structures in the anatomical circuits connecting the cerebellum to the extracerebellum) are reported in ASD young adults, in an MRI study, in which a *seed-based resting state functional connectivity analysis* was carried out. They showed that the between-group (ASD and control) DMN connectivity contrast revealed abnormalities in both the cerebellar DMN and right supramarginal gyrus (SMG). Their study is the first one to establish a correlation between ASD symptom severity and DN functional connectivity.

Regarding the ‘dynamic behavior’ of the DMN network in the case of ADHD subjects, an examination of healthy controls verified presence of an antiphasic or negative relationship between activity in *dorsal anterior cingulate cortex, dACC* (centered at x = 8, y = 7, z = 38), and in DMN components [145]. The authors report that group analyses revealed ADHD-related compromises in this relationship, with *decreases in the functional connectivity* between the anterior cingulate cortex ACC, and precuneus/posterior cingulate cortex, prCC and posCC, regions. Secondary analyses revealed an extensive pattern of *ADHD-related decreases* in connectivity between precuneus and other default mode network components, including *ventromedial prefrontal cortex vPFC*. Abnormalities in the DMN in ADHD subjects have been shown in [146,147].

Related to our study of DMN, is its network change with age, one of the three significant ‘behavior’ measures found. *The strength of coherence between nodes of the DMN brain system varies with age* [124,148]. Young adult pattern of DMN may recede as one passes into the sixth decade of life and beyond [149], even in healthy older subjects.

##### Seed-PLS Functional Connectivity and DMN

Based on above, we used the MSE values evaluated at each channel to detect and ‘uncover’ these networks via the *seed-PLS* approach. We must note here that this method does not provide information about the directions of the ‘causal’ effects, but rather provides information about the regions of the brain activated due to the activated seed regions of F3 and F4, since our main target of the present work is to analyze brain dynamics-behavior correlations. For detecting ‘directed’ causal-effects between regions, other tools are more appropriate like Granger Causality, Directed Transfer Function (DTF) and Partial Directed Coherence (PDC). In a future paper we plan to use these methods to examine the full directed functional connectivity maps. 

Two steps are typically followed in ‘building’ the functional connectivity of the brain regions interactions, based on the location of the channels. In step one, a test is carried out for investigating brain activity modulations, which are attributable to a given cognitive task, in our case to the valid–invalid tasks. We are interested in connecting brain dynamics, due to valid–invalid cognitive tasks, with the *arousal and control (regulation) of emotions* measures that the participants have self-reported. This test was replaced in our case by an extensive literature review, described in this section as well as Section 1.4, which refer to the brain regions involved in the regulation of emotions of a subject (*ventral anterior cingulate),* which strongly interact with the *dorsolateral PFC (prefrontal cortex). The review* resulted in considering these regions as the most appropriate seeds, so we choose channels F3 and F4 as the most representative of those regions, given the electrodes’ location in our case. The second step identifies which specific regions out of the other 12 channels are more active during the valid–invalid tasks, and whether they are different in participants of the three different groups. By more active it is meant regions correlated with F3 and F4 ‘seed-*reference regions’,* the right- and left-dorsolateral PFC (r-DLPFC, l-DLPFC). In the seed-PLS technique, the seeds are chosen either by performing a multivariate regression analysis on the MSE values (choosing appropriate dependent and independent variables of interest) or by using another variant of PLS, the task-PLS, which can detect the channels of interest that are strongly activated during the cognitive tasks or based on the theoretical information or on results of other empirical analysis, found in literature review, as in our case. From the literature review, given above, we concentrate on the activities of channels F3 and F4 (corresponding to r-DLPFC and l-DLPFC), which are ‘linked’ to the *emotion arousal and control behavioral* measures, strongly activated during the valid–invalid cognitive tasks.

So, *Y_seed_* is the matrix formed by the columns of *X* corresponding to channels F3 and F4. According to the methodology, these two columns are removed now from the *X* matrix, which now becomes *X_seed_*. Because of these two changes in *X* and *Y*, we obtain a new correlation matrix, the *R_seed_*, which now contains the correlation of each of the j-2 MSE values in *X_seed_* with each of the k seed MSEs in *Y_seed_* within each of the *n* = 3 conditions (ASD, ADHD, control). The *R_seed_* correlation matrix becomes

$$R_{seed}=\left[\begin{array}{c} R_{seed1}\\ R_{seed2}\\ R_{seed3} \end{array}\right]\;$$

where the rows are the seed MSE per condition n (ASD, ADHD, control) and the columns represent the MSE values. The main difference of the seed-PLS approach from the behavior-PLSC is the interpretation, i.e., the relationship now between the seed MSE values (i.e., *Y_seed_*) and the MSE values in *X_seed_* represents their *functional connectivity. The pattern of this connectivity can be illustrated by plotting the saliences for the brain V into a glass brain to show how strongly the seed MSE values correlate with the rest of the brain.* The matrix *U* of the *seed saliences* indicate the differences in the seed MSEs across experimental conditions. Thus, in a similar way as in the behavior-PLSC one, a separate plot of each column of U against each seed MSE shows how the experimental conditions (here the different groups) interact with the seed MSEs. The results of the application of seed-PLSC are presented in Section 3.3.

#### 2.5.3. Graph-Theoretic, Functional Connectivity Measures Applied on Seed Brain Salience Matrix V

In order to enhance the comparison between the functional connectivity matrices generated by the seed PLS, for valid–invalid syllogisms and the different groups of participants, we have carried out a ‘rough’ functional connectivity network analysis, by applying a number of graph-theoretic functional connectivity measures on *weighted undirected matrix*, a transformation of *V*, using the Brain Connectivity Toolbox (http://www.brain-connectivity-toolbox.net, accessed on 8 November 2021) [150]. Functional connectivity analysis for ASD and ADHD subjects, using graph-theoretic measures, has been carried out by Delbruck E. et al. [151], Huang D. et al. [152], and Keon C. et al. [153], the results of which are used in our work to support our findings. Recently, a measure of brain connectivity based on graph signal processing was used to estimate brain region connectivity for epilepsy patients while performing memory and learning cognitive tasks (two tasks that are very ‘similar’ to the cognitive tasks referred in this work) [70]. A set of transition matrices formed via multichannel hidden Markov modeling and graph connectivity theory, used also in our work. The paper also uses partial correlation as a measure to build the connectivity map, as we also have done in our work. 

Our EEG measurements consist of a functional dataset, therefore the nodes of the network are the brain regions (channels) activated by the seeds F3 and F4. Brain salience matrix *V* is the ‘raw’ matrix, as shown in the heat maps of figures in the results section. This matrix is converted to a square matrix with elements that are the correlation coefficients of the pairs of rows (channels) and columns (group-seed, e.g., ASD-seed1-F3). The resulted matrix is called a *weighted undirected matrix* [150], on which a weight threshold is applied to remove weak and non-significant (spurious) links-connections. Finally, we applied some measures of centrality, as follows: (a) the *node degree*, the number of links connected to a node. Since in our case we have only undirected network, no in-degree and out-degree is computed. (b) *Node strength*, the sum of weights of links connected to the node, (c) the *density of a network*, the number of existing connections out of all possible connections, and finally (d) the *betweeness centrality* BC, which identifies which nodes are ‘hubs’, i.e., the nodes that connect with a large portion of other nodes. BC is the fraction of all shortest paths in the network that contain a given node. Nodes with big values of BC participate in a large number of shortest paths. All the results are presented in Section 3.3.

##### Multiscale Entropy MSE

MSE quantifies the complexity of a signal by computing the sample entropy (S_E_) over several timescales on coarse-grained data [47,48]. Sampling entropy measures the order or irregularity of a signal. Let the EEG signal be presented as $=\left\{x_{1},x_{2},\ldots ,x_{N}\right\}$, then S_E_ is defined as

$$S_{E}\left(m,r,N\right)=-ln\left(\frac{c_{m+1}\left(r\right)}{C_{m}\left(r\right)}\right)$$

This expression is the negative of the logarithmic conditional probability that two similar sequences of *m* consecutive data points will remain similar at the next point (*m* + 1). In (7), the term $c_{m+1}\left(r\right)=\frac{\left\{number\;of\;pairs\left(i,j\right)with\;\left|x_{i}^{m}-x_{j}^{m}\right|<r,\;i\neq j\right\}}{\{number\;of\;all\;probable\;pairs=\frac{N-m+1}{N-m}}$ where $\left|x_{i}^{m}-x_{j}^{m}\right|$ symbolizes the distance between vectors $x_{i}^{m}$ and $x_{j}^{m}$, having dimension m, and r is the tolerable distance between two vectors (expressed in respect to st.dev of the signal). N is the length of the time series. So, in case of more regular (e.g., periodic) series

$$c_{m+1}\left(r\right)\approx c_{m}\left(r\right)\;\to 1\;so\;S_{E}\left(m,r,n\right)\;\to 0$$

On the opposite, for a random signal (completely irregular)

$$c_{m+1}\left(r\right)\ll c_{m}\;\to \;\frac{c_{m+1}\left(r\right)}{c_{m}\left(r\right)}\ll 1\;so\;S_{E}\left(m,r,n\right)\gg 1$$

**Figure 5:**
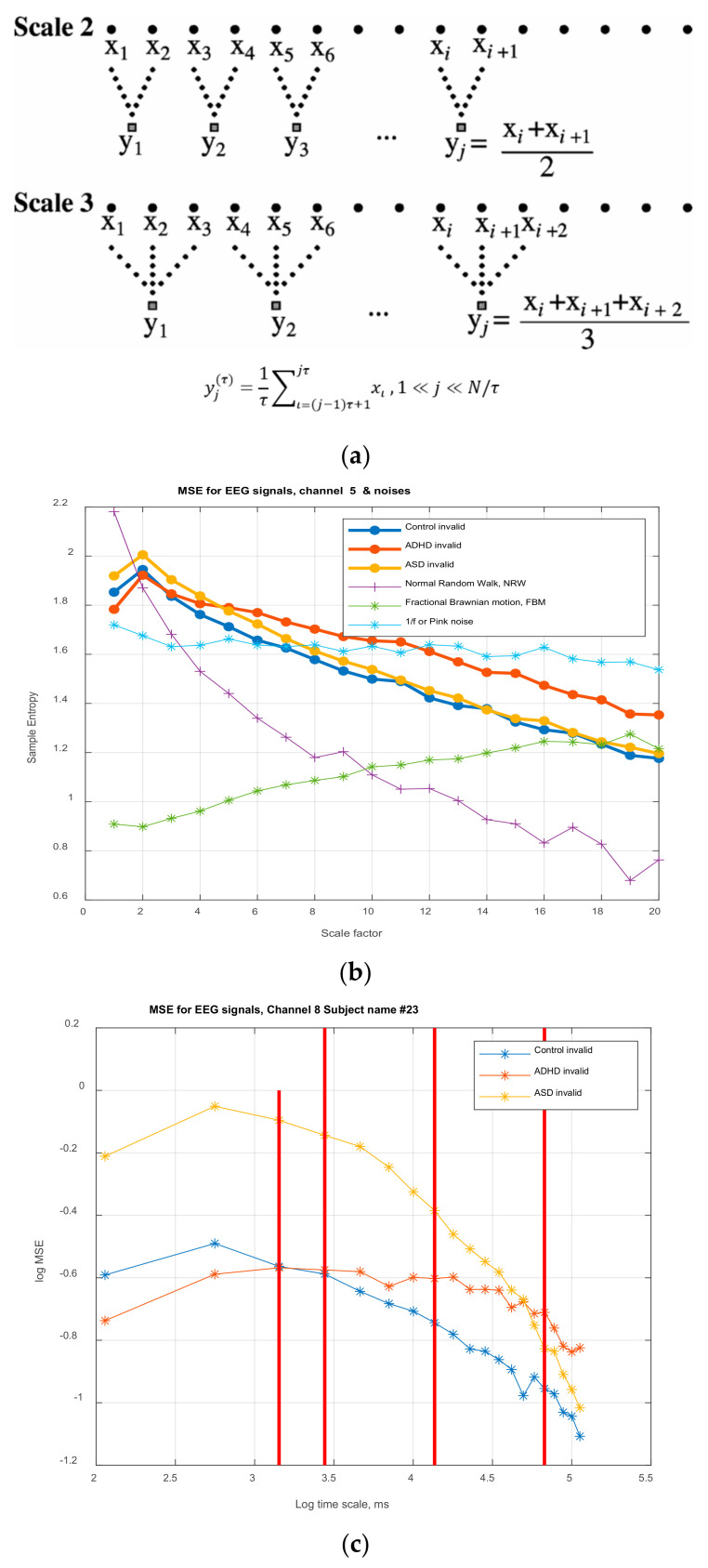
(**a**) The procedure for coarse-graining (Adapted from Costa et al. [47]). (**b**) Comparison of MSE curves (linear x y scales) for the three stochastic noises and three EEGs at channel 5 (indicative), for the invalid type of syllogism, and for the three groups of subjects (control, ASD, and ADHD). (**c**) MSE curves vs. time scales, in log–log scales, for subject #23 and channel 8, for invalid type of syllogism. The red line set the borders of frequency bands (gamma, beta, alpha, theta, and delta). (Taken from Papaioannou A. et al. [155]).

For the computation of MSE, the original signal $\left\{x_{1},x_{2},\ldots ,x_{Ν}\right\}$ is transformed to a coarse-grained signal $\left\{y^{\left(\tau \right)}\right\}$, where τ is the scale factor (SF). The procedure for coarse-graining is shown in Figure 5a, adopted from Costa et al., 2005 [47]. The original series is divided into non-overlapping windows of length, and the points inside the window are averaged, so a coarse-grained series is obtained as

$$y_{j}^{\left(\tau \right)}=\frac{1}{\tau }\;\sum_{i=\left(j-1\right)\tau +1}^{j\tau }x_{i},\;1\leq j\leq \frac{N}{\tau }$$

##### $S_{E}$ is computed for each time-series $y_{j}^{\left(\tau \right)}$

Based on previous studies, $S_{E}$ has a good statistical validity for parameters m = [1, 2] and 0.1 ≤r≤0.25 [154]. In the present study, m = 2, r = 0.15 × st.dev, N = 6,0000 samples, and τ = 20 scale factors, so Ν/τ =3000 samples, therefore, enough to obtain a reliable estimation of $S_{E}$ [154].

In Figure 5b,c, we provide indicative MSE curves, as an example, that computed the invalid case of syllogism for the groups ASD, ADHD, and control. We also show MSE values for various noises for comparison (taken from Papaioannou A. et al. [155]). We must note here, however, that in the present work we used average MSE values, at all 14 channels, and across all scale factors (average of 20 scales). So, for each participant we have 28 Channel-syllogism MSE values (14 for valid and 14 for invalid), in each group.

Figure 5b shows three MSE values (for the invalid type of syllogism, and for the three groups of subjects, at channel 5) ‘generated’ by EEG activity at channel 5 as well as MSE values extracted from various noises, for comparison. We observe that up to scale 5, the complexity of EEG from ASD subjects is larger than that of both ADHD and control ones, while after scale 5, the complexity of ADHD is higher. *This indicates the advantage of using multiscale entropy measure instead of using the ‘typical’, simple, one-scale entropy that does not take into account that the* dynamics of EEGs are dependent also on the time-scale (new mechanisms activated at different timescales on the ‘scene’ and affect the dynamics of EEG). We note here that for the purpose of the present study, taking the grand average of MSE as mentioned before, does not undermine the efficiency of the MSE method and the reliability of its results, since by using MSE values per participant instead of mean EEG values as input in the PLSC analysis, produces better results, i.e., the extracted correlation between brain data and behavioral data is more reliable, as is shown below. 

Figure 5c depicts exemplary MSE curves vs. time scales, in log-log scales, for subject 23 and channel 8, for all groups and valid and invalid types of syllogisms, are shown. The red line set the borders of frequency bands (gamma, beta, alpha, theta, and delta). As it is shown in the figure, in *gamma* frequency region (gamma rhythm), group ASD presents the highest value in MSE for invalid type as also in the case of valid type (not shown here), in which MSE of ADHD is larger than that of ASD. In *beta* region, MSE values for valid and invalid are different. In this figure, both control and ADHD EEGs have identical MSE values, while in the valid type MSE od ASD is larger than that of ADHD, which is larger than that of control [151].

## 3. Results

### 3.1. Mixed ANOVA and Multi-Dimensional Chi Square Analysis of MSE Values

Statistical analysis was carried out using SPSS Statistics v20.0 software for Microsoft Windows, with alpha significance set at 0.05. We tested the dependent variable for multicollinearity, before proceeding to ANOVA, and we found no pairwise correlation coefficients larger than 0.90 [156]. To examine simultaneously the *between and within subjects factor* variations of the *dependent variable MSE*, we employed a three-way mixed ANOVA or 14 × 2 × 3 design to study the within-subjects effects and between-subjects effect of ASD, ADHD, and control groups. The first factor is the within-subjects factor *channel*, with *14 levels*. The second factor is also a within-subjects factor of *Syllogism*, with *two levels (valid, invalid)*. The *third factor is between-subjects factor of group*, with *three levels*: ASD, ADHD, and control. The main results are shown in Table 4, and Table 5 below (the rest of the tables of the test are in the Supplementary Materials).

Table 4 indicates that the main effect of *group factor is significant* (F_(2,60)_ = 3.564, *p =* 0.034). The Mauchly’s test of sphericity W = 0.022 is significant (*p =* 0.000), so the assumptions behind the normal within-subjects ANOVA are violated, thus the reported F is epsilon corrected and the *p* value is also the corrected *p* value. 

From the tests within-subjects effects (see Table 5), the *main effect of channel factor is* significant (F_(13,780_ = 12.677, *p =* 0.000), and the channel * group interaction is also *significant (*F_(26,780)_ = 2.677, *p =* 0.000). The *syllogism main effect* is not significant, F_(1,60)_ = 2.263, *p =* 0.138. Also, the syllogism* group interaction is not significant, F_(2,60)_ = 0.985, *p =* 0.379. The channels* syllogism interaction is not significant, F_(13,780)_ = 0.983, *p =* 0.466. Finally, the channels* syllogism*group interaction is not significant, F_(26,780)_ = 1.103, *p =* 0.329

We have estimated also the effect size for each main effect and the interactions, by calculating the partial eta squared (η^2^), in order to quantify how large each effect is. The significant *main effect of channels* has η^2^ = 0.174, indicating that this variable, when removing the effects of the other variables and interactions, explains 17.4% of the variance. Similarly, the significant two-way interaction of channels * group has η^2^ = 0.082 and explains 8.2% of the variance. No other effects are significant.

From Table 6, we see that there are *significant differences* between the means of MSE (complexity) values at the 14 channels of the members of the ‘pathological’ groups of participants, i.e., between ASD and ADHD, while the *difference in means of MSE between both ASD and ADHD and that of the control group* is *not significant*. In conclusion, the valid–invalid type of syllogism generates significantly different complexity values, MSE, between ASD and ADHD. However, when passing from valid to invalid cognitive load in the ASD group, the complexity increases, a trend not shown in the cases of ADHD and control.

Since the group*channels interaction was found to be significant, we examine more carefully the largest values of MSE at each channel, for each group. For the ASD group, channels FC6 (Frontal-Central right) and AF4 (Anterio-frontal right) have very large values, indicating that at these regions the EEGs activity is very volatile and complex (strong nonlinear and linear interactions prevail). Similarly, in ADHD channels F4 (Frontal-right) and O1 (Occipital, left), the complexity is also very high. Finally, in the control group, channels F3 (Frontal-left) and O1 (Occipital-left) are very complex and volatile. Therefore, the valid–invalid cognitive tasks have ‘stimulated’ both different as well as the same brain regions (e.g., the occipital-left one is commonly ‘stimulated’ in both ADHD and ASD participants).

**Figure 6:**
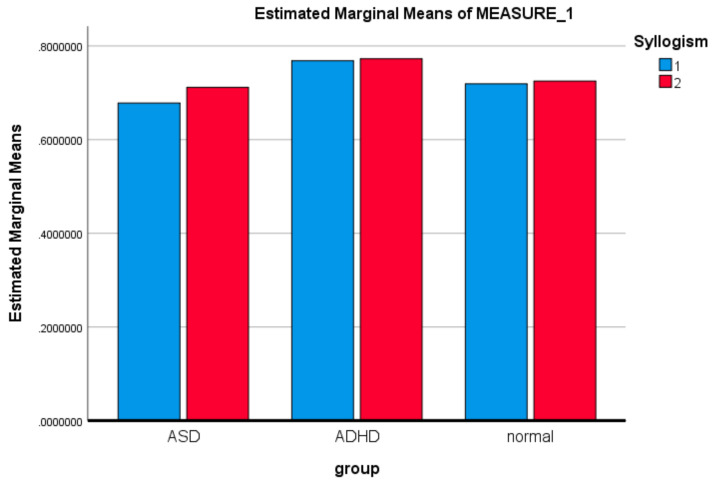
Marginal means of complexity (MSE), for each group of participants for valid and invalid syllogisms. When passing from valid to invalid cognitive load in the ASD group, the complexity increases.

As we observe in Figure 6, the entropy (mean MSE) was higher in the ADHD than in control, which in turn is slightly higher than in the ASD group, especially for the valid syllogism. Enhanced brain signal variability was observed in the work of [157] in an experiment to test child’s cognitive ability by using MSE, between ASD and control groups. They found a significant group effect in *parietal* and *temporal* regions and a significant group*MSE’s scale factor parameter effect, in *frontal, central* (FC6 in our case), and temporal regions, using an ANCOVA design. We remind here that in our case we have taken the average MSE across all scale factors (mean over 20 scales), so here we do not have such an effect. However, our other results that are also in accordance with their work could be considered, regarding the found significant group*channel effect in the frontal-central right region FC6 in ASD.

#### 3.1.1. Behavioral Performance: X^2^ Multidimensional Test

We tested the dimensions of emotions of participants (*strength, arousal, and control*) for both valid and invalid syllogisms. The data are ordinal, so we test for the *association* and *difference* of the related variables between the independent variable: group, smoking, left- and right-handed, health treatment and issues, education, and occupation. The only significant interactions were found as shown on Table 7.

**Figure 7:**
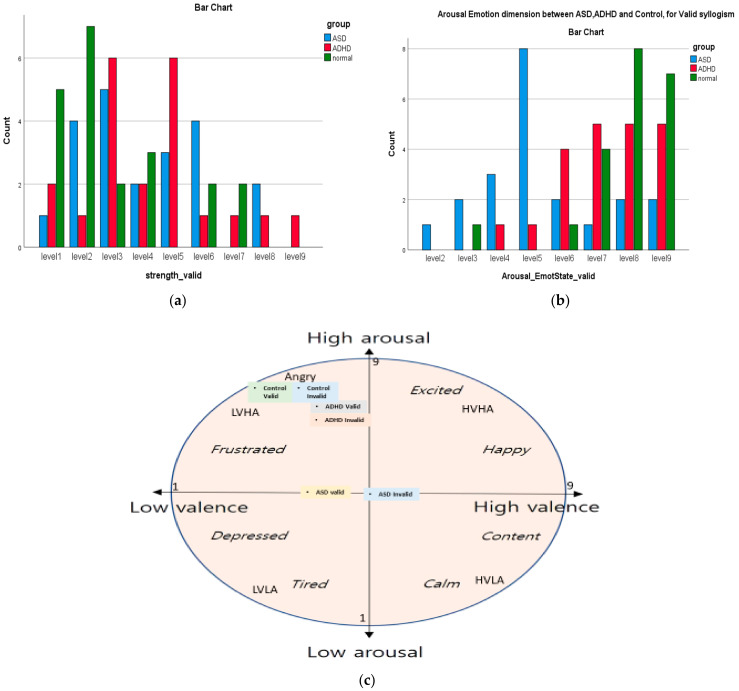
Variation of arousal (**a**) and valence (strength), (**b**) dimensions of emotion scores with groups, (**c**): 2-D plot of the arousal and valence (strength) dimensions of emotion scores, for the three groups [100,101]. The plot is based on the median values of Table 8.

**Figure 8:**
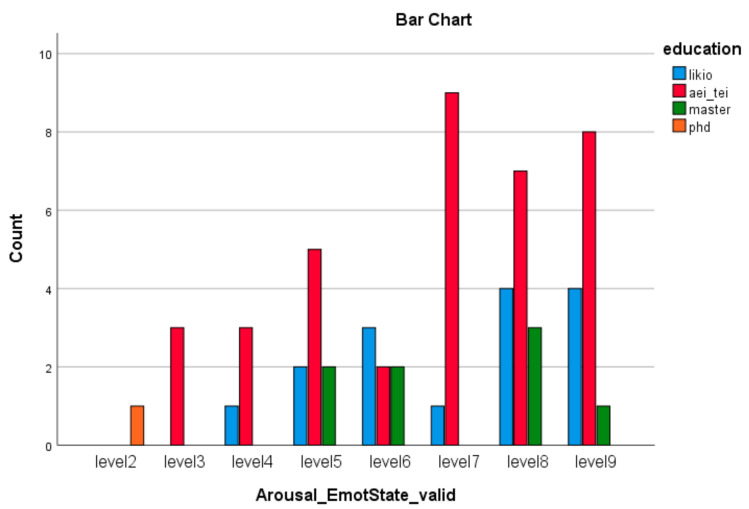
Arousal dimension of emotion scores versus education levels of participants in the groups.

**Figure 9:**
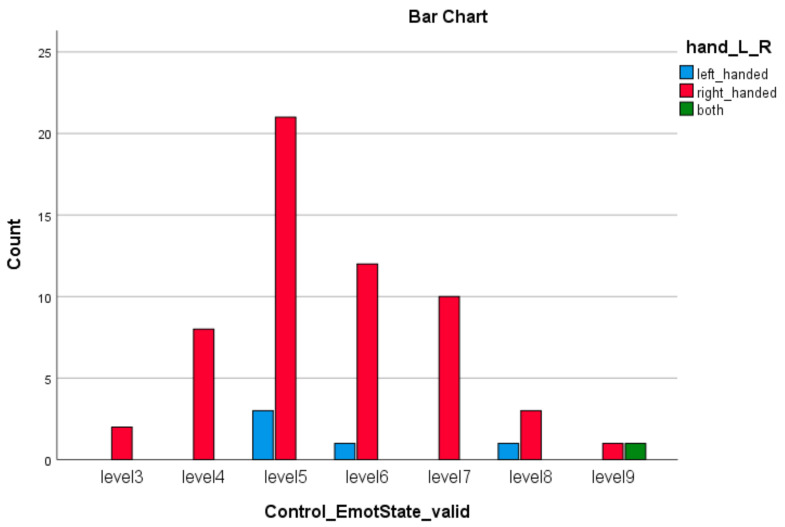
Control dimension of emotion scores versus handedness (right or left-handed participants) in the groups.

From Table 7, for the valid syllogism, there is a significant relationship between *arousal emotion* (one of the three dimensions of the emotional state construct), and *group* (*Χ^2^* = 31.08, df = 14, *p* = 0.05). Also, there is a significant relationship between *arousal emotion* and *education* (*Χ^2^* = 72.76, df = 21, *p* = 0.000). We observed also a significant relationship between *control emotion* and the *left- or right-handed* feature (*Χ^2^ =* 35.00, df = 12, *p* = 0.000). No associations were found between emotion dimensions and all groups for the invalid syllogism. Figure 7, Figure 8 and Figure 9 depict the variation of the above interactions with group. We comment below on these findings, referring to relevant literature for support. Based on Figure 7 and Table 8, which shows the self-reported scores of the participants on the valence and arousal dimensions of their emotional state during the reasoning tasks, we have constructed Figure 7c, the 2-D plot of the *circumplex model of affect* [100,101]. From the plot, we observe three distinct clusters: *(a) the ASD valid–invalid characterized by low valence medium arousal and medium valence medium arousal respectively, (b) the ADHD valid and invalid, characterized by low valence high arousal (LVHA), and (c) the control valid–invalid, with low valence, high arousal (LVHA).*

An interesting finding in our study is the significant association (Table 7) between *control of emotion* and *handedness*. From Figure 9, we see that the right-handed participants scored high in levels 5, 6, and 7 of the SAM scale for the control dimension of the emotional state measure. It is well known that, since handedness is a reflection of *lateralization on the nervous system*, it is very useful to study the relations between *handedness*, *cerebral lateralization,* and cognition. Different kinds of information processing require the activation of specific sides of the brain—the left and right sides. Moreover, our cognition is lateralized partially toward one side or the other. Therefore, the detection of a “connection” between this findings with the MSE values of ASD, ADHD, and control, for the valid–invalid cases, would be a challenging result. A recent work that sought differences in cerebral cortical anatomy of left- and right-handers [158], concluded that no individual cortical region showed an association with left-handedness. In our work, the majority of participants stated that they are right-handers (73 out of 81, i.e., 90%), (27 ASD, 26 ADHD, and 20 control), while 1 ASD, 3 ADHD, and 1 control were left handers (7%), and 3 both-handers. Regarding handedness and ADHD-ASD disorders, Simons et al. [159] have shown that left-handed ADHD students exhibited higher number of errors and higher variability of reaction times as compared with the controls. They also show a significant effect of handedness on commission errors (CE), since left-handers made more CE than right-handers. On the other hand, Wiberg et al. [160] provided evidence that imaging-handedness analysis revealed an increase in functional connectivity between left and right language networks in left-handers. 

### 3.2. Behavior-PLSC and Seed-PLS Results

#### 3.2.1. Brain Saliences Scores

**Figure 10:**
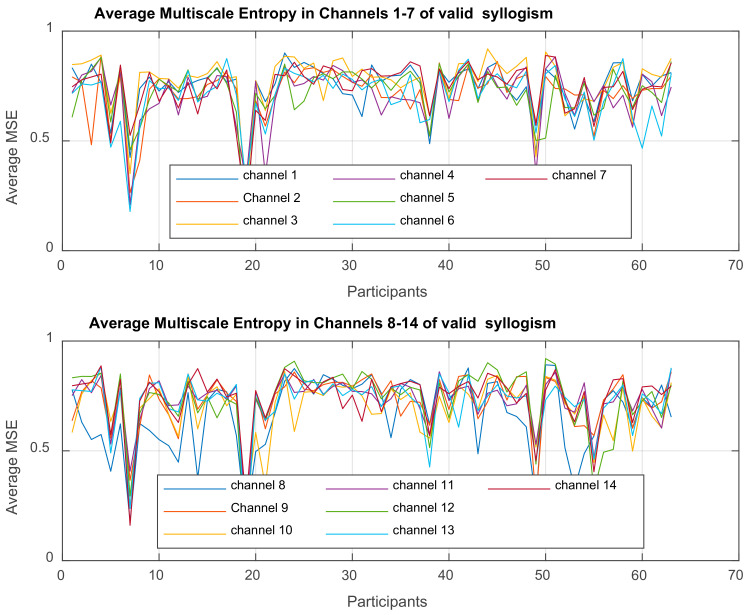
Time series of average MSE values at channels per subject, extracted from EEGs recorded at participant’s scalp, when they are exposed in a cognitive load induced by a **valid** syllogism.

**Figure 11:**
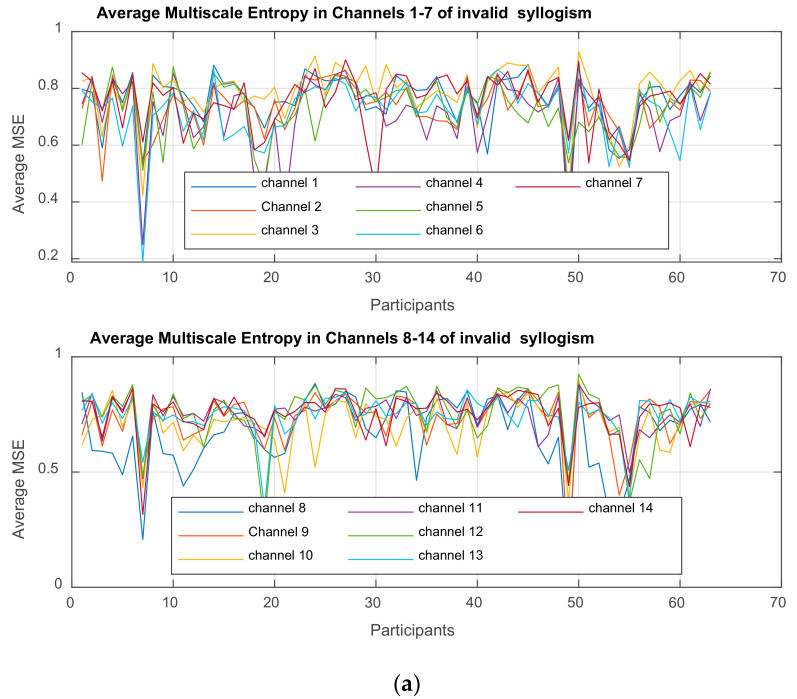

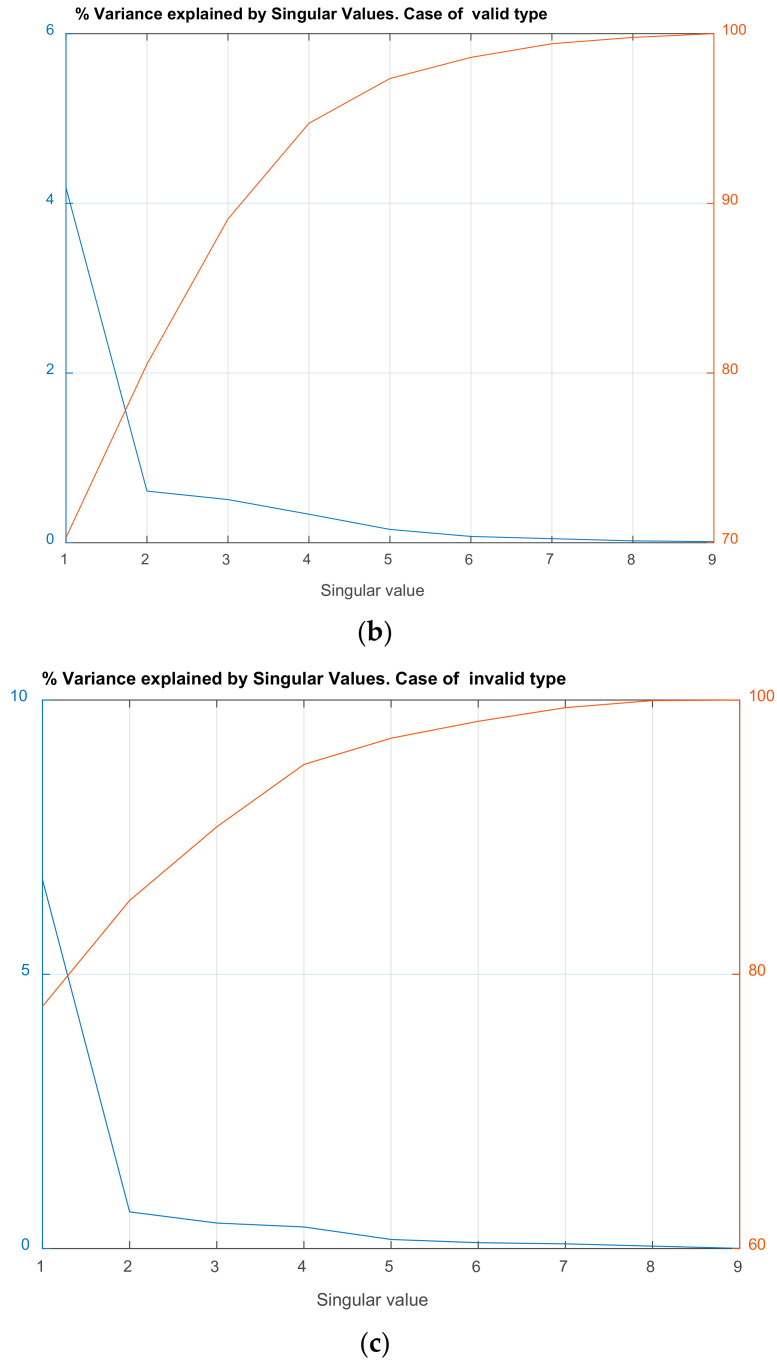
(**a**) Time series of average MSE values at channels per subject, extracted from EEGs recorded at participant’s scalp, when they are exposed in a cognitive load induced by an **invalid** syllogism. (**b**) Plot of squared singular values and percentage of the total variance explained. The first four singular values explain about 95% of the variance. It shows actually the effect of *dimensionality reduction* attained by the action of the SVD on the Correlation matrix *R.* Case: Valid syllogism. (**c**) Plot of squared singular values and percentage of the total variance explained. The first four singular values explain about 95% of the variance. It shows actually the effect of *dimensionality reduction* attained by the action of the SVD on the correlation matrix *R.* Case: invalid syllogism.

Figure 10 and Figure 11a show the time series of average MSE values at all channels per subject, extracted from EEGs recorded at participant’s scalp, when they are exposed in a cognitive load induced by a **valid** and **invalid** syllogism, respectively.

Figure 11b shows the results of applying the SVD on matrix *R*, regarding the singular values squared (since singular values are equal to square root of eigenvalues), and the percentage of the total variance of *R* explained. The sum of the first four singular values squared, explain around 95% of the variance. Table 9 lists the singular values and their percentage of variance explained for valid and invalid cases. Figure 11c shows similar results for the invalid case.

**Figure 12:**
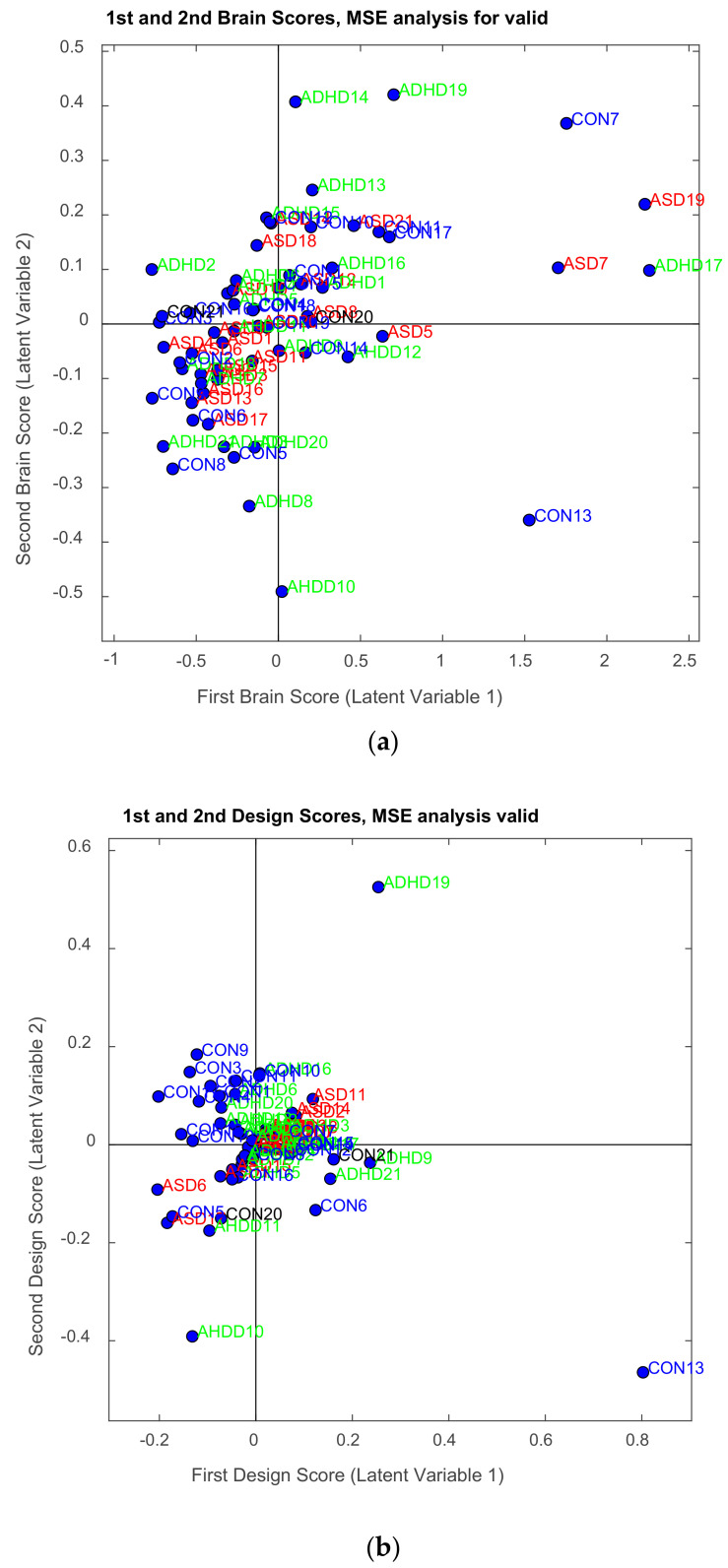
(**a**) Plot of first and second brain scores (latent variables), based on the average per subject MSE, for **valid** type of syllogism. (**b**) Plot of first and second **behavior** (Design) **scores** (**latent variables**), based on the average per subject MSE, for **valid** type of syllogism.

**Figure 13:**
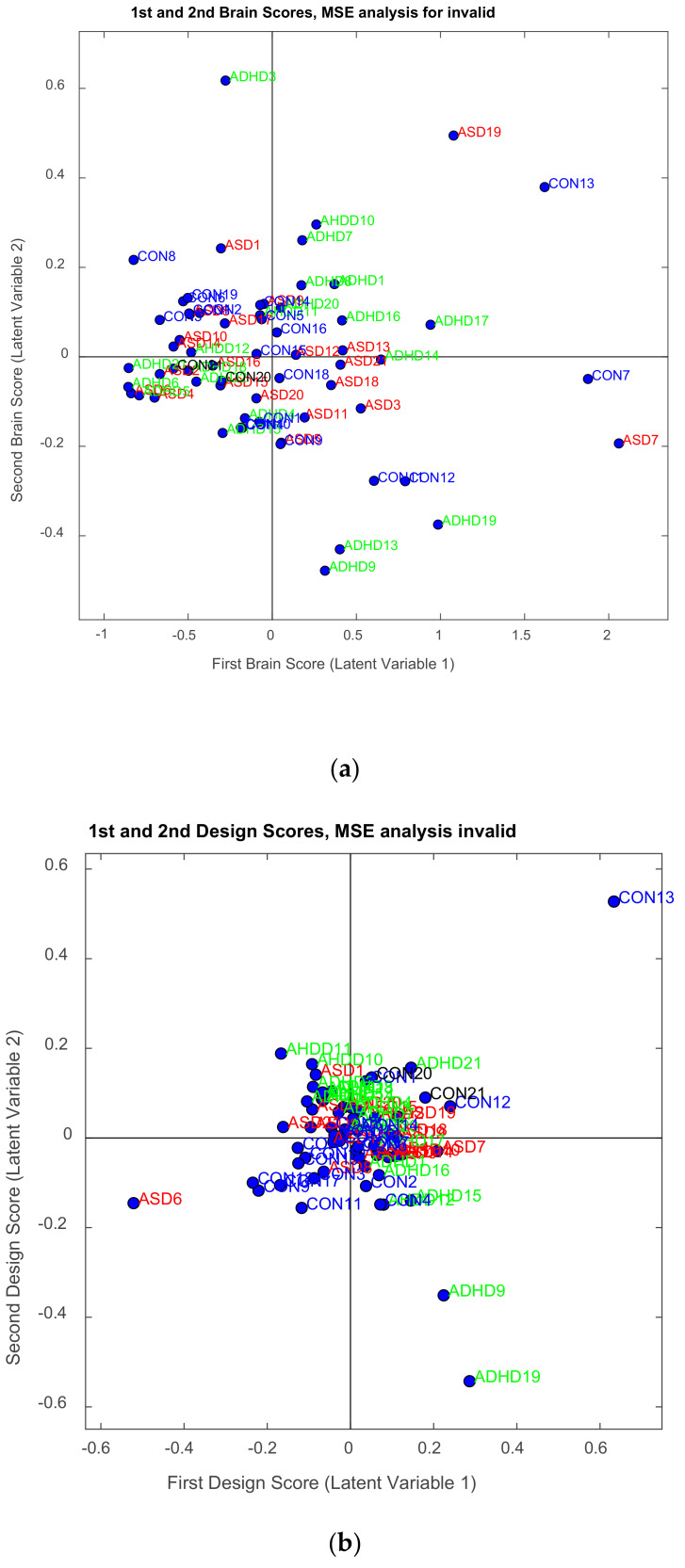
(**a**) Plot of first and second **brain scores** (**latent variables**), based on the average per subject MSE, for **invalid** type of syllogism. (**b**) Plot of first and second **design scores** (**latent variables**), based on the average per subject MSE, for **invalid** type of syllogism.

In Figure 12 and Figure 13, we plot the *first and second brain scores (latent variables)* and *behavior (design) scores*, extracted from the application of PLSC on MSE values, for valid and invalid type of syllogism, respectively. They show the relationship of the covariance of the brain scores. *The plots are PCA style plots and show how the first or second brain or design scores separate some participants from the other.* The latent variables reflect the relationship of the covariance of the brain score. Distance on the plot will directly indicate the amount of explained covariance of the correlation matrix *R* (the further from the origin an item the participant is, the more variance it contributes to the visualized latent variables). Figure 12a, for the valid case, shows four quadrants, looking clockwise the 1st, 2nd, 3rd, and 4th. Groups of participants on the 1st quadrant are positively correlated to other groups. We observe in the figure that the majority of ASD participants is concentrated mainly on the 3rd quadrant (negatively correlated) and also distributes along the diagonal from 3rd to 1st quadrant, the axis of a fictitious ellipsoid of distribution of the participants, with a ‘center of mass’ close to the origin. Participants of the control group are mostly concentrated also around the origin, indicating that they contribute symmetrically to the total covariance of the brain dynamics. In Figure 12b, in the valid case we show the plot of the scores of first and second behavior latent variables (i.e., age, level of confidence and emotional control). It shows actually the relationship of their covariance. The extent of the distribution of the participants around the origin is smaller and denser, in comparison with the previous figure, with control group mostly concentrated on the 4th quadrant, ASD along the diagonal axis from 3rd to 1st quadrant but close to the origin, while ADHD mostly on the 1st and 2nd quadrants. Overall, figure shows that *the contribution of the participants to the total covariance of the behavior data is more dense and closer to the origin compared* with that of Figure 12a. In Figure 13a, in invalid case, the majority of ADHD and control subjects are concentrated along the diagonal 2nd to 4th quadrants and close to the origin, exhibiting an *asymmetric contribution to the total variance*. ASD subjects are located closer to the origin, while ADHD further from the origin, producing a greatest contribution to the variance. ADHD participants are concentrated along the diagonal 3rd to 1st quadrants, with the majority of them located at the first and positively correlated to the other groups. Figure 13b, also an invalid case, is very similar to Figure 12b, but here all participants are more densely concentrated around the origin.

#### 3.2.2. Behavior Saliences Scores: Age-Group, Emotion State-Group, and Level of Confidence-Group Interactions

**Figure 14:**
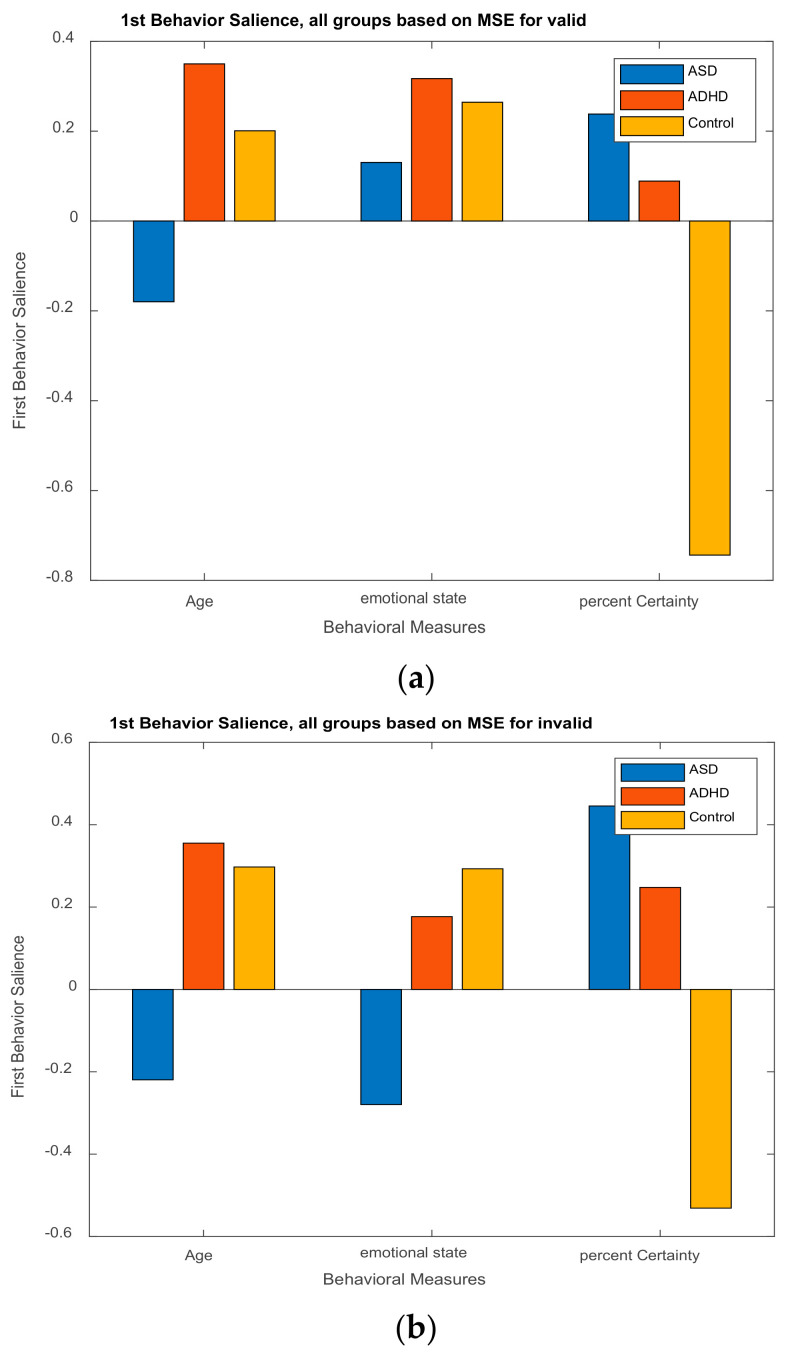
(**a**) 1st behavior salience, **valid** syllogism. The 1st behavior salience reveals the difference ASD and the other groups for age (‘maturation effect’) and the difference between control and other groups for percent of certainty (or degree of confidence). No difference was found in emotional state for all groups. (**b**) 1st behavior salience, **invalid** syllogism. The 1st behavior salience reveals the difference ASD and the other groups for age (as in the valid case) and the difference between control and other groups for percent certainty and difference between ASD and the other two groups in the emotional state.

**Figure 15:**
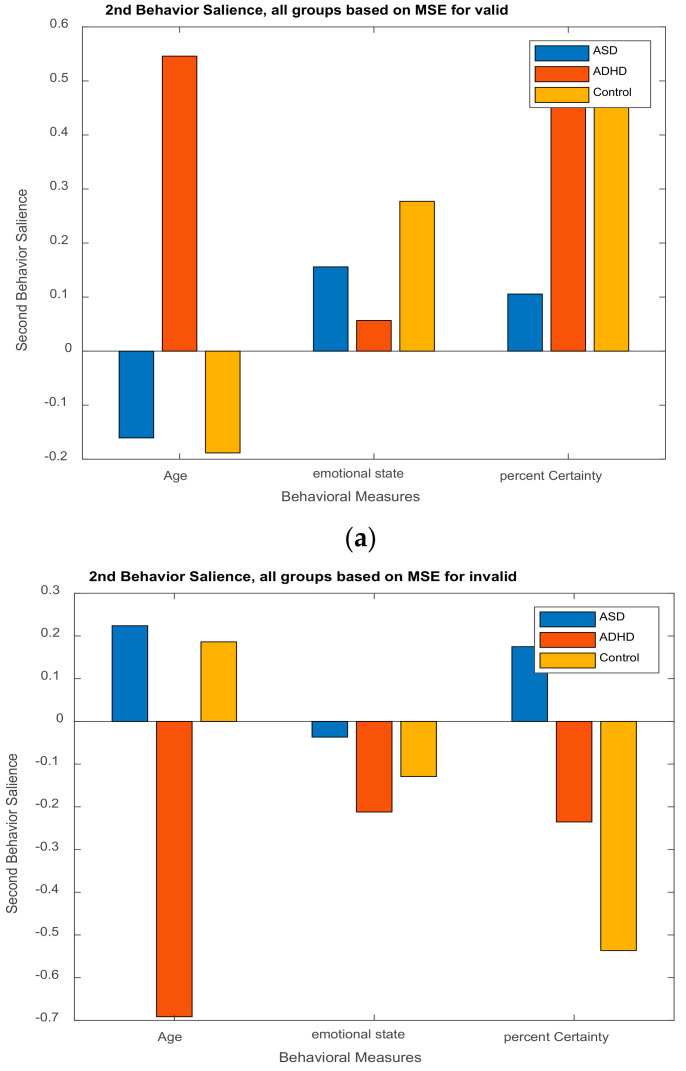
(**a**) 2nd behavior salience, **valid** syllogism. (**b**) 2nd behavior salience, invalid syllogism. This salience reveals the difference between ADHD (strong negatively correlated) from ASD and control for age, no differences in emotional state, and difference between ASD and other two groups for percent certainty, having also different signs in correlation.

We have observed in Figure 14a,b (for valid syllogism) and Figure 15a,b (for invalid syllogism) that age has a significant effect in separating groups of participant, in the first and second behavior saliences. We will try at this point to explain this finding by using results from similar PLSC behavior results. Ziegler et al. [43] used PLSC to analyze the correlation of multivariate cognitive abilities (matrix X) and local brain structure (matrix Y) in children and adolescents. Using specific cognitive weightings, in order to improve the age-group covariate analysis (i.e., to optimize the extraction of singular values), Ziegler et al. [43] analyzed the effects of maturation, i.e., *the age-related differences of the brain (structure) data and cognition data variance.* They studied how the multivariate structure- cognition covariance evolves as a function of age. They found evidence of dynamic changes of the multivariate brain structure-cognition covariance as a process of age or brain maturation. More specifically, by using three age groups, they analyzed the maturation of the structure-cognition covariance, by observing how the latent variables (or saliences) scores correlation with cognitive abilities are changed in respect to the three age groups. Therefore, our findings about the age ability to separate strongly ASD from ADHD and control in the valid type of syllogism in the first and second saliences is in accordance with the above maturation effect in the work of Ziegler, although the experiments are different. The “accordance” of the results points out the capacity of the PLSC model to extract such hidden correlational association between behavioral and brain data. 

This salience reveals the difference between ADHD (strongly positively correlated) from control and ASD (negatively correlated) for age and shows no differences for emotional state and percent of certainty. ADHD and control are strongly and positively correlated for percent certainty, also all groups are positively correlated for the emotional state.

Based on Section 3.2.1 and Section 3.2.2, we are now able to draw a general conclusion: the brain and behavior first and second latent variables indicate that the participants of the three groups contribute differently to the total covariance of their interaction as described and quantified by the brain–behavior correlation matrix, in the valid and invalid cases of syllogism. This general result (even though not so discernible due to overlapping of the points on the graphs brain dynamics latent variables) in combination with the results shown in the figures of 2-D plots of the behavior saliences (Figure 14 and Figure 15), enhances our argument that the extracted brain and behavior latent variables via PLS have properly captured the shared information or ‘hidden’ correlation between the two data sets and that these latent variables can distinguish adequately, in valid and invalid syllogism, the interaction of the dynamics of the brain of the participants belonging in ASD, ADHD, and control groups, as a result of the cognitive loads induce by the valid–invalid Aristotle’s syllogism, with the behavior measures of the participants, recorded during this experiment.

#### 3.2.3. Projections of the Brain–behavior Correlation Matrix on Channels. The ‘Heat Map’

**Figure 16:**
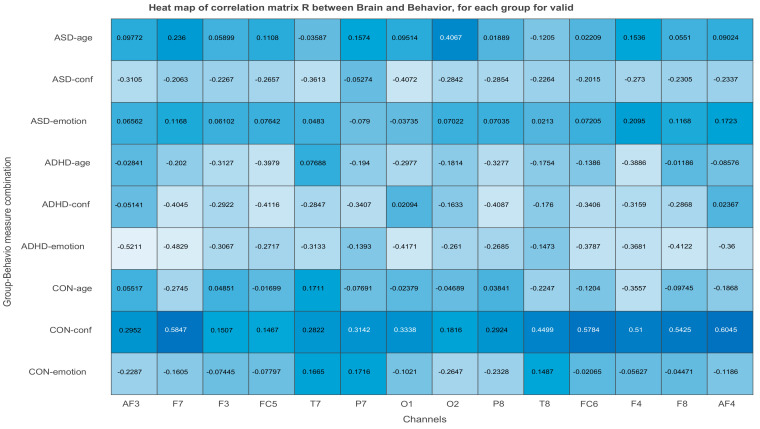
Heat map of the correlation matrix ***R*** between brain and behavior matrices for the **valid** syllogism.

**Figure 17:**
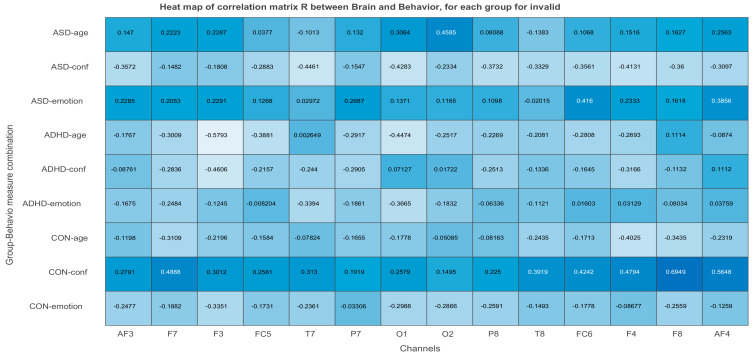
Heat map of the correlation matrix ***R*** between brain and behavior matrices for the **invalid** syllogism.

It is very interesting to see how the aforementioned behavior*group interactions are related to the electrode locations (channels), i.e., how the MSE values, extracted from the EEGs at these brain locations reflecting their activation due to valid–invalid syllogism, are correlated to group*behavior interaction. Figure 16 and Figure 17 show the ‘heat map’ (i.e., strength of correlation) of the matrix *R* for valid and invalid, respectively, in which rows represent correlations and columns the channels. A more informative figure than the heat map presented in this section, is a figure that shows the weights of the MSE dependent differences in the brain–behavior correlations on the brain locations (channels), described in the next section.

In the valid case, we observe in Figure 16, three clusters (age-ASD, emot-ASD, and conf-CON-) which exhibit strong correlations between MSE values (brain dynamics) and behavioral dynamics, expressed by the *emotion state* and *age* of ASD and *level of confidence* in answers of the control group, in almost all channels. More specifically, the correlation of the emotional state of control group with MSE values is ‘high’ (>0.50) and positive at channel F7 (frontal temporal left), while the correlation of level of confidence of the control group with MSE values positive and strong at channels FC6 (right, frontal central), F4 (right, frontal), and F8 (right, frontal-temporal). Less and negative correlations exist between MSE and emotion state of ADHD group at F7 (left, frontal temporal) and O1 (left occipital), at which also the confidence level of ASD is negatively correlated with MSE.

In the invalid case, Figure 17, we observe weaker correlations than in the valid case, and also concentrated in a smaller number of channels. Actually, the correlation of the level of confidence of the control group is positively but ‘moderately’ (<0.5) correlated with MSE values at channels F4, F7, F8, and AF4, and negatively at FC6. Only the age of the ADHD group is high and positively correlated with MSE at F3, at which also the level of confidence of ADHD is moderately and negative correlated. Finally, the emotion state of ASD group is moderately correlated with MSE at FC6.

Therefore, the heat map of R reveals that in the case of invalid syllogism, the number of activated brain regions that are significantly correlated with the behavior measures of the subjects of three groups is smaller than in the case of valid syllogism. An interesting finding also is that in both valid and invalid syllogisms, the correlations of the level of confidence of the control group are positive and happened at the same frontal lobe channels: FC6, F4, F8, and AF4. 

#### 3.2.4. Projection of the Brain Saliences Matrix V on Channel (Brain) Locations

**Figure 18:**
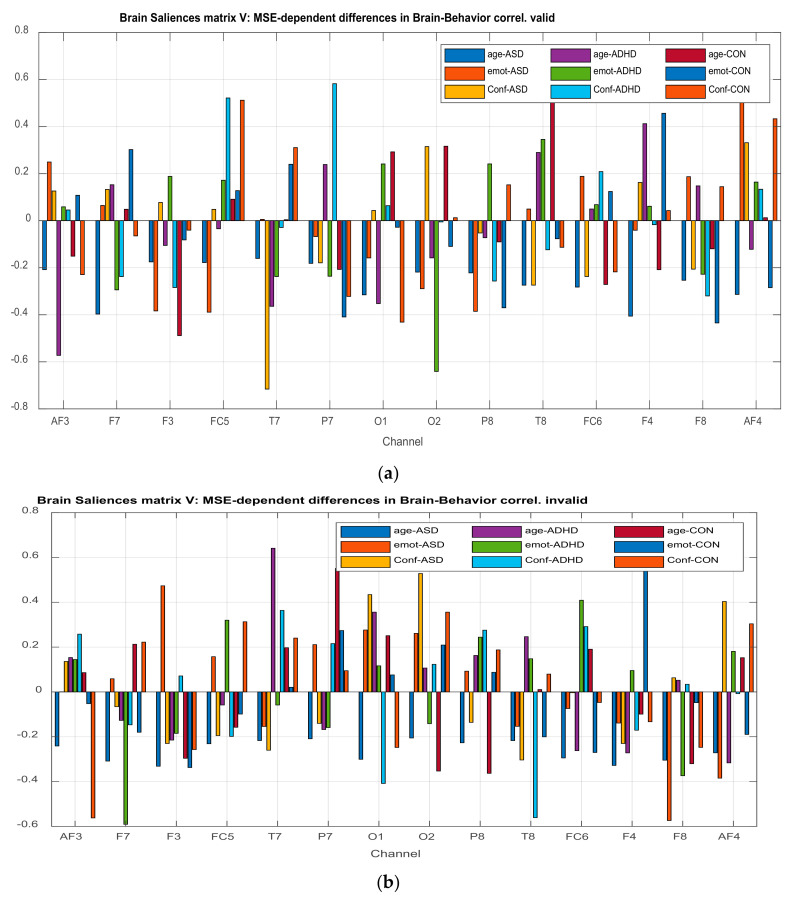
(**a**) Plot of saliences for brain matrix ***V*** (right singular vectors) row-wise. Each plotted row corresponds to a channel and depicts how the MSE dependent differences in brain–behavior correlations weigh on that channel, in the case of **valid** syllogism. (**b**) Plot of saliences for brain matrix ***V*** (right singular vectors) row-wise. Each plotted row corresponds to a channel and depicts how the MSE dependent differences in brain–behavior correlations weight on that channel, in the case of invalid syllogism.

**Figure 19:**
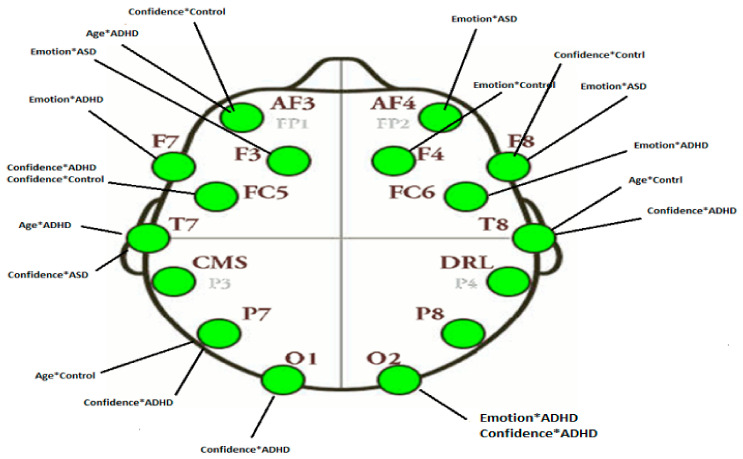
Location of intense *behavior by group interaction effects* (correlations >0.40), in both valid and invalid syllogism.

A similar, but more informative, figure to the heat map presented in the previous section, is a figure that shows the weights of the MSE dependent differences in the brain–behavior correlations on the brain locations (channels). Actually, this is a projection of the brain salience matrix *V,* after performing the singular values decomposition SVD (see Section 2.5). Figure 18a,b, show these projections for the valid and invalid cases, respectively. For example, in the valid syllogism, at channel T7 (Temporal left) the (behavior-group interaction) *percen*t of confidence * ASD is strongly negatively correlated with MSE, while at channel P7 (Parietal left) the interaction percent of confidence * ADHD is strongly positively correlated with MSE. To facilitate the reading and interpreting of the figures, we have formed Table 10, based on these figures, showing notable high correlations (>0.40) between pair of brain–behavior measures (extracted from brain saliences matrix *V*). The elements of this table have been entered on the scalp locations of the channels, in Figure 19. We observed that region above T7-T8 symmetry line (lateral-medial-lateral axis or the coronal plane cross-sectional line) is dominated mainly by the *emotion of ‘pathological’ groups* and the *confidence of control group,* while the region below this line is dominated mainly by *degree or level of confidence of ‘pathological’ groups.*

### 3.3. Results of Seed-PLS for Functional Connectivity

We performed a full-group seed-PLS functional analysis. The squared singular values and the associated total variance explained in the case of valid and invalid syllogism are shown in the Table 11. We observe that the first latent variable (LV) in the valid case explains 98% of the total variance of the MSE values in the brain regions that are activated by the seed regions F3 and F4. For the invalid case, the first LV explains almost 95% of the total variance. We concentrate therefore only on the first two latent variables (or first and second brain saliences).

**Figure 20:**
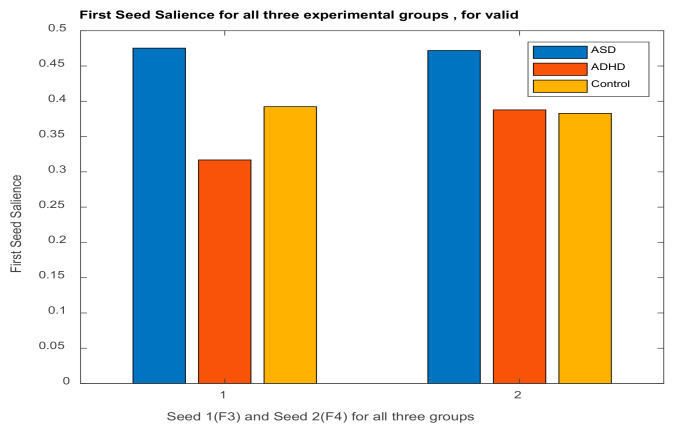
Plot of first seed salience vs. seeds 1 and 2 (MSE values at F3 and F4, respectively), for all groups, in the valid syllogism.

Figure 20 show how the first and second saliences differentiate the groups. The figure shows the first seed salience where the first seed, i.e., MSE values at channel F3, indicate that ASD group exhibits larger (+) correlations than the other two groups, with the control group to have the second larger correlation. The second seed salience, shows the same pattern, i.e., ASD group has the largest (+) correlation, with the other two to have almost the same correlations.

**Figure 21:**
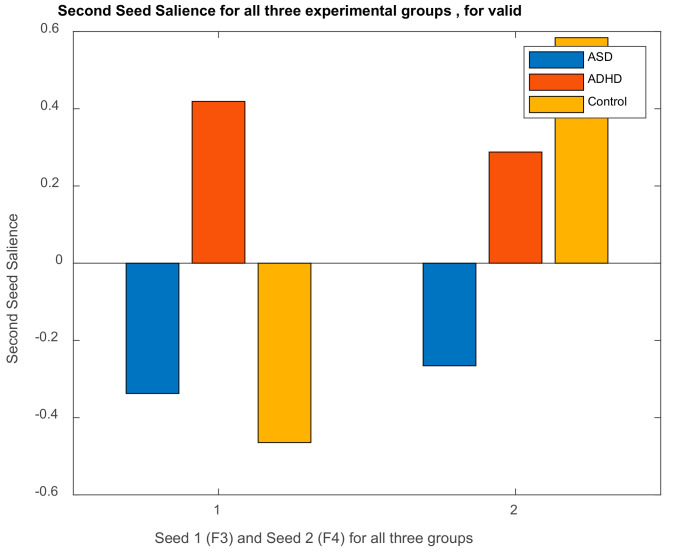
Plot of second seed salience vs. seeds 1 and 2 (MSE values at F3 and F4, respectively), for all groups, in the valid syllogism.

Figure 21 shows that the second seed salience ‘does a better job’ in differentiating the groups. The first seed here (MSEs at F3 or left-DLPFC) differentiate ADHD group from the other groups, and the second seed (F4, right-DLPFC) differentiate the ASD group from the other two groups. 

**Figure 22:**
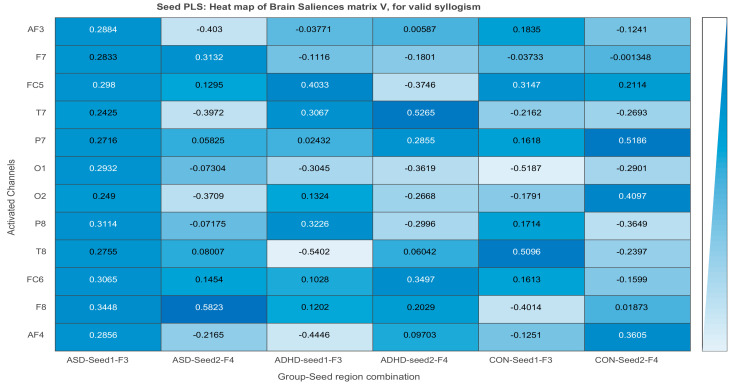
Heat map of the brain salience matrix V extracted by SVD of correlation matrix *R* between brain *X* and seed *Y* matrices, for the valid syllogism.

**Figure 23:**
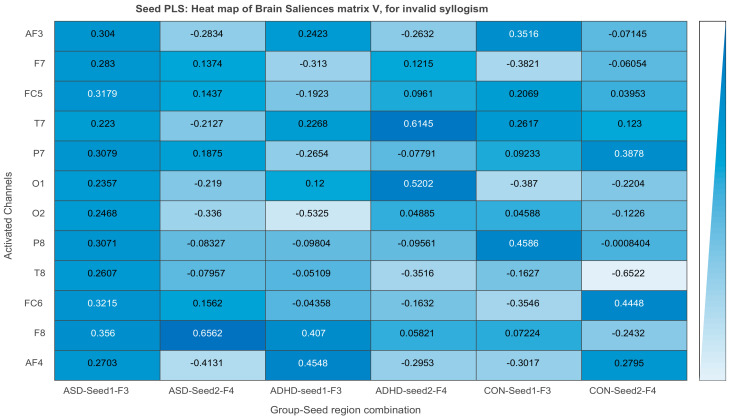
Heat map of the brain salience matrix V extracted by SVD of correlation matrix *R* between brain *X* and seed *Y* matrices, for the invalid syllogism.

Figure 22 and Figure 23 depict the ‘heat map’ of the brain saliences matrix V extracted by SVD on the correlation matrix *R* between brain *X* and seed *Y* matrices for the valid and invalid syllogisms, respectively. The entry values in the matrix are the correlation coefficient values. For example, the value 0.5823 of the column ASD-Seed2-F4 and row F8 indicates that the activation of the seed F4 (frontal, right) has activated brain region F8 (frontal-temporal, right) of the participants belonging to the ASD group for the valid syllogism. The corresponding activation level in the case of invalid syllogism for the same group of participants is larger than the previous one (0.656).

By looking more carefully at the two heat maps, we observed that the level of activations on the other brain locations induced by seeds F3 and F4 are in general different in the case of valid syllogism than in case of invalid case, as well as between the group of participants. Therefore, the brain functional connectivity of ASD, ADHD, and control, formed by the activations of seeds F3 and F4 are different in the valid and invalid syllogism. We remind here that the choice of activation of F3 and F4 is based on literature review (Section 2.5.2) and especially on the empirical evidence it provides that these two regions are heavily involved in *emotion regulation* (as our results have shown) and *executive function,* i.e., the working memory intense involvement during varying cognitive loads, as is the case of valid and invalid reasoning in our study. 

From the above heat maps, we observe that in the ASD the activation of seed F3 induces on the rest of the regions with relatively similar and positive activations. Seed F4 in ASD also activates positively and strongly the region F8 both in valid and invalid case (0.582 and 0.655 correlations, respectively), while activates negatively regions AF3, T7, O2, P8, and AF4 with different strength in valid and invalid case. More specifically, AF4 in the invalid case is activated more negatively than in the invalid case, while AF3 more negatively in the valid case than in the invalid one. *In general, the distribution of the activated regions, positively or negatively, due to F3 and F4 seeds, is different both in respect to syllogism type and the group of participants.*

Based on these heat maps, the connectivity maps are plotted, using only the maximum values of the correlations. The seed-PLS based *functional connectivity maps* of the aforementioned figures reveal some very interesting, albeit ‘rough’, connectivity characteristics. We observe that seed F4 activates strongly and positively regions F8 both in valid and invalid cases, in ASD subjects. In ADHD, the F3 seed activates negatively region T8 in the valid case (−0.540), and region O2 in the invalid case (−0.532). In ADHD, seed F4 activates positively region T7 in the valid and O1 and T7 in the invalid case. In the case of control subjects, seed F3 activates positively T8 (0.509) and negatively O1 (−0.518) in valid case, while positively P8 (0.458), in invalid case. For the same group, seed F4 activates positively P7 (0.518) and negatively (−0.652) T8 in invalid case. *An interesting observation is also that seed F4 (frontal, right), in the case of ASD participants, in both valid and invalid syllogism, activates only one region, namely the region F8 (frontal-temporal, right).*

**Figure 24:**
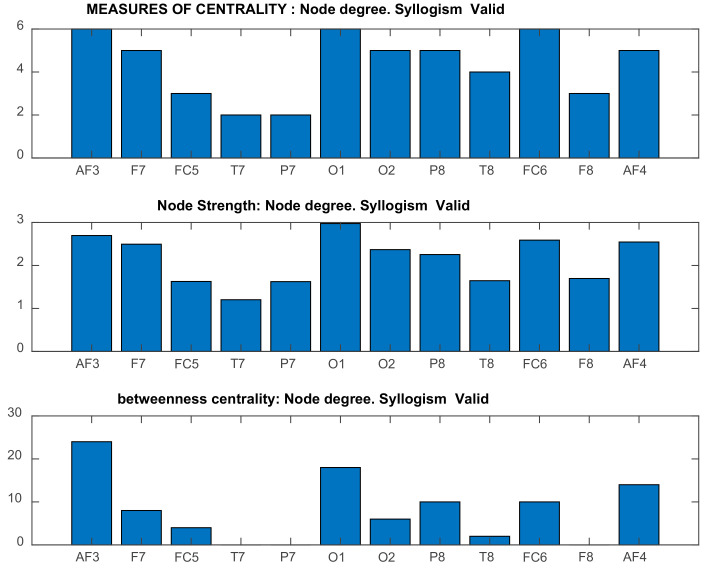
Measures of centrality of the network generated by the brain salience matrix V, for the valid syllogism. The rows and columns of V are the nodes of the network, while the links correspond to elements of the weight matrix W (normalized V and taking its elements with a threshold = 0.20, i.e., retaining the 20% of the strongest links).

**Figure 25:**
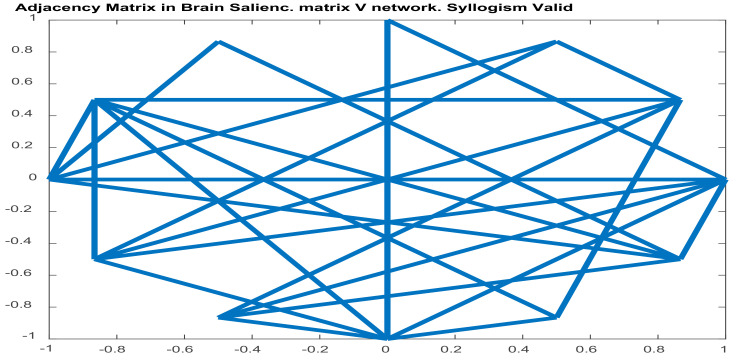
Adjacency matrix of the network generated by the brain salience matrix V, for the valid syllogism. The rows and columns of V are the nodes of the network, while the links correspond to elements of the weight matrix W (normalized V and taking its elements with a threshold = 0.20, i.e., retaining only the 20% of the strongest links). The density of the network is 0.400.

**Figure 26:**
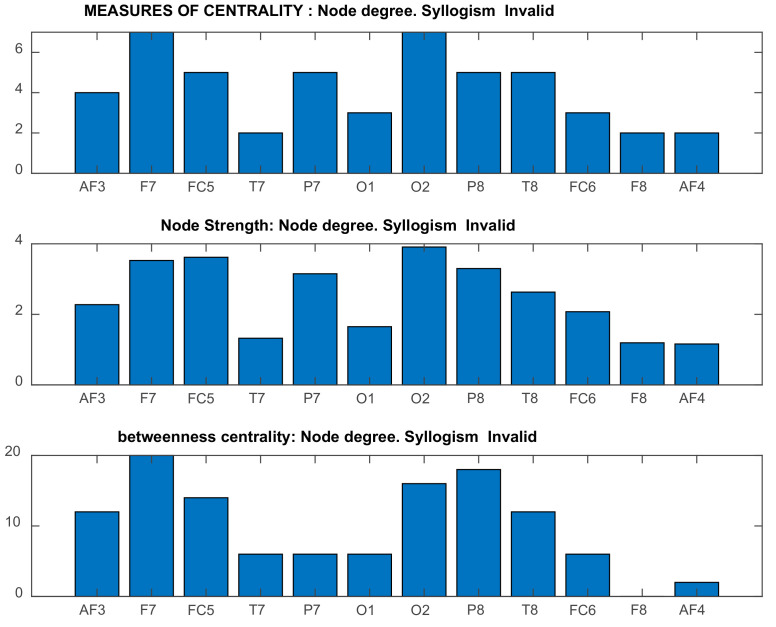
Measures of centrality of the network generated by the brain salience matrix V, for the invalid syllogism. The rows and columns of V are the nodes of the network, while the links correspond to elements of the weight matrix W (normalized V and taking its elements with a threshold = 0.20, i.e., retaining the 20% of the strongest links).

**Figure 27:**
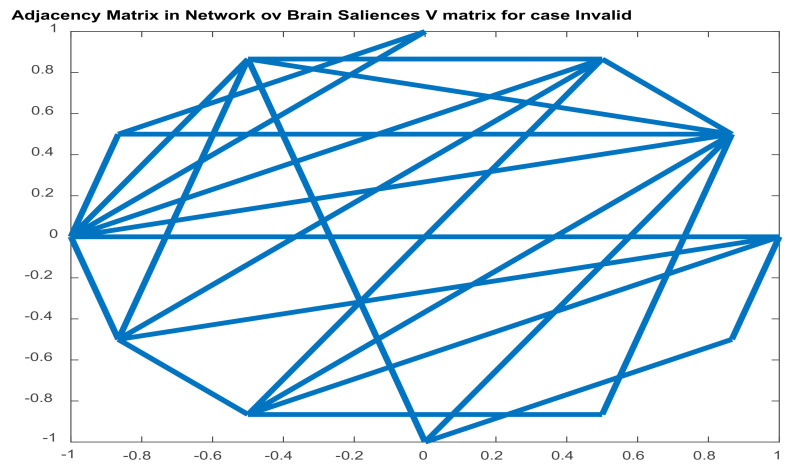
Adjacency matrix of the network generated by the brain salience matrix V, for the invalid syllogism. The rows and columns of V are the nodes of the network, while the links correspond to elements of the weight matrix W (normalized V and taking its elements with a threshold = 0.20). The density of the network is 0.370.

Figure 24 and Figure 25 present the results of computing the measures of *centrality and adjacency* matrix of the seed-based brain saliences, functional network, for the valid case and Figure 26 and Figure 27 for the invalid case. In the valid syllogism (Figure 24 and Figure 25), we observe that nodes 1, 6, and 10 have the highest degrees, followed by nodes 2, 7, 8, and 12, while node 6 has the highest strength followed by nodes 2, 7, 8, and 12. The network’s complexity is further depicted by its pictorial presentation of the adjacency matrix, Figure 25, indicating a more complex network compared with the one of Figure 27, for invalid case.

**Figure 28:**
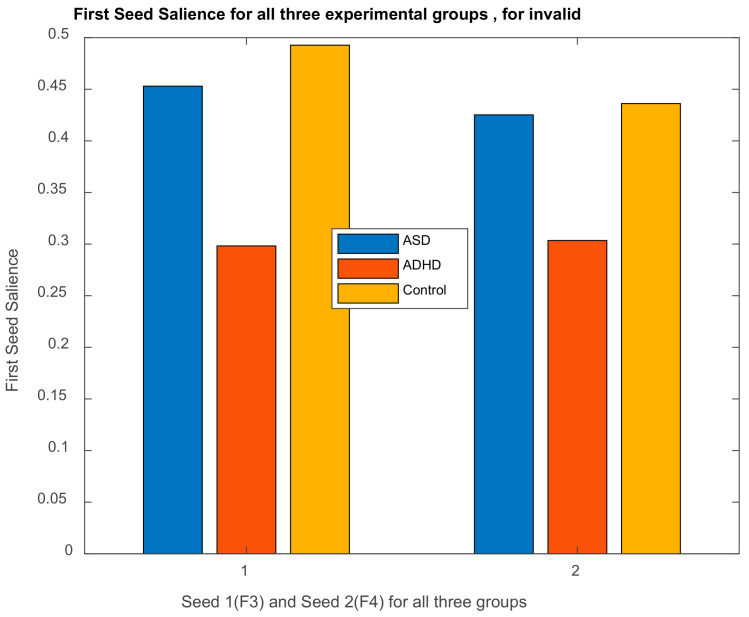
Plot of first seed salience, vs. seeds 1 and 2 (MSE values at F3 and F4, respectively), for all groups, in the invalid syllogism.

**Figure 29:**
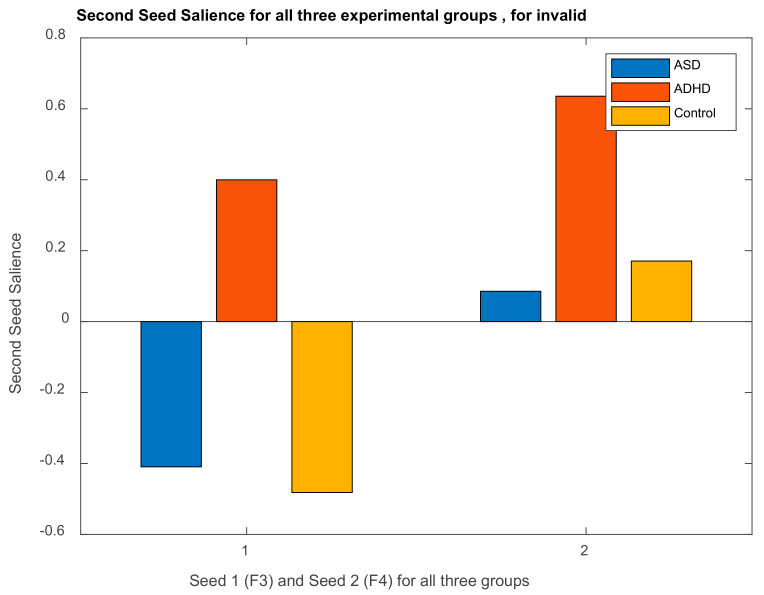
Plot of second seed salience, vs. seeds 1 and 2 (MSE values at F3 and F4, respectively), for all groups, in the invalid syllogism.

Figure 28 depicts the first seed salience vs. seeds 1 and 2 (MSE values at F3 and F4, respectively), for all groups, in the invalid syllogism. Figure 29 shows the plot of second seed salience, vs. seeds 1 and 2 (MSE values at F3 and F4, respectively), for all groups, in the invalid syllogism.

**Figure 30:**
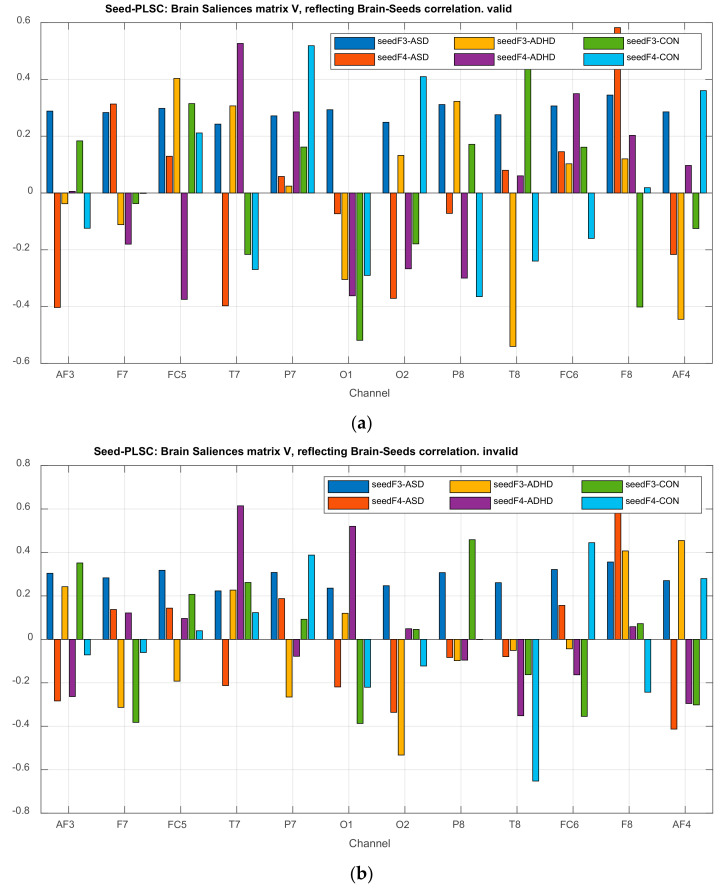
(**a**) Brain saliences matrix V reflecting the activated regions of the brain due to seed *regions F3 and F4 activations*, for the valid syllogism. (**b**) Brain saliences matrix V reflecting the activated regions of the brain due to seed *regions F3 and F4 activations*, for the invalid syllogism.

In Figure 30 we show the Brain saliences matrix V reflecting the activated regions of the brain due to seed *regions F3 and F4 activations*, for the valid syllogism, (**a**) , and the Brain saliences matrix V reflecting the activated regions of the brain due to seed *regions F3 and F4 activations*, for the invalid syllogism, (b).

**Figure 31:**
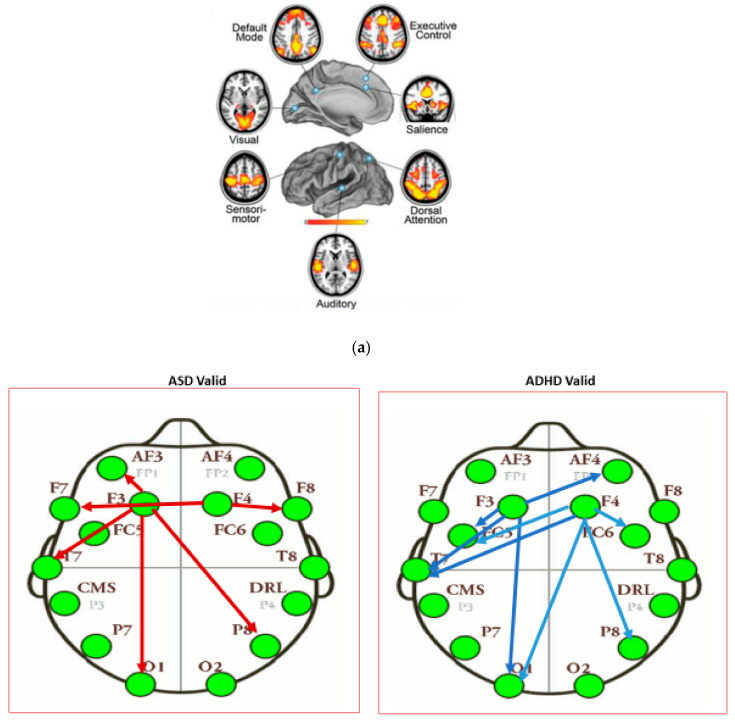

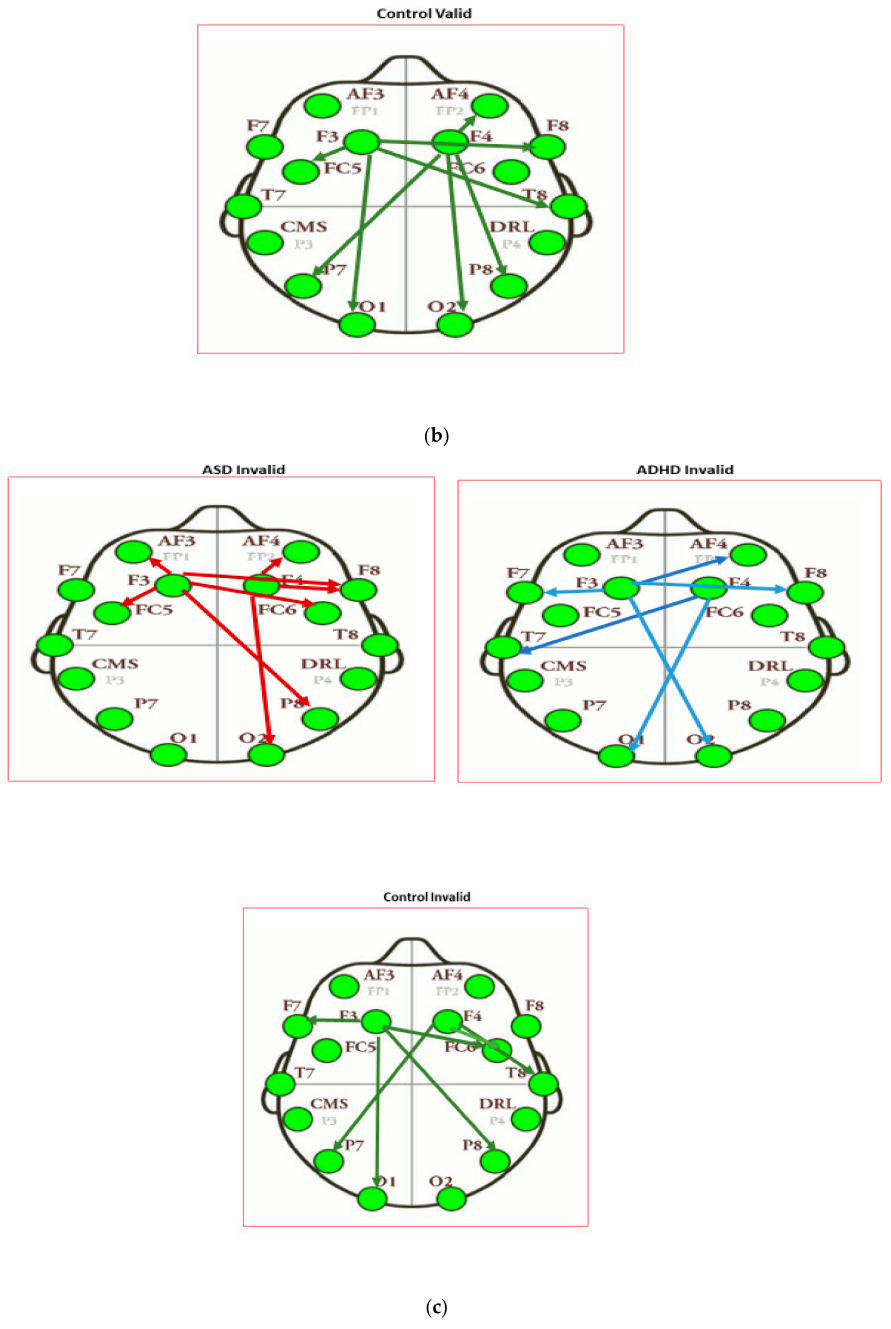
(**a**) The seven major brain functional networks (from Raichle M., 2001, freely available in ftp://imaging.wustl.edu/pub/raichlab/restless_brain, accessed on 8 November 2021) [148], used in this work to correspond our activated regions, due to seeds F3 and F4, with regions of the above major brain networks. (**b**) Connectivity network generated via seed-PLSC approach, when using electrodes F3 and F4 as seeds, for the case of valid syllogism. Lines of different colors correspond to different groups of participants, indicating which other electrodes are activated during the valid syllogism. The seeds have been chosen as the brain regions involved in *emotion regulation* and *goal-specific working memory* during a cognitive task (here the valid syllogism), based on the literature review. The lines connecting the F3 and F4 seed electrodes with the rest of electrodes are based on the seed brain salience matrix V heat map values (>0.3), Figure 22 and Figure 23, for valid and invalid cases, respectively. (**c**) Connectivity network generated via seed-PLSC approach, when using electrodes F3 and F4 as seeds, for the case of invalid syllogism. Lines of different colors correspond to different groups of participants, indicating which other electrodes are activated during the valid syllogism. The seeds have been chosen as the brain regions involved in *emotion regulation* and *goal-specific working memory* during a cognitive task (here the valid syllogism), based on the literature review. The lines connecting the F3 and F4 seed electrodes with the rest of electrodes are based on the seed brain salience matrix V heat map values (>0.3), Figure 22 and Figure 23, for valid and invalid cases, respectively.

Figure 31a shows seven known brain systems and their striking patterns of spatial coherence, extracted from the fluctuating patterns of intrinsic activity [148]. The above images were produced from the fluctuating patterns of intrinsic activity seen in the human brain with fMRI imaging. The patterns of spatial coherence shown were obtained by placing a *seed region* in the sensorimotor cortex. We see therefore how the activation of this seed region has activated regions in other systems. According to Raichle, there exist not only interactions *within a network* (i.e., among the elements of a single system, say DMN), but also *among networks*, for example between DMN and executive control. This emphasizes the *integrated nature* of the brain’s functional organization, which ‘incorporates’ or ‘blends’ both functionally related *intrinsic activity* (consuming major amount of brain’s energy) and *task-evoked* activity (adding little energy cost). It has to be emphasized here that the unique patterns of spatial coherence of the seven major brain systems shown in the figure tend to obscure the fact that these systems function in an integrated manner, not only during, say a cognitive task, but also during the resting state. 

This work inspired us to compare the spatial coherence of the regions activated by the seeds F3 and F4 in the present study, by corresponding the regions of Figure 31b,c to the regions of the above seven major brain functional regions. Comparing the Default Mode Network (DMN) with the emotive system of EEF recording electrode sites (Figure 2 and Figure 31), we make the following correspondence of the regions between them:

F3: Left dorsal lateral Prefrontal Cortex (l-DLPFC)
F4: Right dorsal lateral Prefrontal Cortex (r-DLPFC)
Middle distance between of AF3 and AF4: medial PFC (mPFC) (a proxy)
Middle distance between P7 and P8: Precuneus/posterior cingulate (PCC) (a proxy)
P7: Left lateral parietal (l-LP)
P8: Right lateral parietal (l-LP)
T7: Left inferior Temporal (l-infT) (T7 a proxy)
T8: Right inferior Temporal (r-infT) (T8 a proxy)

Table 12 provides the correspondence of the regions activated in our study to the regions of the major seven brain systems given in Raichle’s work [148]. 

From Table 12, we can observe that in both valid and invalid cases, ADHD and control subjects exhibit the same number of DMN-related activated regions, while ASD exhibits a largest number of DMN-related activated regions, especially in the invalid syllogism where both left and right anterio-frontal regions are involved. Therefore, within the DMN framework, ASD in general needs more resources (involves more regions) than ADHD and control to ‘handle’ the invalid syllogism cognitive demand, which seems to be more demanding than the valid one. This increased difficulty therefore resembles the difficulty encountered in passing from the one-back to two-back, etc. cognitive task, which involves ‘heavily’ executive function resources. Also, we see that the left and right parietal regions (P7, P8) are involved in both ADHD and control in both valid and invalid cases. Of note also, the ‘presence’ of the occipital regions O1 and O2, in both valid and invalid cases, however, related to the visual brain system. Both O1 and O2 are needed by control subjects to manage the valid syllogism and by ADHD to manage the invalid syllogism. ASD involves O1 for valid and O2 for invalid syllogism. Another point to note is that in valid syllogism, both ASD and ADHD need the ‘help’ of the left and right temporal regions, encountered in the *sensorimotor* and *auditory systems*, in both valid and invalid tasks, while ASD group does not need these regions in invalid syllogism. Strong presence, in both valid and invalid syllogism, exhibits the *dorsal attention system.* More specifically, in the valid case the control subjects and the ASD subjects in the invalid case involve a ‘large variety’ of regions of this system (frontal, parietal, occipital, and frontal-central). It seems that the dorsal attention system works here as a ‘background’ brain function activated continuously during the cognitive tasks of this experiment. Also, as expected, the left and right frontal temporal regions are activated, since during cognitive task the *executive control function* is heavily involved. 

As a conclusion of all the above, the valid and invalid cognitive tasks need the involvement of a large variety of brain regions, belonging to different major brain system suggested by Raichle [144], even though we focused initially on the DMN. Also, the ASD group seems to have the largest need for more sources (more brain regions activated) to handle the cognitive load during the cognitive tasks, and more importantly, this need is more intense for ASD subjects when passing from valid to invalid syllogism.

Table 13 and Table 14 contain the intensities of connections (values taken from heat maps, Figure 22 and Figure 23), shown on Figure 31b,c, of brain regions located in different and the same hemispheres. As we see, in ASD subjects, the connections of regions within the same hemispheres are stronger, on average, than the connections of regions in different hemispheres, for the invalid syllogism, a result which is in accordance with the work of Nowicka et al. [160], in which they report EEG evidence of altered patterns of brain activity and connectivity, in an experiment for name recognitions in autism. However, in the valid case, the ASD’s connections within the same hemispheres are weaker than those between the two hemispheres, a result different than that reported in the above-mentioned work. In the valid syllogism, the connections of regions located at different hemispheres are stronger than those of the ADHD subjects, which in turn are stronger than the ones of the ASD group.

The same ‘behavior’ is observed for the connections within the same brain hemispheres: on average, the connections of the control group, are stronger than those of ADHD, which are stronger than those in the ASD group. In both valid and invalid case, ADHD’s connections between regions in different hemispheres are, on average, stronger than the connections of ADHD subjects, located within the same hemispheres. Also in the invalid syllogism, ASD’s brain region connections in different hemispheres are, on average, weaker than the ones of the ADHD and control subjects. For regions within the same hemispheres, ASD’s connections are stronger, on average, than those in ADHD, and weaker than those in control group.

Two examples from the literature review of using DMN in combination with EEG in a cognitive task, the attentional processing, in ADHD and ASD group are given below. Very-low frequency (VLF) EEGs (0.02–0.20 Hz) are characteristics of the DMN, regions of which are more active during resting state, and DMN is considered to relate to an interconnected brain region consisting of anterior and posterior cingulate cortex (ACC, PCC), medial prefrontal cortex (mPFC), and precuneus [161]. A very interesting finding in this work is that VLF power of EEG attenuates when passing from the rest state to states related to cognitive tasks, as the attentional processing, with the same pattern in ADHD and control subjects. Broyd et al. [162] in a similar work found that the reduction of VLF power, exhibited from passing from rest to a cognitive task is more prominent in the mPFC for the control group, a result suggesting that the ADHD subjects do not ‘turn off’ typical DMN regions during cognitive tasks. Since valid–invalid syllogisms are in the same category of cognitive task as attentional processing, albeit not the same, it is natural to see how the DMN’s mPFC regions of the ADHD subjects compare with the ones in the healthy group. Although in our study we do not have measurements of the activation of mPFC regions of DMN in resting state, it is interesting to see their level of activation in the valid–invalid cognitive tasks, and how they compared with the ones of the control group. Our results for the activated AF4 (a proxy for mPFC) for ADHD and control, in both valid–invalid tasks are ‘consistent’ somehow with the results of [158]. As we observe in Figure 22, the activation of AF4 (mPFC) due to seed F3 in ADHD (−0.444) is stronger than that in control (−0.125) and also of the right direction since when the activation level of seed F3 is positive, the activation of AF4 is negative, revealing the attenuation process when passing from an ‘easy job’ to a more demanding one. This result suggests that in ADHD subjects, the DMN regions are always ‘turn-on’ (more active) during the valid–invalid syllogism, compared with the DMN regions of the control subjects. This finding is further enhanced by looking at Table 14, in which the AF4 region of the DMN system is present (i.e., activated) only in ADHD and not in the control group, indicating that the attenuation of the EEG power (in our case the reduction of MSE value or complexity) of the mPFC is more prominent in control subjects during the valid–invalid syllogism. 

For the same attention processing task, higher gamma power EEG was found in ASD subjects, in response to non-targets, in the left frontal AF3, bilateral parietal (P7, P8), and occipital (O1, O2) scalp channels of the DMN system [163]. In our study, regions AF3 and F8 were found activated by the seed F3, in the valid syllogism case.

## 4. Discussion and Conclusions

The analysis of the dynamics of brain–behavior interaction, especially during cognitive tasks is a very important and hot research topic, since its results can provide valuable and very crucial information on how highly activated brain regions are associated with behavioral characteristics of the subjects involved in cognitive tasks. Although a good number of applications of behavioral- and seed-PLSC methods have been applied on fMRI and PET data as the literature review shows, no application on EEG recordings seems to exist, to the best of our knowledge. Even more absent in the current literature is the application of these two approaches on EEG recordings taken from ASD and ADHD subjects. In this work, we used a very effective combination of the two approaches together with a nonlinear method for extracting all hidden nonlinearities from EEGs (the multiscale entropy), which have been recorded during ‘peculiar’ cognitive tasks, Aristotle’s syllogisms. 

Behavior-PLSC was used in our study to detect the correlation between two sets of data, dynamics of brain activity as measured by MSE values based on the EEGs, and behavioral measures as level of emotional state, degree of confidence in providing answers and age. Seed-PLSC were also used to build a ‘primitive’ *functional connectivity network*, within the *DMN brain system*, that was used to compare the changes in the functional connectivity of the ASD, ADHD, and control subjects’ brain regions, ‘imposed’ by the seed-activated regions F3 and F4 (related to l-DLPFC and r-DLPFC) on the mPFC and parietal-temporal regions. Based on the results of the analysis described in this work, the differences in the EEGs complexity measured by MSE between the three groups of participants lead to the conclusion that cortical information processing is changed in ASD and ADHD adults, therefore their level of cortical activation may be insufficient to meet the peculiar cognitive demands of Aristotle’s reasoning. These deficiencies are also accompanied by statistically significant differences in the behavioral response of ASD and ADHD, compared with those of control subjects. Specifically, the first behavior salience extracted by PLSC reveals the difference between ADHD and other participants for age as well as the difference between healthy (control) and the other groups for emotional state. The second behavioral salience reveals the difference between control subjects and ASD and ADHD for age (the maturation effect) and the difference (albeit smaller) between control and other groups for the emotional state. It reveals also the strong difference of ASD from ADHD and control subjects in the percent of confidence in answering questions in Aristotle’s valid–invalid syllogisms. The *changes in the functional connectivity* were found to be significant between the three groups. 

The contribution of this work lies in the application of the aforementioned combination: behavior-seed-PLSC methods, the MSE, and the Aristotle’s syllogism (a ‘composite tool’) to distinguish the subjects of the ASD, ADHD, and control groups. The tool seems capable of capturing the linear and nonlinear interaction of brain dynamics and behavioral characteristics of the subjects.

The results seem promising in adopting this type of reasoning, in the future, after further enhancements and experimental tests, as a *supplementary instrument* towards examining the differences in brain activity and behavioral responses of ASD and ADHD patients. The application of the combination of these two methods could be considered as a new tool of analysis in helping detecting more effectively such type of disorders. A linear measure alone, as the typical Power Spectrum Decomposition, is not capable of making such a distinction. The work also contributes in shedding light on the connection of the neural mechanisms of syllogism/reasoning of Aristotelian type with behavioral responses, contributing to understanding how humans reason logically and why ‘pathological’ subjects deviate from the norms of formal logic.

Several limitations of the present work should be mentioned. The first one refers to our findings that are based on cross-sectional data regarding the EEGs as well as the behavioral measures time series. Our results may be different from the ones based on longitudinal data; however, as it is well known, collecting such data it is not an easy job, since to obtain longitudinal data over such a wide age range as well as from different groups of participants is practically very difficult. Another point is that, we investigated behavioral changes in the functional and structural DMNs, which is a resting-state network. It would therefore be very crucial to observe the functional changes of other networks of the brain that are activated during demanding cognitive loads, like Aristotle’s syllogism in our case. For example, the visual and auditory networks that are also sensitive to cognitive tasks could also be used as reference networks, the changes of which could distinguish the performance of the three groups. The size of the samples (21 per each group) may be also a concern. Nevertheless, our sample size is comparable to similar PLS studies [3,4,5]. Finally, it would be very important and informative to study the interaction between brain dynamics and a number of other behavioral characteristics instead of the emotional state and degree of confidence in answering syllogistic questions, as used in this study. This could further assess the effectiveness of our composite tool in distinguishing the participants of the three groups. Finally, the found ‘superiority’ of Aristotle’s syllogism in combination with MSE and behavior-seed-PLSC over the typical ‘main-stream’ N-back tasks in distinguishing the gradual difficulty of ASD and ADHD in handling cognitive loads of such type, must be more extensively and rigorously assessed in a separate experiment. However, all these limitations could be analyzed in a future work. 

## Figures and Tables

**Table 1:** Channel index, name, and location.

Channel Index	Channel Name	Location
1	AF3	Anterio-frontal, left
2	F7	Frontal-temporal, left
3	F3	Frontal, left
4	FC5	Frontal-central, left
5	T7	Temporal, left
6	P7	Parietal, left
7	01	Occipital, left
8	02	Occipital, right
9	P8	Parietal, right
10	T8	Temporal, right
11	FC6	Frontal-central, right
12	F4	Frontal, right
13	F8	Frontal-temporal, right
14	AF4	Anterio-frontal, right

**Table 2:** Example of Aristotle Invalid Syllogism.

A	B
Denying the Antecedent	Affirming the Consequent
*Major Premise*: If A then B *Minor Premise*: not *Conclusion*: Therefore not B	*Major Premise*: If A then B *Minor Premise*: B *Conclusion:* Therefore A
Example If George beat the game already, then he is a great gamer. George did not beat the game already Therefore, George is not a great gamer	Example If George beat the game already, then he is a great gamer. George is a great gamer Therefore, George beat the game already

**Table 3:** Data used in the study for both valid and invalid syllogisms.

Study	Brain Activity Data	Demographic &Behavioral Data
63 participants (21 for each group, ASD, ADHD, and control)	Average MSE at 28 channel -syllogism combinations, per subject	Age, % of certainty in answers, mean of emotional state.

**Table 4:** Results of mixed ANOVA to test the *between-subjects effects* of group factor (ASD, ADHD, and control) for MSE values (dependent variables).

Tests of Between-Subjects Effects						
Transformed Variable: Average						
Source	Type III Sum of Squares	df	Mean Square	F	Sig.	Partial Eta Squared
Intercept	937.863	1	937.863	3881.298	0.000	0.985
group	1.722	2	0.861	3.564	0.034	0.106
Error	14.498	60	0.242			

**Table 5:** Results of mixed ANOVA to test the *within-subjects effects* for MSE values (dependent variables).

Tests of Within-Subjects Effects							
Source		Type III Sum of Squares	df	Mean Square	F	Sig.	Partial Eta Squared
Channels	Sphericity Assumed	1.210	13	0.093	12.677	0.000	0.174
	Greenhouse-Geisser	1.210	7.816	0.155	12.677	0.000	0.174
	Huynh-Feldt	1.210	9.397	0.129	12.677	0.000	0.174
	Lower-bound	1.210	1.000	1.210	12.677	0.001	0.174
Channels*group	Sphericity Assumed	0.511	26	0.020	2.677	0.000	0.082
	Greenhouse-Geisser	0.511	15.631	0.033	2.677	0.001	0.082
	Huynh-Feldt	0.511	18.794	0.027	2.677	0.000	0.082
	Lower-bound	0.511	2.000	0.256	2.677	0.077	0.082
Error (Channels)	Sphericity Assumed	5.728	780	0.007			
	Greenhouse-Geisser	5.728	468.940	0.012			
	Huynh-Feldt	5.728	563.832	0.010			
	Lower-bound	5.728	60.000	0.095			
Syllogism	Sphericity Assumed	0.092	1	0.092	2.263	0.138	0.036
	Greenhouse-Geisser	0.092	1.000	0.092	2.263	0.138	0.036
	Huynh-Feldt	0.092	1.000	0.092	2.263	0.138	0.036
	Lower-bound	0.092	1.000	0.092	2.263	0.138	0.036
Syllogism*group	Sphericity Assumed	0.080	2	0.040	0.985	0.379	0.032
	Greenhouse-Geisser	0.080	2.000	0.040	0.985	0.379	0.032
	Huynh-Feldt	0.080	2.000	0.040	0.985	0.379	0.032
	Lower-bound	0.080	2.000	0.040	0.985	0.379	0.032
Error(Syllogism)	Sphericity Assumed	2.441	60	0.041			
	Greenhouse-Geisser	2.441	60.000	0.041			
	Huynh-Feldt	2.441	60.000	0.041			
	Lower-bound	2.441	60.000	0.041			
Channels*Syllogism	Sphericity Assumed	0.020	13	0.002	0.983	0.466	0.016
	Greenhouse-Geisser	0.020	8.369	0.002	0.983	0.450	0.016
	Huynh-Feldt	0.020	10.173	0.002	0.983	0.458	0.016
	Lower-bound	0.020	1.000	0.020	0.983	0.325	0.016
Channels*Syllogism*Group	Sphericity Assumed	0.045	26	0.002	1.103	0.329	0.035
	Greenhouse-Geisser	0.045	16.737	0.003	1.103	0.347	0.035
	Huynh-Feldt	0.045	20.345	0.022	1.103	0.340	0.035
	Lower-bound	0.045	2.000	0.002	1.103	0.338	0.035
Error(Channels*Syllogism)	Sphericity Assumed	1.214	780	0.002			
	Greenhouse-Geisser	1.214	502.122	0.002			
	Huynh-Feldt	1.214	610.361	0.002			
	Lower-bound	1.214	60.000	0.020			

**Table 6:** Post hoc tests for each dependent variable. Pairwise comparisons for all combinations of the group factor (independent variable).

Multiple Comparisons							
						95% Confidence Interval	
	(I)group	(J)group	Mean Difference (I−J)	Std. Error	Sig.	Lower Bound	Upper Bound
LSD	ASD	ADHD	−0.075549196 *	0.0286686642	0.011	−0.132895063	−0.018203330
		normal	−0.027149080	0.0286686642	0.347	−0.084494947	0.0301996786
	ADHD	ASD	0.075549196 *	0.0286686642	0.011	0.018203330	0.132895063
		normal	0.048400116	0.0286686642	0.097	−0.008945751	0.105745983
	normal	ASD	0.027149080	0.0286686642	0.347	−0.030196786	0.084494947
		ADHD	−0.048400116	0.0286686642	0.097	−0.105745983	0.008945751
Bonferroni	ASD	ADHD	−0.075549169 *	0.0286686642	0.032	−0.146158774	−0.004939619
		normal	−0.027149080	0.0286686642	1.000	−0.097758658	0.043460497
	ADHD	ASD	0.075549196 *	0.0286686642	0.032	0.004939619	0.146158774
		normal	0.048400116	0.0286686642	0.290	−0.022209462	0.119009694
	normal	ASD	0.027149080	0.0286686642	1.000	−0.043460497	0.097758658
		ADHD	−0.048400116	0.0286686642	0.290	−0.119009694	0.022209462

*Note.* Based on observed means. The error term is Mean Square (Error) = 0.009. * The mean difference is significant at the 0.05 level.

**Table 7:** *X^2^* multidimensional test on behavioral data.

Emotion Dimension (SAM)	*X^2^* Value	df	Asympt. Sig. (2-Sided)
Arousal Emotion * group (Valid)	31.08	14	0.005
Arousal Emotion * education (Valid)	72.76	21	0.000
Control Emotion * Left or Right Hand	35.00	12	0.000

**Table 8:** Statistics of valence and arousal scores of participants that are used in plotting the 2-D circumplex model of affect, Figure 7c.

Valid Syllogism					
**Valence**	**Group**	**Mean**	**Median**	**Min**	Max
	ASD	4.14	4.00	1	8
	ADHD	4.29	4.00	1	9
	Control	3.00	2.00	1	7
Arousal	ASD	5.38	5.00	2	9
	ADHD	7.29	7.00	4	9
	Control	7.81	8.00	3	9
**Invalid Syllogism**					
**Valence**	**Group**	**Mean**	**Median**	**Min**	Max
	ASD	5.10	5.00	2	9
	ADHD	4.29	4.00	1	9
	Control	4.00	4.00	1	8
Arousal	ASD	5.62	5.00	2	9
	ADHD	6.76	7.00	3	9
	Control	7.62	8.00	3	9

**Table 9:** Squared singular values extracted from SVD on matrix *R*, and their cumulative percent of explaining the total variance of *R*, for valid and invalid syllogism.

Valid		Invalid	
Singular Value (Squared)	Cumulative % of Variance Explained	Singular Value (Squared)	Cumulative % of Variance Explained
4.185	70.30	6.73	77.65
0.608	80.52	0.67	85.38
0.509	89.07	0.46	90.75
0.336	94.72	0.39	95.30
0.157	97.35	0.16	97.21
0.074	98.60	0.10	98.44
0.047	99.40	0.08	99.44
0.022	99.77	0.04	99.96
0.013	100.00	0.00	100.00

**Table 10:** Notable high correlations (>0.40) between pair of brain–behavior measures (extracted from brain saliences matrix *V*).

Valid Syllogism			Invalid Syllogism		
Channel	Behavior-Group Interaction	Correlation Coefficient	Channel	Behavior-Group Interaction	Correlation Coefficient
AF3	Age*ADHD	−0.572	AF3	Confidence*Control	−0.562
FC5	Confidence*ADHDConfidence*Control	0.521 0.519	F7	Emotion*ADHD	−0.590
T7	Confidence*ASD	−0.715	F3	Emotion*ASD	0.473
P7	Confidence*ADHD	0.582	T7	Age*ADHD	0.641
O2	Emotion*ADHD	−0.640	P7	Age*Control	0.551
T8	Age*Control	0.600	O1	Confidence*ADHD	−0.408
F4	Emotion*Control	0.456	O2	Confidence*ASD	0.528
F8	Confidence*Control	−0.434	T8	Confidence*ADHD	−0.561
AF4	Emotion*ASD	0.547	FC6	Emotion*ADHD	0.490
			F4	Emotion*Control	0.746
			F8	Emotion*ASD	−0.574

**Table 11:** (**a**) Singular values and explained variance from seed-PLS for the valid syllogism. (**b**) Singular values and explained variance from seed-PLS for the invalid syllogism.

(**a**)	
Valid Syllogism	
Squared Singular Values	Total Variance Explained, %
41.183	98.43
0.330	99.22
0.197	99.69
0.105	99.94
0.019	99.99
0.002	100.00
(**b**)	
**Invalid Syllogism**	
**Squared Singular Values**	**Total Variance Explained, %**
28.476	94.44
0.903	97.43
0.370	98.66
0.224	99.41
0.100	99.74
0.076	100.00

**Table 12:** Linking major brain systems’ regions [144] to activated regions (channels) of the present study.

	Valid Syllogism			Invalid Syllogism		
Brain Network	ASD	ADHD	Control	ASD	ADHD	Control
Default Mode	AF4, P8	AF4	P7, P8	AF3, AF4, P8	AF4	P7, P8
Visual	O1	O1	O1, O2	O2	O1, O2	O1
Sensorimotor	T7	T7	T8		T7	T8
Auditory	T7	T7	T8		T7	T8
Dorsal Attention	O1, P8	O1, P8, T7	P7, P8, O1, O2, T8	O2, P8, FC5, FC6	T7, O1, O2	FC6, T7, O1, P8, T8
Salience	T7	T7	T8		T7	T8
Executive Control	F7, F8	F8	F8	F7	F7, F8	F7

**Table 13:** Intensity of connections of brain regions located in different and in the same hemispheres for the **valid** syllogism.

Intensity of Connections of Brain Regions Located in Different Hemispheres		
**ASD Valid**	**ADHD Valid**	**Control Valid**
AF3 to P8: 0.311	F3 to AF4: −0.446	F3 to T8: 0.509
F4 to F7: 0.313	F4 to FC5: −0.374	F3 to F8: −0.401
	F4 to O1: −0.361	F4 to P7: 0.5186
**Intensity of Connections of Brain Regions Located in the Same Hemispheres**		
F3 to AF3: 0.288	F3 to FC5: 0.403	F3 to FC5: 0.314
F3 to F7: 0.283	F3 to T7: 0.306	F3 to O1: −0.518
F3 to T7: 0.242	F3 to O1: −0.304	F4 to AF4: 0.360
F3 to O1: 0.293	F4 to FC6: 0.319	F4 to O2: 0.409
F4 to F8: 0.582	F4 to P8: −0.299	F4 to P8: −0.364

**Table 14:** Intensity of connections of brain regions located in different and in the same hemispheres for the **Invalid** syllogism.

Intensity of Connections of Brain Regions Located in Different Hemispheres		
**ASD Invalid**	**ADHD Invalid**	**Control Invalid**
F3 to P8: 0.307	F3 to O2: −0.532	F3 to P8: 0.458
F3 to FC6: 0.321	F3 to AF4: 0.454	F3 to FC6: −0.354
F3 to F8: 0.356	F3 to F8: 0.407	F4 to P7: 0.387
	F4 to T7: 0.614	
	F4 to O1: 0.520	
**Intensity of Connections of Brain Regions Located in the Same Hemispheres**		
F3 to AF3: 0.304	F3 to F7: 0.137	F3 to F7: −0.382
F3 to FC5: 0.317	F4 to F8: 0.058	F3 to O1: −0.387
F4 to AF4: −0.413		F4 to T8: −0.652
F4 to F8: 0.656		F4 to FC6: 0.445
F4 to O2: −0.336		

## Data Availability

The data that supported the findings of the present work are available from the corresponding authors, [A.P., G.P.], upon reasonable request.

## Supplementary Materials

The following are available online at www.mdpi.com/article/10.3390/brainsci11111531/s1, Figure S1: The Self-assessment Manikin (SAM) instrument, Figure S2: Distribution of I_(total_perm) (10000 permutations), for the case of EEG, valid type of syllogism (**a**), Distribution of I_(total_perm) (10000 permutations), for the case of MSE feature, valid type of syllogism (**b**). Table S1: Demographics of group of participants, used in the mixed ANOVA and X2 analysis, Table S2: Descriptive statistics of all variables involved in the mixed ANOVA and X2 analysis, Table S3: MANOVA analysis, Results of multivariate tests of the channel*group and channels*syllogism interactions. Manova tests (e.g Wilk’s lamda) > 0.14 show large effects, Table S4: Results of the test of spherisity in the mixed ANOVA analysis. Since W=0.022 is significant, the reported F value is epsilon corrected, Table S5: Results of mixed ANOVA to test within-subjects effects, for MSE values, Table S6: Independent ANOVA ANALYSIS, as support to the mixed ANOVA, Table S7: Estimated marginal means for group factor, Table S8: Estimated marginal means for Channels factor, Table S9: Estimated marginal means for Syllogism factor, Table S10: Mixed ANOVA iteractions effects groups*channels, Table S11: Mixed ANOVA iteractions effects groups*syllogism, Table S12: Mixed ANOVA iteractions effects channels*syllogism, Table S13: Mixed ANOVA iteractions effects groups*channels*syllogism.
