# Supplementary material for: Differences in Performance of ASD and ADHD Subjects Facing Cognitive Loads in an Innovative Reasoning Experiment
Source: Brain Sci. 2021 Nov 18;11(11):1531. doi: 10.3390/brainsci11111531 (PMC8615740; doi:10.3390/brainsci11111531)
Supplement: Supplementary file 1 [file brainsci-11-01531-s001.zip › brainsci-1303085-supplementary.pdf]

SUPPLEMENTARY MATERIAL

Table S1. Demographics of group of participants, used in the mixed ANOVA and X^2 analysis

group

		Frequency	Percent	Valid Percent	Cumulative Percent
Valid	ASD	30	37.0	37.0	37.0
	ADHD	30	37.0	37.0	74.1
	normal	21	25.9	25.9	100.0
	Total	81	100.0	100.0	

gender

		Frequency	Percent	Valid Percent	Cumulative Percent
Valid	male	57	70.4	71.3	71.3
	female	23	28.4	28.7	100.0
	Total	80	98.8	100.0	
Missing	System	1	1.2		
Total		81	100.0		

education

		Frequency	Percent	Valid Percent	Cumulative Percent
Valid	likio	21	25.9	26.9	26.9
	aei_tei	46	56.8	59.0	85.9
	master	10	12.3	12.8	98.7
	phd	1	1.2	1.3	100.0
	Total	78	96.3	100.0	
Missing	System	3	3.7		
Total		81	100.0		

occupation

		Frequency	Percent	Valid Percent	Cumulative Percent
Valid	not_working	7	8.6	9.3	9.3
	freelancer	16	19.8	21.3	30.7
	student	35	43.2	46.7	77.3
	public_servant	4	4.9	5.3	82.7
	private_employee	13	16.0	17.3	100.0
	Total	75	92.6	100.0	
Missing	System	6	7.4		
Total		81	100.0		

smoking

		Frequency	Percent	Valid Percent	Cumulative Percent
Valid	yes	23	28.4	28.4	28.4
	no	58	71.6	71.6	100.0
	Total	81	100.0	100.0	

hand_L_R

		Frequency	Percent	Valid Percent	Cumulative Percent
Valid	left_handed	5	6.2	6.2	6.2
	right_handed	73	90.1	90.1	96.3
	both	3	3.7	3.7	100.0
	Total	81	100.0	100.0	

health_issues

		Frequency	Percent	Valid Percent	Cumulative Percent
Valid	yes	24	29.6	30.8	30.8
	no	54	66.7	69.2	100.0
	Total	78	96.3	100.0	
Missing	System	3	3.7		
Total		81	100.0		

health_treatment

		Frequency	Percent	Valid Percent	Cumulative Percent
Valid	yes	28	34.6	35.4	35.4
	no	51	63.0	64.6	100.0
	Total	79	97.5	100.0	
Missing	System	2	2.5		
Total		81	100.0		

Table S2. Descriptive statistics of all variables involved in the mixed ANOVA and X^2 analysis

Descriptive Statistics

	group	Mean	Std. Deviation	N
AF3_VALID	ASD	.719485498	.1488445645	21
	ADHD	.779432267	.0911077627	21
	normal	.738849453	.1051185133	21
	Total	.745922406	.1184178913	63
AF3_MSE_IVALID	ASD	.76173370	.086142267	21
	ADHD	.78106342	.071223308	21
	normal	.74472072	.118535001	21
	Total	.76250595	.093736334	63
F7_VALID	ASD	.66101720	.176104891	21
	ADHD	.75158884	.080655825	21
	normal	.73004188	.071889186	21
	Total	.71421598	.123637758	63
F7_MSE_INVALID	ASD	.71164674	.098174472	21
	ADHD	.76744320	.062959499	21
	normal	.73707679	.082469139	21
	Total	.73872224	.084322733	63
F3_VALID	ASD	.74330678	.168153710	21
	ADHD	.80175403	.071396813	21
	normal	.77049502	.124222285	21
	Total	.77185194	.127760732	63
F3_MSE_INVALID	ASD	.76518508	.097014729	21
	ADHD	.81229310	.061701308	21
	normal	.78038291	.129588648	21
	Total	.78595370	.100363961	63
FC5_VALID	ASD	.66574390	.178263660	21
	ADHD	.73872253	.086234516	21
	normal	.71631991	.116994168	21
	Total	.70692878	.134208494	63
FC5_MSE_INVALID	ASD	.67900118	.163453413	21
	ADHD	.74470196	.084148007	21
	normal	.70899978	.119055873	21
	Total	.71090097	.127309770	63
T7_VALID	ASD	.688174970	.1725729717	21
	ADHD	.763300846	.0778176488	21
	normal	.701809607	.0927572478	21
	Total	.717761808	.1241808834	63
T7_MSE_INVALID	ASD	.70948109	.128770882	21
	ADHD	.76744016	.061931910	21
	normal	.70080863	.086818324	21
	Total	.72590996	.099532620	63
P7_VALID	ASD	.656845681	.1688264685	21
	ADHD	.753774513	.0763321512	21
	normal	.698456634	.1190464078	21
	Total	.703025609	.1313286681	63
P7_MSE_INVALID	ASD	.66909361	.134075696	21
	ADHD	.77088122	.052354797	21
	normal	.71757367	.104729601	21
	Total	.71918284	.109440013	63
O1_VALID	ASD	.67746842	.153546275	21
	ADHD	.79761240	.057831743	21
	normal	.75176442	.093454845	21
	Total	.74228175	.118286905	63
O1_MSE_INVALID	ASD	.73453145	.085138458	21
	ADHD	.78233100	.100735026	21
	normal	.75390571	.104271266	21
	Total	.75692272	.097521633	63
O2_VALID	ASD	.555951748	.1723093273	21
	ADHD	.783208804	.0804716324	21
	normal	.653472469	.1668562183	21
	Total	.664211007	.1716173890	63
O2_MSE_INVALID	ASD	.60358048	.133158269	21
	ADHD	.77353357	.094662376	21
	normal	.63083348	.202464026	21
	Total	.66931585	.165763440	63
P8_VALID	ASD	.67986660	.175053988	21
	ADHD	.77855561	.065189169	21
	normal	.71597941	.135466922	21
	Total	.72480054	.137348880	63
P8_MSE_INVALID	ASD	.70916269	.092391200	21
	ADHD	.76814757	.063953884	21
	normal	.72086901	.179139483	21
	Total	.73272642	.122823264	63
T8_VALID	ASD	.65426342	.178755200	21
	ADHD	.72761438	.081061145	21
	normal	.70206929	.119838020	21
	Total	.69464903	.134160343	63
T8_MSE_INVALID	ASD	.68237969	.109755588	21
	ADHD	.72141155	.091342007	21
	normal	.71830863	.135250585	21
	Total	.70736662	.113123930	63
FC6_VALID	ASD	.70104052	.155847055	21
	ADHD	.76681494	.061444105	21
	normal	.72011523	.099499995	21
	Total	.72932356	.114114791	63
FC6_MSE_INVALID	ASD	.74800507	.088346476	21
	ADHD	.77489909	.049989883	21
	normal	.72441690	.108068642	21
	Total	.74910702	.086737961	63
F4_VALID	ASD	.687780494	.1880064839	21
	ADHD	.792041598	.0837398851	21
	normal	.721355829	.1651357416	21
	Total	.733725974	.1561394901	63
F4_MSE_INVALID	ASD	.72608702	.134834316	21
	ADHD	.79591200	.072872456	21
	normal	.73610346	.156215707	21
	Total	.75270083	.128122366	63
F8_VALID	ASD	.69137379	.198570362	21
	ADHD	.75089760	.095455238	21
	normal	.71105558	.102801581	21
	Total	.71777566	.140323706	63
F8_MSE_INVALID	ASD	.72323876	.106599986	21
	ADHD	.77679866	.045898090	21
	normal	.73726609	.108389946	21
	Total	.74576784	.093046053	63
AF4_VALID	ASD	.71219789	.183219027	21
	ADHD	.77302463	.073202788	21
	normal	.73648516	.119543232	21
	Total	.74056923	.133425294	63
AF4_MSE_INVALID	ASD	.74020188	.113225948	21
	ADHD	.77802337	.061885919	21
	normal	.73848394	.117685453	21
	Total	.75223640	.100881153	63

Table S3. MANOVA analysis, Results of multivariate tests of the channel*group and channels*syllogism interactions. Manova tests (e.g Wilk's lamda) > 0.14 show large effects.

Multivariate Tests[a]

Effect		Value	F	Hypothesis df	Error df	Sig.	Partial Eta Squared
Channels	Pillai's Trace	.834	18.599[b]	13.000	48.000	.000	.834
	Wilks' Lambda	.166	18.599[b]	13.000	48.000	.000	.834
	Hotelling's Trace	5.037	18.599[b]	13.000	48.000	.000	.834
	Roy's Largest Root	5.037	18.599[b]	13.000	48.000	.000	.834
Channels * group	Pillai's Trace	.725	2.145	26.000	98.000	.004	.363
	Wilks' Lambda	.383	2.276[b]	26.000	96.000	.002	.381
	Hotelling's Trace	1.331	2.405	26.000	94.000	.001	.400
	Roy's Largest Root	1.066	4.017[c]	13.000	49.000	.000	.516
Syllogism	Pillai's Trace	.036	2.263[b]	1.000	60.000	.138	.036
	Wilks' Lambda	.964	2.263[b]	1.000	60.000	.138	.036
	Hotelling's Trace	.038	2.263[b]	1.000	60.000	.138	.036
	Roy's Largest Root	.038	2.263[b]	1.000	60.000	.138	.036
Syllogism * group	Pillai's Trace	.032	.985[b]	2.000	60.000	.379	.032
	Wilks' Lambda	.968	.985[b]	2.000	60.000	.379	.032
	Hotelling's Trace	.033	.985[b]	2.000	60.000	.379	.032
	Roy's Largest Root	.033	.985[b]	2.000	60.000	.379	.032
Channels * Syllogism	Pillai's Trace	.165	.730[b]	13.000	48.000	.725	.165
	Wilks' Lambda	.835	.730[b]	13.000	48.000	.725	.165
	Hotelling's Trace	.198	.730[b]	13.000	48.000	.725	.165
	Roy's Largest Root	.198	.730[b]	13.000	48.000	.725	.165
Channels * Syllogism * group	Pillai's Trace	.452	1.102	26.000	98.000	.354	.226
	Wilks' Lambda	.592	1.106[b]	26.000	96.000	.351	.230
	Hotelling's Trace	.613	1.109	26.000	94.000	.348	.235
	Roy's Largest Root	.443	1.672[c]	13.000	49.000	.098	.307

a. Design: Intercept + group
Within Subjects Design: Channels + Syllogism + Channels * Syllogism

b. Exact statistic

c. The statistic is an upper bound on F that yields a lower bound on the significance level.

Table S4. Results of the test of spherisity in the mixed ANOVA analysis. Since W=0.022 is significant, the reported F value is epsilon corrected.

Mauchly's Test of Sphericity[a]

Measure: MEASURE_1

Within Subjects Effect	Mauchly's W	Approx. Chi-Square	df	Sig.	Epsilon[b]		
					Greenhouse-Geisser	Huynh-Feldt	Lower-bound
Channels	.022	210.972	90	.000	.601	.723	.077
Syllogism	1.000	.000	0	.	1.000	1.000	1.000
Channels * Syllogism	.030	194.669	90	.000	.644	.783	.077

Tests the null hypothesis that the error covariance matrix of the orthonormalized transformed dependent variables is proportional to an identity matrix.

a. Design: Intercept + group
Within Subjects Design: Channels + Syllogism + Channels * Syllogism

b. May be used to adjust the degrees of freedom for the averaged tests of significance. Corrected tests are displayed in the Tests of Within-Subjects Effects table.

Table S5. Results of mixed ANOVA to test within-subjects effects, for MSE values

Tests of Within-Subjects Effects

Measure: MEASURE_1

Source		Type III Sum of Squares	df	Mean Square	F	Sig.	Partial Eta Squared
Channels	Sphericity Assumed	1.210	13	.093	12.677	.000	.174
	Greenhouse-Geisser	1.210	7.816	.155	12.677	.000	.174
	Huynh-Feldt	1.210	9.397	.129	12.677	.000	.174
	Lower-bound	1.210	1.000	1.210	12.677	.001	.174
Channels * group	Sphericity Assumed	.511	26	.020	2.677	.000	.082
	Greenhouse-Geisser	.511	15.631	.033	2.677	.001	.082
	Huynh-Feldt	.511	18.794	.027	2.677	.000	.082
	Lower-bound	.511	2.000	.256	2.677	.077	.082
Error(Channels)	Sphericity Assumed	5.728	780	.007			
	Greenhouse-Geisser	5.728	468.940	.012			
	Huynh-Feldt	5.728	563.832	.010			
	Lower-bound	5.728	60.000	.095			
Syllogism	Sphericity Assumed	.092	1	.092	2.263	.138	.036
	Greenhouse-Geisser	.092	1.000	.092	2.263	.138	.036
	Huynh-Feldt	.092	1.000	.092	2.263	.138	.036
	Lower-bound	.092	1.000	.092	2.263	.138	.036
Syllogism * group	Sphericity Assumed	.080	2	.040	.985	.379	.032
	Greenhouse-Geisser	.080	2.000	.040	.985	.379	.032
	Huynh-Feldt	.080	2.000	.040	.985	.379	.032
	Lower-bound	.080	2.000	.040	.985	.379	.032
Error(Syllogism)	Sphericity Assumed	2.441	60	.041			
	Greenhouse-Geisser	2.441	60.000	.041			
	Huynh-Feldt	2.441	60.000	.041			
	Lower-bound	2.441	60.000	.041			
Channels * Syllogism	Sphericity Assumed	.020	13	.002	.983	.466	.016
	Greenhouse-Geisser	.020	8.369	.002	.983	.450	.016
	Huynh-Feldt	.020	10.173	.002	.983	.458	.016
	Lower-bound	.020	1.000	.020	.983	.325	.016
Channels * Syllogism * group	Sphericity Assumed	.045	26	.002	1.103	.329	.035
	Greenhouse-Geisser	.045	16.737	.003	1.103	.347	.035
	Huynh-Feldt	.045	20.345	.002	1.103	.340	.035
	Lower-bound	.045	2.000	.022	1.103	.338	.035
Error (Channels*Syllogism)	Sphericity Assumed	1.214	780	.002			
	Greenhouse-Geisser	1.214	502.122	.002			
	Huynh-Feldt	1.214	610.361	.002			
	Lower-bound	1.214	60.000	.020			

Table S6. Independent ANOVA ANALYSIS, as support to the mixed ANOVA.

Tests of Between-Subjects Effects

Measure: MEASURE_1
Transformed Variable: Average

Source	Type III Sum of Squares	df	Mean Square	F	Sig.	Partial Eta Squared
Intercept	937.863	1	937.863	3881.298	.000	.985
group	1.722	2	.861	3.564	.034	.106
Error	14.498	60	.242			

Table S7. Estimated marginal means for group factor.

1. group

Measure: MEASURE_1

group	Mean	Std. Error	95% Confidence Interval	
			Lower Bound	Upper Bound
ASD	.695	.020	.654	.735
ADHD	.770	.020	.730	.811
normal	.722	.020	.682	.763

Table S8. Estimated marginal means for Channels factor.

2. Channels

Measure: MEASURE_1

Channels	Mean	Std. Error	95% Confidence Interval	
			Lower Bound	Upper Bound
1	.754	.012	.730	.778
2	.726	.012	.703	.750
3	.779	.013	.753	.805
4	.709	.016	.678	.740
5	.722	.012	.697	.747
6	.711	.013	.684	.738
7	.750	.011	.727	.772
8	.667	.017	.632	.702
9	.729	.015	.699	.759
10	.701	.014	.673	.729
11	.739	.011	.717	.762
12	.743	.017	.709	.777
13	.732	.014	.704	.759
14	.746	.014	.719	.774

Table S9. Estimated marginal means for Syllogism factor.

3. Syllogism

Measure: MEASURE_1

Syllogism	Mean	Std. Error	95% Confidence Interval Lower Bound	95% Confidence Interval Upper Bound
1	.722	.014	.693	.751
2	.736	.011	.715	.758

Table S10. Mixed ANOVA iteractions effects groups*channels.

4. group * Channels

Measure: MEASURE_1

group	Channels	Mean	Std. Error	95% Confidence Interval Lower Bound	95% Confidence Interval Upper Bound
ASD	1	.741	.021	.699	.783
	2	.686	.021	.645	.728
	3	.754	.023	.709	.799
	4	.672	.027	.618	.726
	5	.699	.022	.656	.742
	6	.663	.023	.617	.709
	7	.706	.019	.667	.745
	8	.580	.030	.519	.640
	9	.695	.026	.643	.746
	10	.668	.024	.619	.717
	11	.725	.019	.686	.763
	12	.707	.029	.648	.765
	13	.707	.024	.660	.755
	14	.726	.024	.678	.774
ADHD	1	.780	.021	.738	.822
	2	.760	.021	.718	.801
	3	.807	.023	.762	.852
	4	.742	.027	.688	.796
	5	.765	.022	.722	.809
	6	.762	.023	.716	.809
	7	.790	.019	.751	.829
	8	.778	.030	.718	.839
	9	.773	.026	.722	.825
	10	.725	.024	.676	.774
	11	.771	.019	.732	.810
	12	.794	.029	.735	.853
	13	.764	.024	.717	.811
	14	.776	.024	.728	.823
normal	1	.742	.021	.700	.784
	2	.734	.021	.692	.775
	3	.775	.023	.730	.821
	4	.713	.027	.659	.767
	5	.701	.022	.658	.745
	6	.708	.023	.662	.754
	7	.753	.019	.714	.792
	8	.642	.030	.582	.703
	9	.718	.026	.667	.770
	10	.710	.024	.661	.759
	11	.722	.019	.683	.761
	12	.729	.029	.670	.787
	13	.724	.024	.677	.771
	14	.737	.024	.690	.785

Table S11. Mixed ANOVA iteractions effects groups*syllogism

5. group * Syllogism

Measure: MEASURE_1

group	Syllogism	Mean	Std. Error	95% Confidence Interval	
				Lower Bound	Upper Bound
ASD	1	.678	.025	.629	.728
	2	.712	.019	.674	.749
ADHD	1	.768	.025	.719	.818
	2	.772	.019	.735	.810
normal	1	.719	.025	.670	.769
	2	.725	.019	.688	.762

Table S12. Mixed ANOVA iteractions effects channels*syllogism

6. Channels * Syllogism

Measure: MEASURE_1

Channels	Syllogism	Mean	Std. Error	95% Confidence Interval	
				Lower Bound	Upper Bound
1	1	.746	.015	.716	.776
	2	.763	.012	.739	.786
2	1	.714	.015	.684	.744
	2	.739	.010	.718	.760
3	1	.772	.016	.740	.804
	2	.786	.013	.761	.811
4	1	.707	.017	.673	.740
	2	.711	.016	.679	.743
5	1	.718	.015	.687	.748
	2	.726	.012	.702	.750
6	1	.703	.016	.671	.735
	2	.719	.013	.693	.745
7	1	.742	.014	.715	.770
	2	.757	.012	.732	.781
8	1	.664	.018	.627	.701
	2	.669	.019	.631	.707
9	1	.725	.017	.691	.758
	2	.733	.015	.702	.763
10	1	.695	.017	.661	.728
	2	.707	.014	.679	.736
11	1	.729	.014	.701	.758
	2	.749	.011	.728	.771
12	1	.734	.019	.695	.772
	2	.753	.016	.721	.785
13	1	.718	.018	.682	.753
	2	.746	.012	.723	.769
14	1	.741	.017	.707	.774
	2	.752	.013	.727	.778

Table S13. Mixed ANOVA iteractions effects groups*channels*syllogism.

7. group * Channels * Syllogism

Measure: MEASURE_1

group	Channels	Syllogism	Mean	Std. Error	95% Confidence Interval	
					Lower Bound	Upper Bound
ASD	1	1	.719	.026	.668	.771
		2	.762	.021	.721	.803
	2	1	.661	.026	.609	.713
		2	.712	.018	.676	.748
	3	1	.743	.028	.688	.799
		2	.765	.022	.722	.809
	4	1	.666	.029	.608	.724
		2	.679	.028	.624	.734
	5	1	.688	.027	.635	.741
		2	.709	.021	.667	.752
	6	1	.657	.028	.601	.712
		2	.669	.022	.624	.714
	7	1	.677	.024	.630	.725
		2	.735	.021	.692	.777
	8	1	.556	.032	.492	.620
		2	.604	.033	.538	.669
	9	1	.680	.029	.622	.738
		2	.709	.027	.656	.762
	10	1	.654	.029	.596	.712
		2	.682	.025	.633	.732
	11	1	.701	.025	.652	.750
		2	.748	.019	.711	.785
	12	1	.688	.033	.621	.754
		2	.726	.028	.671	.781
	13	1	.691	.031	.630	.753
		2	.723	.020	.683	.763
	14	1	.712	.029	.654	.770
		2	.740	.022	.696	.784
ADHD	1	1	.779	.026	.728	.831
		2	.781	.021	.740	.822
	2	1	.752	.026	.700	.804
		2	.767	.018	.731	.803
	3	1	.802	.028	.746	.857
		2	.812	.022	.769	.856
	4	1	.739	.029	.681	.797
		2	.745	.028	.690	.800
	5	1	.763	.027	.710	.816
		2	.767	.021	.725	.810
	6	1	.754	.028	.698	.809
		2	.771	.022	.726	.816
	7	1	.798	.024	.750	.845
		2	.782	.021	.740	.825
	8	1	.783	.032	.719	.847
		2	.774	.033	.708	.839
	9	1	.779	.029	.720	.837
		2	.768	.027	.715	.821
	10	1	.728	.029	.670	.786
		2	.721	.025	.672	.771
	11	1	.767	.025	.718	.816
		2	.775	.019	.738	.812
	12	1	.792	.033	.726	.859
		2	.796	.028	.741	.851
	13	1	.751	.031	.690	.812
		2	.777	.020	.737	.817
	14	1	.773	.029	.715	.831
		2	.778	.022	.734	.822
normal	1	1	.739	.026	.688	.790
		2	.745	.021	.704	.786
	2	1	.730	.026	.678	.782
		2	.737	.018	.701	.773
	3	1	.770	.028	.715	.826
		2	.780	.022	.737	.824
	4	1	.716	.029	.658	.774
		2	.709	.028	.654	.764
	5	1	.702	.027	.649	.755
		2	.701	.021	.659	.743
	6	1	.698	.028	.643	.754
		2	.718	.022	.673	.762
	7	1	.752	.024	.704	.799
		2	.754	.021	.712	.796
	8	1	.653	.032	.590	.717
		2	.631	.033	.565	.696
	9	1	.716	.029	.658	.774
		2	.721	.027	.668	.774
	10	1	.702	.029	.644	.760
		2	.718	.025	.669	.768
	11	1	.720	.025	.671	.769
		2	.724	.019	.687	.762
	12	1	.721	.033	.655	.788
		2	.736	.028	.681	.791
	13	1	.711	.031	.650	.772
		2	.737	.020	.697	.777
	14	1	.736	.029	.678	.795
		2	.738	.022	.694	.782

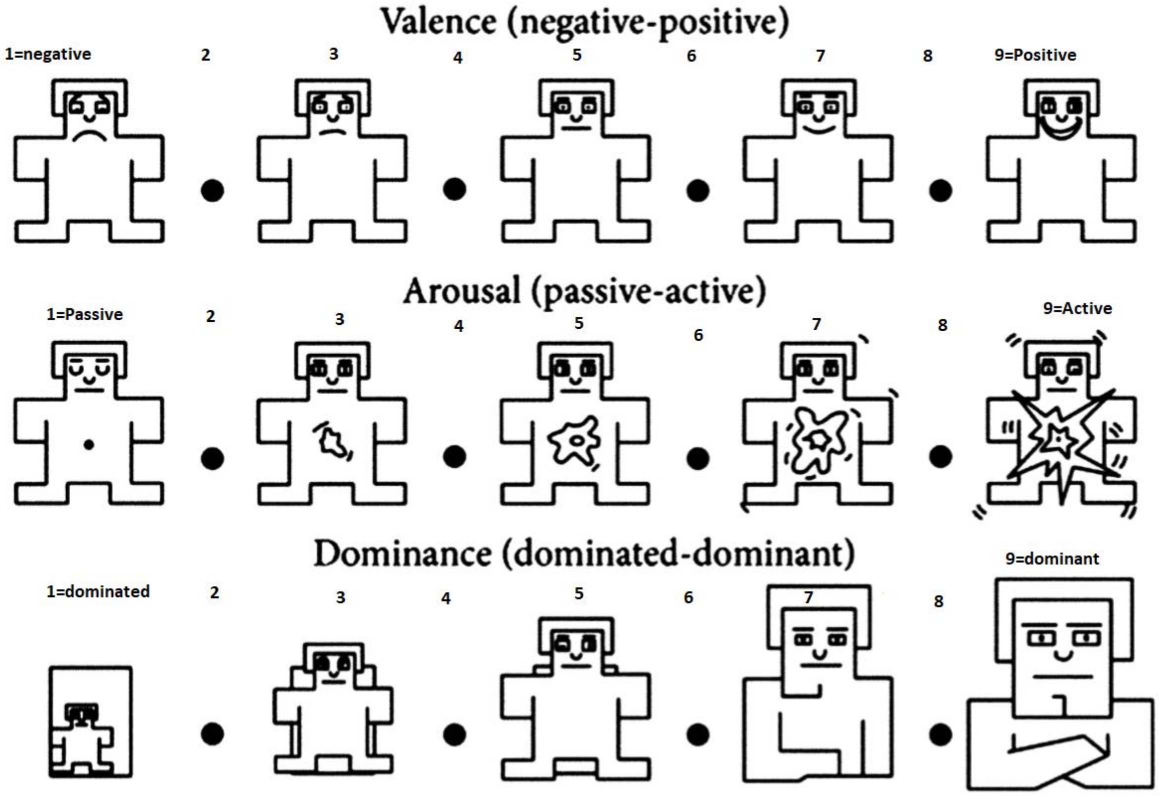

Figure S1. The Self-assessment Manikin (SAM) instrument for assessing the dimensions of emotion state: Valence, Arousal, and Dominance or Control. Numbers indicate the rate scale, with 2,4,6,8 the rates between two pictures, allowing a 9 point scare rating of a participant state of emotion *(Taken from Bradley et al., 1994, with minor modifications of one of the authors (AP) of the present paper).*

Results of the permutation tests

In this section we present the results of the permutation test, described in section 2.5.1. Figure S2,b below shows the distribution of $\boldsymbol{I_{total\_perm}}$ (10000 permutations), for the case of EEG, valid type of syllogism. The histogram has been normalized probability-wised so with this normalization, the height of each bar is equal to the probability of selecting an observation within that bin interval, and the height of all of the bars sums to 1. The value of deterministic I_{total} =1.5644 occurs with probability p=0.0326 < 0.05, so the extracted common information from the two Brain activity and behavioral tables is statistically significant. Figure S2,a shows the distribution of $\boldsymbol{I_{total\_perm}}$ (10000 permutations), for the case of MSE feature, valid of syllogism (similar results are for the invalid type). The histogram has been normalized probability-wised so with this normalization, the height of each bar is equal to the probability of selecting an observation within that bin interval, and the height of all of the bars sums to 1. The value of deterministic I_{total} =6.4841 occurs with probability p=0.046 < 0.05, so the extracted common information from the two Brain activity and behavioral tables is statistically significant. However, *this p value is closer to 5% than the one value found in the case of using the MSE feature, an indication that MSE extracts important and mainly nonlinear information from EEGs, allowing greater amount of common information to be extracted via PLSC, enhancing further the efficiency of this method.*

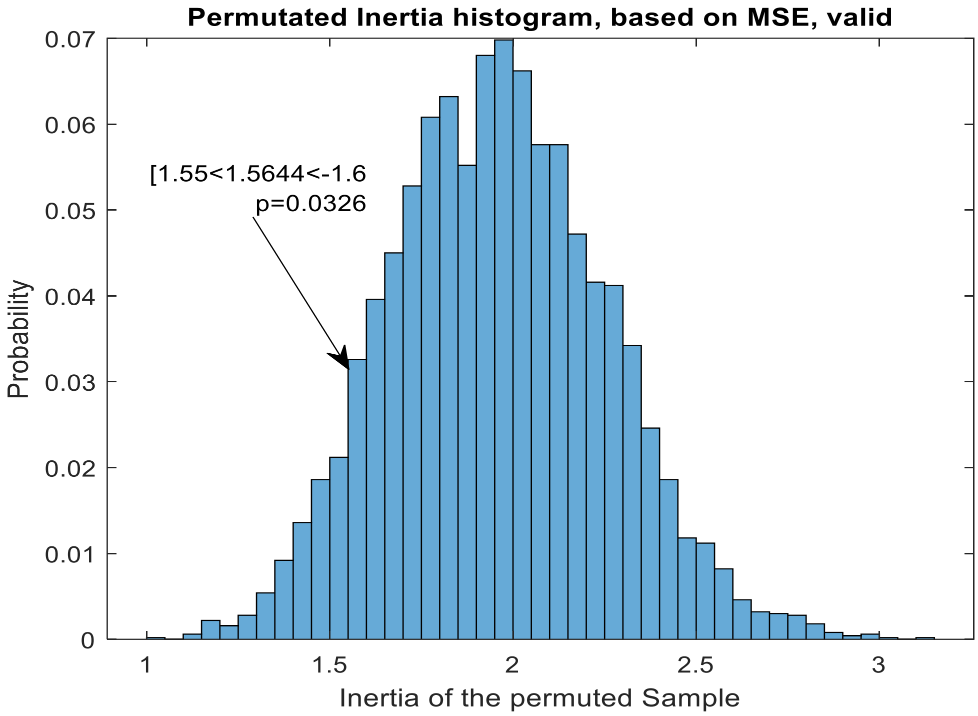

(a)

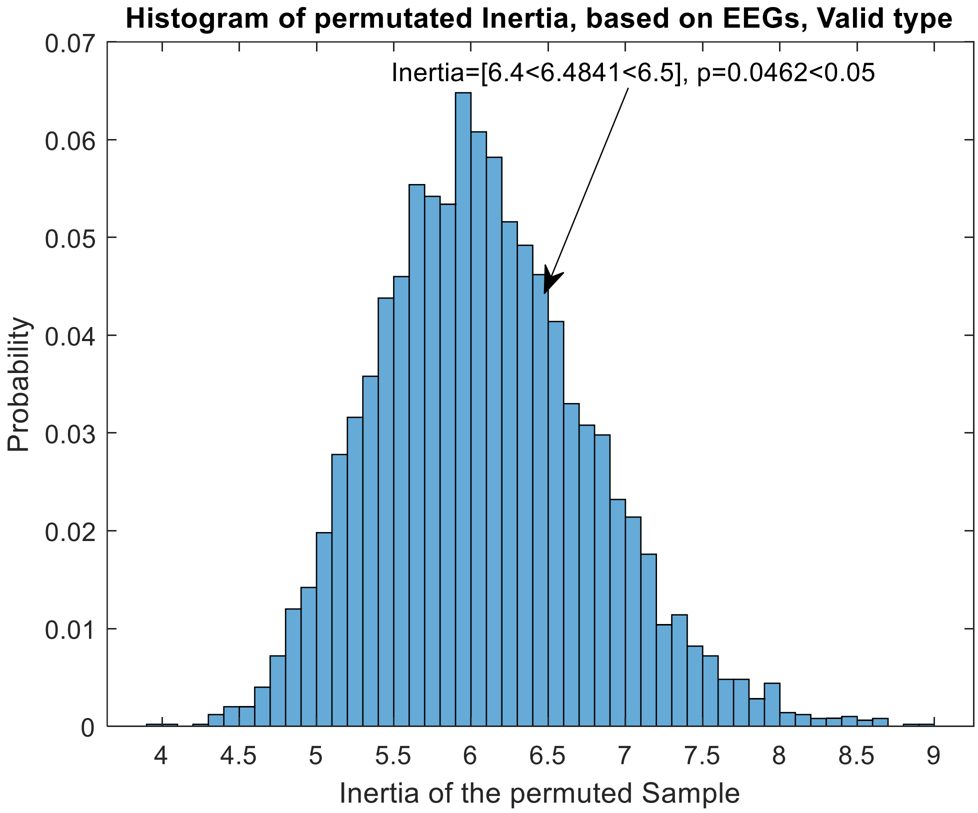

(b)

Figure S2. Distribution of I_(total_perm) (10000 permutations), for the case of EEG, valid type of syllogism (**a**), Distribution of I_(total_perm) (10000 permutations), for the case of MSE feature, valid type of syllogism (**b**).
